# Development of a Rapid Fluorescent Diagnostic System to Detect Subtype H9 Influenza A Virus in Chicken Feces

**DOI:** 10.3390/ijms22168823

**Published:** 2021-08-17

**Authors:** Hien Thi Tuong, Ju Hwan Jeong, Young Ki Choi, Hyun Park, Yun Hee Baek, Seon-Ju Yeo

**Affiliations:** 1 Zoonosis Research Center, Department of Infection Biology, School of Medicine, Wonkwang University, Iksan 54538, Korea; tuonghien23@gmail.com (H.T.T.); hyunpk@wku.ac.kr (H.P.); 2 Department of Microbiology, College of Medicine and Medical Research Institute, Chungbuk National University, Cheongju 28644, Korea; jeongbau07@naver.com (J.H.J.); choiki55@chungbuk.ac.kr (Y.K.C.); 3 Zoonotic Infectious Diseases Research Center, Chungbuk National University, Cheongju 28644, Korea; 4 Center for Study of Emerging and Re-emerging Viruses, Korea Virus Research Institute, Institute for Basic Science (IBS), Daejeon 34126, Korea; 5 Department of Tropical Medicine and Parasitology, College of Medicine, Seoul National University, Seoul 03080, Korea

**Keywords:** fecal specimen, subtype H9 influenza A virus, rapid fluorescent immunochromatographic test, monoclonal antibody

## Abstract

The circulation of the H9N2 virus results in significant economic losses in the poultry industry, and its zoonotic transmission highlights the need for a highly sensitive and rapid diagnostic and detection system for this virus. In this study, the performance of lateral flow test strips for a fluorescent immunochromatographic test (FICT) was optimized for the diagnosis of H9N2 virus-infected animal samples. The novel monoclonal antibodies (McAbs) against influenza A H9 viruses were developed, and two categories of McAbs with linear and conformational epitopes were compared for the performance of rapid diagnostic performance in the presence of feces sample at different time points (2, 4, and 6 days) post-infection (dpi). The limit of detection (LOD) of FICT and Kd values were comparable between linear and conformational epitope McAbs. However, superior performance of linear epitope McAbs pairs were confirmed by two animal studies, showing the better diagnostic performance showing 100% relative sensitivity in fecal samples at 6 dpi although it showed less than 80% sensitivity in early infection. Our results imply that the comparable performance of the linear epitope McAbs can potentially improve the diagnostic performance of FICT for H9N2 detection in feces samples. This highly sensitive rapid diagnostic method can be utilized in field studies of broiler poultry and wild birds.

## 1. Introduction

Avian influenza virus (AIV) H9N2 subtype infecting turkeys was first reported in the United States in 1966 [1] and has widely spread to Europe and Asia since the 1990s [2,3]. In China, the H9N2 subtype virus was isolated from diseased chickens in Guangdong province in 1994 and later infected domestic poultry in other provinces in China [2,4,5,6]. The first field outbreak of the H9N2 virus in Korea was reported in March 1996 caused by A/chicken/Korea/96006/96 (H9N2) with a 20% mortality rate and a severe (98.1%) drop in egg production. A genetically closely related virus to the A/duck/Hong Kong/Y439/97 (Korea group) virus was later isolated from aquatic birds. Since then, H9N2 viruses have been widespread in domestic poultry farms in Korea and have formed a unique cluster of Korean lineage [7,8].

Among the many subtypes, H9N2 is a low pathogenic avian influenza (LPAI) virus, which circulates primarily among wild birds and domestic poultry, resulting in great economic losses to the poultry industry because of declined egg production or moderate to high mortality [9,10,11,12]. If co-infected with other pathogens, approximately 70% morbidity and 30% mortality rates have been reported in poultry [13,14]. Moreover, the H9N2 LPAI viruses are able to recognize alpha 2–6-linked sialic acid for direct transmission to mammalian species, including swine and humans, raising human health concerns [15]. Until July 2020, a total of 59 cases of non-fatal human infection were reported in Asia and the Middle East (http://www.who.int/influenza/human_animal_interface/HAI_Risk_Assessment/en/ (accessed on 11 February 2021)). However, sero-epidemiological studies provided evidence of human exposure to H9N2 viruses, and the number of infected humans was suspected to be much greater than the number of reported cases [16,17,18,19,20]. Given the great economic losses, as well as the fear that H9N2 viruses may become a pandemic through repeated interspecies transmission from poultry to humans, efficient surveillance of H9N2 is essential.

An accurate and rapid diagnostic method to detect H9N2 viruses in poultry and humans is necessary to improve surveillance and pandemic preparedness. Currently, rapid diagnostic tests (RDTs) and real-time reverse transcription-polymerase chain reaction (rRT-PCR) vary in their sensitivity and specificity. According to CDC guidelines, hospitalized patients should be tested with high sensitivity and specificity molecular assays, such as rRT-PCR (90–95%), rather than RDTs (50–70%) [21]. Moreover, molecular methods also provide detailed information on specific influenza A virus subtypes and strains important for treatment. However, these methods are time-consuming and require instruments, highly trained personnel, and high costs.

Many recent trials have employed diagnosis platforms with fluorescent material to improve the sensitivity and specificity [22,23,24,25,26]. Previously, we developed a smartphone-based rapid fluorescent diagnostic system to detect the H9N2 virus in pathogen-free chickens [24]. However, this diagnosis targeted the internal nucleoproteins (NP) because of the expression of a highly conserved main protein of influenza viruses and, therefore, did not provide the subtype information. Several research groups currently focus on developing H9 subtype-specific diagnostic kits convenient for detecting H9 viruses during outbreaks [26,27]. Nevertheless, most of them lacked data for detecting the avian influenza virus in fecal samples using animal models. Following the respiratory infection, the H9N2 virus multiplies in the intestinal tract of chickens and is transmitted through feces [28,29]. In humans, detection of influenza virus RNA and influenza virus in the stool indicates localization of influenza virus in the gastrointestinal tract of children that could serve as an influenza virus transmission mode during seasonal and epidemic outbreaks [30]. Therefore, a high-performance rapid diagnostic system for fecal samples is essential for improving the efficient identification and management of influenza cases in poultry and humans.

In the current study, H9N2 virus-specific monoclonal antibody was developed and used to set up the fluorescent immunochromatographic strip test (FICT) that discriminates the hemagglutinin of H9 subtype AIV. We used an animal model to assess the ability of the FICT assay to detect H9N2 antigen in fecal specimens.

## 2. Results

### 2.1. Development of McAbs

**Figure 1:**
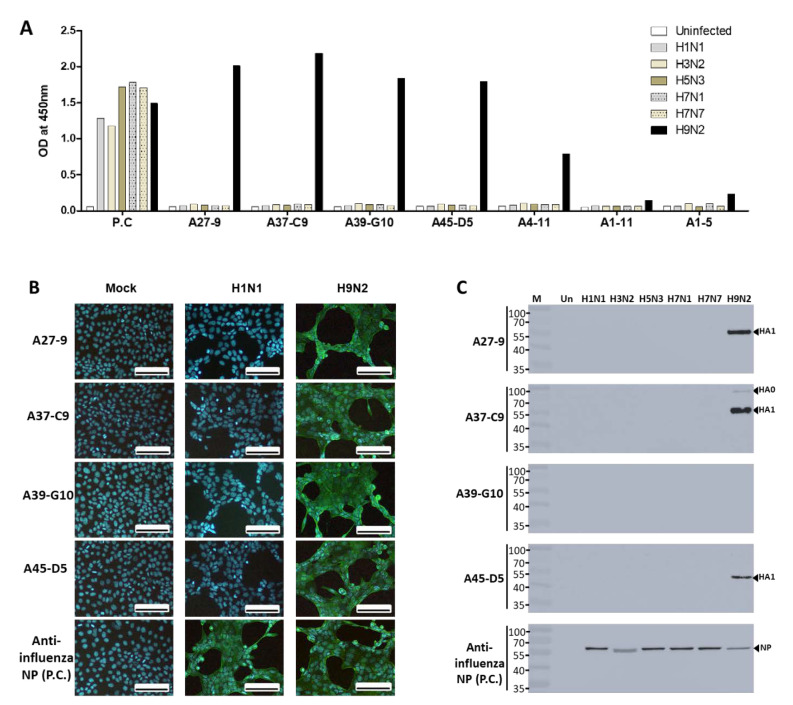
Characterization of McAbs against H9N2 virus by immunofluorescence studies. (**A**) Hybridoma screening with other avian influenza virus subtypes. P.C., anti-influenza NP. (**B**) Cells were infected with the virus for 12 h and fixed with 4% paraformaldehyde. After fixation, cells were incubated with A27-9, A37-C9, A39-G10, and A45-D5 antibodies and washed three times with PBST. Fluorescence was detected by a FICT-conjugated secondary antibody (scale bar, 100 μm; original magnification, 100×). Mock, non-infection. (**C**) Six influenza A subtype viruses were loaded on an SDS-PAGE gel, and Western blotting was used to analyze each antibody.

After immunization of mice with the inactivated H9N2 virus, 7 hybridoma cell lines producing McAbs were established. Indirect Enzyme-linked immunosorbent assay (ELISA) with 7 hybridoma cell lines producing McAbs was conducted for six avian influenza A virus subtypes at 1000 HAU/mL. Four hybridoma cell lines (A27-9, A37-C9, A39-G10, and A45-D5) reacted specifically with the H9N2 subtype with high signal and had no cross-reactivity with other subtype viruses. Reactivity of three hybridoma cell lines (A4-11, A1-11, A1-5) to the virus was relatively low from 0.3 to 0.8 OD. Anti-influenza A nucleoprotein (NP) (7307) was used as the positive control for confirming the presence of an equal amount of each subtype virus (Figure 1A). To further verify the specificity of McAbs, viral reactivity of McAbs was analyzed by Western blotting and immunofluorescence assay (IFA) (Figure 1B). Western blotting with 1000 HAU of viruses/lane was used to assess the ability of McAbs to recognize the linear epitope. Three McAbs (A27-9, A37-C9, A45-D5) reacted with H9N2 and not with other virus subtypes. The A39-G10 McAb did not react with linearized antigen, implying that its specific reactivity was due to the conformational structure of the target antigen (Figure 1C). In the IFA, the A27-9, A37-C9, A39-G10, A45-D5 McAbs reacted against the H9N2 subtype virus, and not against other viruses. The positive signal of anti-influenza NP showed the presence of the virus in both assays.

### 2.2. Selection of the Specific McAb Pair for FICT Assays

**Figure 2:**
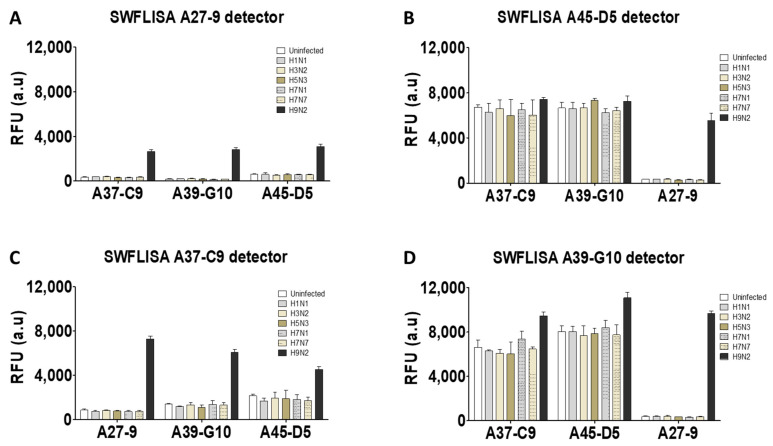
Selection of McAb pair for detection of H9N2 virus by sandwich FLISA. Four McAbs (A27-9, A37-C9, A39-G10, A45-D5) were conjugated to Europium-200 nm. (**A**) Europium A27-9 conjugate, (**B**) Europium A45-D5 conjugate, (**C**) Europium A37-C9 conjugate, (**D**) Europium A39-G10 conjugate were tested in pair with three remaining McAbs. The 2 pairs (A27-9/A39-G10; A27-9/A45-D5) reacted with the H9N2 virus with a high signal and discriminated from other subtype viruses.

Before the McAbs were applied to a rapid diagnostic strip, the specificity of antibody pairs was determined via the sandwich FLISA (Figure 2). After bio-conjugating, each McAb with Europium nanoparticles (Eu NP) was tested in pair with three remaining McAbs. The 96-well plate was coated with the anti-H9N2-specific antibodies and six influenza A subtype viruses (H1N1, H3N2, H5N3, H7N1, H7N7, and H9N2) were applied at 100 HAU/well, and the analytes were detected by measuring the fluorescence intensity. The two pairs of McAbs (A27-9, A39-G10 (Figure and A27-9, A45-D5) reacted with the virus at a high signal, discriminating H9N2 from other subtype viruses, and were selected for subsequent experiments.

### 2.3. Optimization of Lysis Buffer

**Figure 3:**
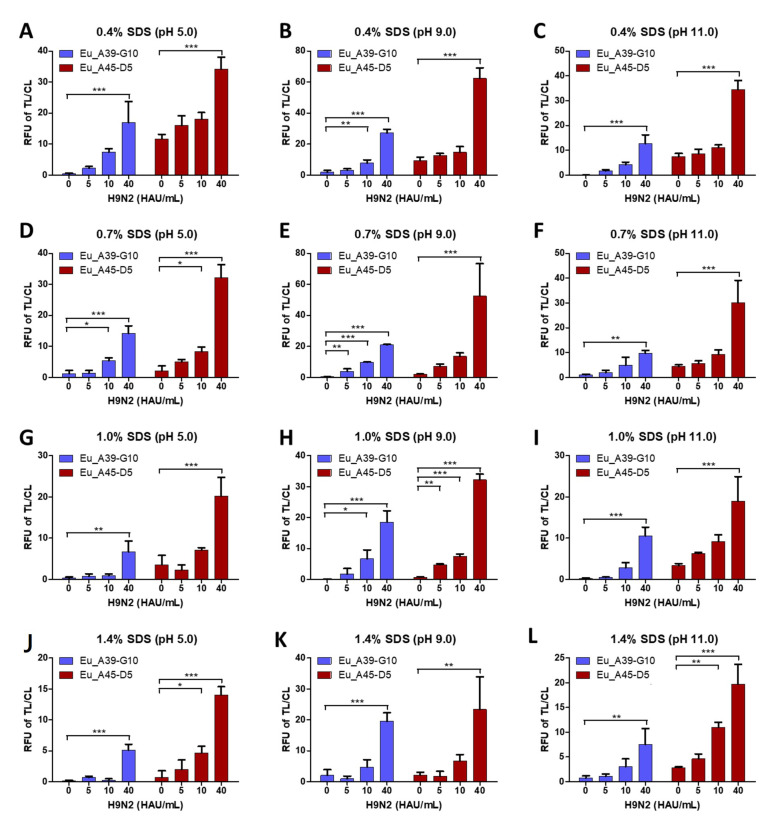
Optimization of lysis buffer for the FICT assay of Europium A45-D5 and Europium A39-G10. Various concentrations of SDS (0.4%, 0.7%, 1.0%, and 1.4%) at different pH values (5.0, 9.0, and 11.0) in basic lysis buffer (0.1 M Tris, 0.1 M EDTA, and 0.5% Triton X-100) were tested. In the lysis buffer, 0.4% SDS was dissolved at three different pHs (**A**–**C**). 0.7% SDS was dissolved in lysis buffers of different pHs (**D**–**F**). 1.0% SDS was dissolved in lysis buffers of different pHs (**G**–**I**). 1.4% SDS was dissolved in lysis buffers of different pHs (**J**–**L**). *, *p* < 0.05; **, *p* < 0.01; ***, *p* < 0.001.

For developing a rapid diagnostic system, it was essential to optimize FICT conditions to prevent cross-reactivity of the bioconjugate on test lines (TL) that specifically bind with the target antigen on one viral particle. For this, all viral antigens need to be released, making the composition of the lysis buffer a critical factor. As the FICT assay lacked multiple washing steps, it readily showed non-specific reactions based on the antibody characteristics. We, therefore, tested different SDS concentrations at different pH values to determine an optimal range for a specific reaction. As displayed in Figure 3, the lysis buffer was tested with different concentrations of SDS (0.4%, 0.7%, 1.0%, and 1.4%) at different pH values (5, 9, and 11) for both Europium conjugates with 75 µL of sample-spiked H9N2 virus (0, 5, 10, and 40 HAU/mL). The best conditions of the lysis buffer for Europium A45-D5 FICT (0.1 M Tris, pH 9.0, 0.1 M EDTA, 1%SDS, and 0.5% Triton X-100) and Europium A39-G10 FICT (0.1 M Tris, pH 9.0, 0.1 M EDTA, 0.7% SDS, and 0.5% Triton X-100) among 12 different lysis buffers were selected for the FICT test. Raw data of lysis buffer optimization of FICT assays are presented in Figure S1.

### 2.4. FICT Assay Performance

For FICT, LOD was determined as previously described [24]. LOD is the lowest analytic concentration likely to be reliably distinguished from blank.

LOB = mean blank + 1.645 ∗ (SD blank)

LOD = LOB + 1.645 ∗ (SD lowest concentration sample)

**Figure 4:**
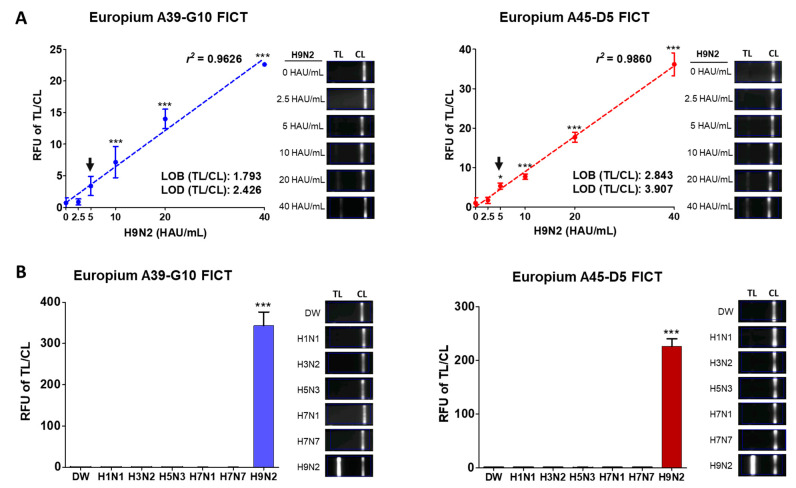
Performance of Europium A39-G10 FICT and Europium A45-D5 FICT assays. (**A**) Detection limit of the FICT assay for target influenza A virus H9N2. Two-fold serially diluted influenza A virus H9N2 was tested in Europium A39-G10 FICT and Europium A45-D5 FICT. A fluorescence image by Europium-FICT is shown in the left panel. The linear range for FICT using the two Europium nanoparticle—antibody conjugates were determined and the data (*n* = 3) are shown as the mean ± SD. (**B**) The specificity of Europium A39-G10 FICT and Europium A45-D5 FICT. The specificity of the optimized FICT was evaluated using influenza A virus H1N1, H3N2, H5N3, H7N1, H7N7, and H9N2 subtypes applied in high titer (1280 HAU/mL). *, *p* < 0.05; ***, *p* < 0.001.

Using A39-G10 McAb as a conjugate, the TL/CL of LOD was determined to be 5 HAU/mL for spiked H9N2 virus in distilled water (DW) (TL/CL: 3.39 ± 1.24) (Figure 4A). With A45-D5 McAb as a conjugate, the TL/CL of LOD was 3.91, indicating that the titer of LOD was 5 HAU/mL because TL/CL of 2.5 and 5 HAU/mL were 1.68 ± 0.65 and 5.3 ± 0.61 (Mean ± SD), respectively. Raw data for Europium A39-G10 FICT and Europium A45-D5 FICT LODs are shown in Figure S2. In the RDT, LOD was determined to be 40 HAU/mL by both Au NP-A39-G10 RDT and Au NP-A45-D5 RDT for spiked H9N2 virus in DW, based on the very faint band at the TL (Figure S3A). These values indicated that the Europium—FICT showed an 8-fold higher performance than the Au NP—RDT.

To confirm the specificity of both FICT assays, six types of influenza A virus (H1N1, H3N2, H5N3, H7N1, H7N7, H9N2) were tested by Europium A39-G10 FICT and Europium A45-D5 FICT (Figure 4B). Both assays did not show cross-reactivity with McAbs when other subtype viruses were present at 1280 HAU/mL. Raw data for Europium A39-G10 FICT and Europium A45-D5 FICT specificities are shown in Figure S4.

The FICT assay was optimized to detect the subtype H9 influenza A—specific virus in chicken fecal samples. After preparing a series of two-fold dilutions for the spiked H9N2 virus, the chicken feces swab sample was taken using a disposable swab and immersed in the lysis buffer. Subsequently, 200 µL of the sample was subjected to the FICT assay. The suitable lysis buffer volume for a fecal swab sample was optimized using different lysis buffer volumes and the most optimum lysis buffer volume (0.5 mL) was used for further fecal sample FICT assays (Figure S5).

**Figure 5:**
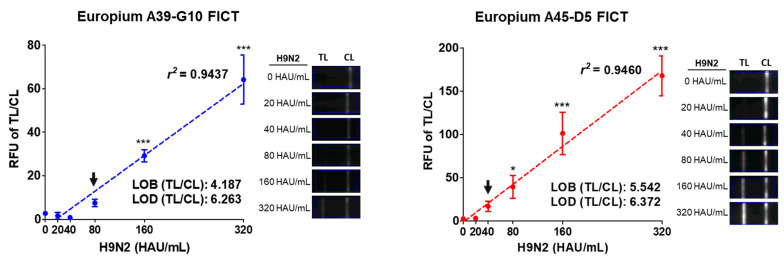
Comparison of Europium A39-G10 FICT and Europium A45-D5 FICT performance by detection limit of FICT assay for target influenza A virus H9N2 spiked in chicken feces. Two-fold serially diluted influenza A virus H9N2 subtype spiked in chicken feces was tested by Europium A39-G10 FICT and Europium A45-D5 FICT. The measured values were plotted and the fluorescence image by Europium-FICT is shown in the left panel. The linear range for FICT using the two Europium nanoparticle—antibody conjugates was determined and the data (*n* = 3) are shown as the mean ± SD. *, *p* < 0.05; ***, *p* < 0.001.

For FICT assays using fecal samples, the TL/CL of LOD was determined to be 80 HAU/mL (TL/CL: 7.49 ± 1.44) by Europium A39-G10 FICT and 40 HAU/mL (TL/CL: 17.01 ± 4.87) and by Europium A45-D5 FICT for the spiked H9N2 virus in chicken feces (Figure 5). In the RDT, LOD was calculated as 160 HAU/mL and 80 HAU/mL by Au NP A39-G10 RDT and Au NP A45-D5 RDT, respectively, based on the very faint band at the TL (Figure S3B). These values indicated that Europium A45-D5 FICT showed 2-fold higher performance than Europium A39-G10 FICT and the Europium FICT assay was 2-fold more sensitive to detect H9N2 virus spiked in chicken feces compare to Au NP RDT. Raw data of LODs for the spiked H9N2 virus in feces by Europium A39-G10 FICT and Europium A45-D5 FICT are shown in Figure S6.

Furthermore, the KD values of A39-G10 and A45-D5 were measured by surface plasmon resonance (SPR) (WOOJUNG BIO, Inc Company) to determine the affinity of both antibodies to the HA antigen of the H9 virus. The result showed that both A39-G10 (KD = 5.933 × 10^9^ M) and A45-D5 (KD = 6.746 × 10^9^ M) possessed strong affinity by KD value (Figure S7).

**Figure 6:**
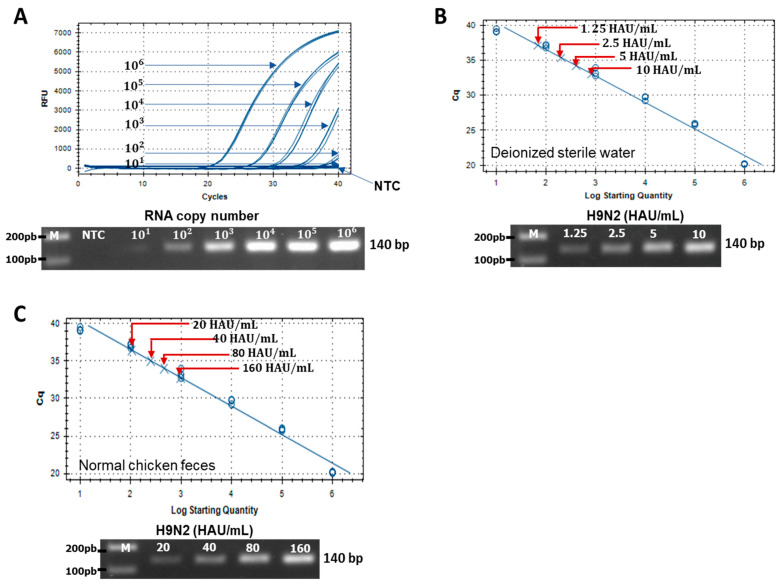
Assessment of FICT performance by rRT-PCR. (**A**) Standard curve was generated by the linear relationship between the threshold cycle (Ct) and RNA copy number of H9N2 hemagglutinin (HA). (**B**) Serially diluted H9N2 virus spiked in deionized sterile water (1.25–10 HAU/mL) and (**C**) serially diluted H9N2 virus (20–160 HAU/mL) spiked in normal chicken feces were subjected to RNA extraction and used for rRT-PCR.

The sensitivity of Europium FICT assays were compared with the standard rRT-PCR used for detecting H9N2 virus that was spiked in DW and chicken feces to determine RNA copy numbers at significantly different virus titers. The RNA copy number corresponded to a threshold cycle (Ct) value of 34.49 ± 0.314 (mean ± SD) and an RNA copy number/reaction mixture of 3.59 × 10^2^ ± 6.3 × 10^1^ (mean ± SD) for 5 HAU/mL of the spiked H9N2 virus in DW. For the spiked H9N2 virus in chicken feces at 40 HAU/mL and 80 HAU/mL Ct values of 34.13 ± 0.133 (mean ± SD) and 34.88 ± 0.123 (mean ± SD), respectively, and RNA copy numbers/reaction mixture of 2.79 × 10^2^ ± 2 × 10^1^ (mean ± SD) a Ct value of and 4.41 × 10^2^ ± 3.4 × 10^1^ (mean ± SD) were observed (Figure 6).

**Figure 7:**
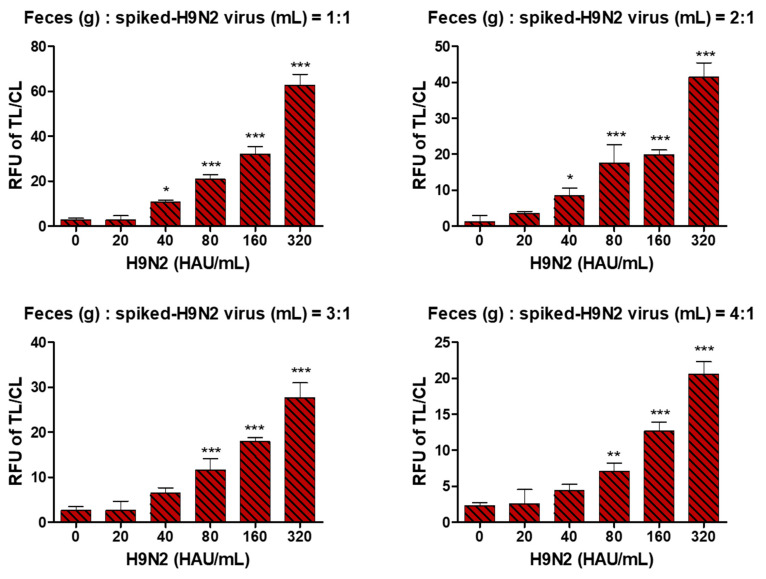
Determination of feces amounts to maintain FICT performance. Ratios of feces (g) to spiked H9N2 virus (mL) (1:1; 2:1; 3:1; 4:1) were tested. Two-fold serially diluted influenza A virus H9N2 subtype was tested by Europium FICT. *, *p* < 0.05; **, *p* < 0.01; ***, *p* < 0.001.

A series of two-fold dilutions of the H9N2 virus were spiked in fecal samples in various ratios to determine FICT performance affected by the chicken feces amount. After immersing the fecal sample swab into the lysis buffer, 200 µL of the sample was used for the FICT assay. The ratio of feces and spiked virus solution (3:1) was maintained with a FICT LOD of 40 HAU/mL (Figure 7). Raw data of FICT are presented in Figure S8. When the FICT specificity for maximum fecal amounts was determined using six types of influenza A virus (H1N1, H3N2, H5N3, H7N1, H7N7, H9N2) at 1280 HAU/mL, no cross-reactivity of H9N2 with other subtype viruses was observed by the FICT assay (Figure S9).

**Figure 8:**
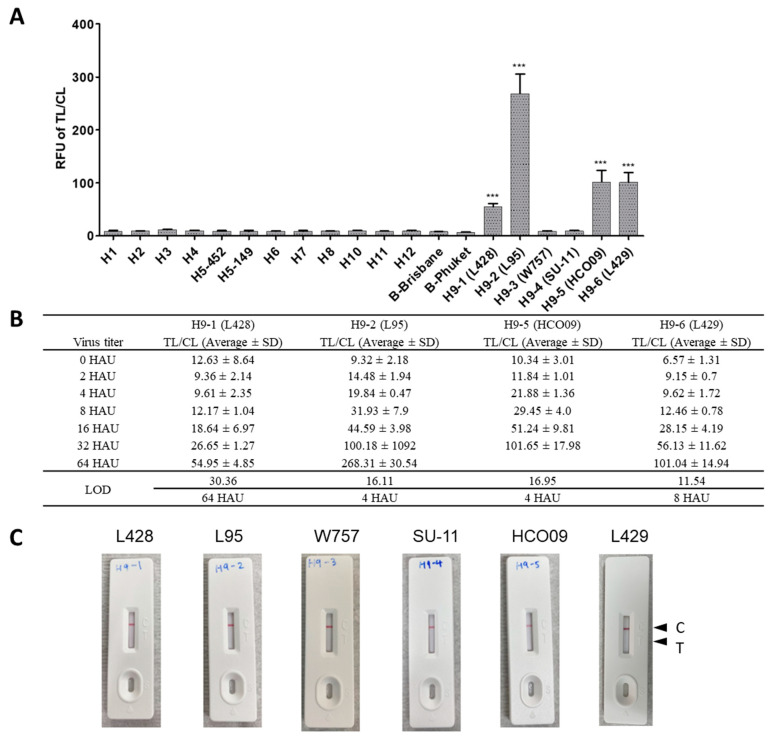
Specificity of the FICT assay. (**A**) The specificity of Europium A45-D5 FICT was evaluated by using Influenza A/B subtype viruses in high titer (64 HAU/25 μL). (**B**) Among H9 subtype viruses tested, FICT-positive H9 strains were further tested for limit of detection by serial dilution of each virus (B). (**C**) A commercial Abbexa Avian Influenza H9 Virus Antigen Rapid Test Kit (RDT) was tested with five H9 subtype strains at 64 HAU/25 μL. Virus information used is listed in Table S2. C, control line; T, test line. ***, *p* < 0.001.

Another research group at Chungbuk National University also evaluated the FICT assay to verify its performance. The specificity of the FICT assay was assessed using six H9 virus strains, 12 other influenza A subtypes and two influenza type B strains (B-Brisbane, B-Phuket) (Figure 8A). The raw FICT data are displayed in Figure S10. The results confirmed that the FICT assay did not show cross-reactivity with other virus subtypes. The FICT assay could detect four of six H9 virus strains (H9N2 (L428); H9N2 (L95); H9N2 (HCO09); H9N2 (L429)). The assay was performed in triplicate using virus titers ranging from 1 to 64 HAU to evaluate the LOD of the FICT assay to detect four strains of the H9N2 virus. The raw data of results for FICT are shown in Figure S11. The TL/CL of LOD was determined to be 64 HAU, 4 HAU, 4 HAU, and 8 HAU for H9N2 (L428), H9N2 (L95), H9N2 (HCO09), and H9N2 (L429), respectively. Compared with the commercial RDT, all six H9 subtypes were not detected, with no signal band at the test line (Figure 8B). These results indicated that compared to the commercially available RDT which did not recognize six H9 strains at all, our FICT has a superior capability of detecting H9 viruses (Figure 8B).

Finally, clinical studies were carried out independently by Chungbuk National University and Wonkwang University research groups using A/Chicken/Korea/LPM429/2016 (H9N2) strain and A/Chicken/Korea/KNUSWR09/2009 (H9N2) strain, respectively.

### 2.5. Clinical Study

To demonstrate that FICT assays could detect the virus in the samples, 4–5-week-old SPF chickens were infected with A/Chicken/Korea/LPM429/2016 (H9N2) (H9N2_L429) (*n* = 6), A/chicken/Korea/KNUSWR09/2009 (H9N2) (H9N2_KNUSWR09) (*n* = 3), or PBS as the negative control (*n* = 3). Cloacal swab samples and fecal samples were collected on days 2-, 4-, and 6 post-infection and tested in parallel by rRT-PCR, FICT, and RDT.

For the rRT-PCR analysis, samples targeting the H9-HA gene with Ct values ≤ 40 were considered positive. Among the collected cloacal swabs, positive Ct values were observed at 4 dpi (3/6) to 6 dpi (3/6) of H9N2_L429 strain, and at 2 dpi (1/3) to 6 dpi (2/3) of H9N2_KNUSWR09 strain. In contrast, in fecal samples collected as early as 2 dpi (37.5% and 33.33% of H9N2_L429 strain and H9N2_KNUSWR09 strain, respectively) to 6 dpi (100%) Ct values implied a higher rate of detection of the H9N2 virus from fecal samples than cloacal swabs. All values for specimens obtained using rRT-PCR and FICT of H9N2_L429 and H9N2_KNUSWR09 strains are summarized in Tables S3 and S4, respectively. As shown in Tables S3 and S4, none of the negative specimens showed Ct values when analyzed using rRT-PCR with H9 HA primers and probe.

**Figure 9:**
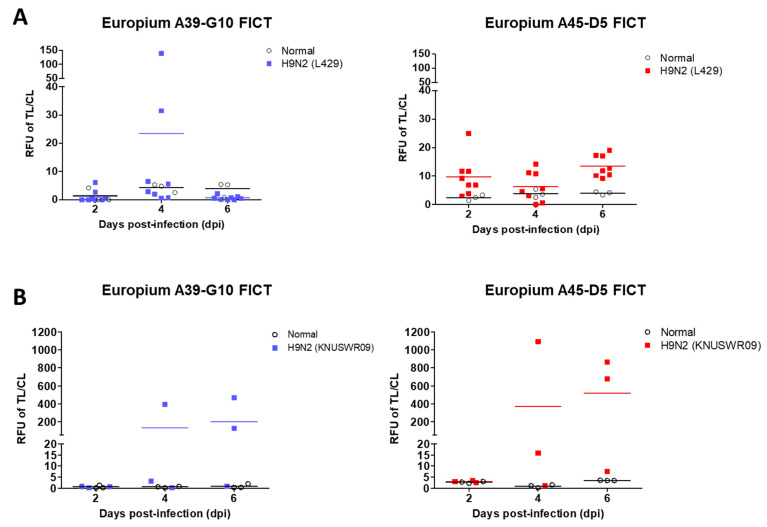
Analysis of Europium A45-D5 FICT and Europium A39-G10 FICT using fecal samples from SPF chickens on different days post-inoculation with two H9N2 strains. The cut-off value used for detection of the H9N2 virus was applied to determine the presence of the virus in fecal samples of (**A**) A/Chicken/Korea/LPM429/2016 (H9N2) strain, (**B**) A/chicken/Korea/KNUSWR09/2009(H9N2) strain.

For both FICT assays, cloacal samples showed lower sensitivity than the fecal samples compared to the rRT-PCR performance with 50% (3/6) and 66.67% sensitivity (2/3) of H9N2_L429 strain and H9N2_KNUSWR09 strain, respectively, at both 4 dpi and 6 dpi (Table 1, Figure S12). The Europium A39-G10 FICT assay in fecal samples yielded positive TL/CL values up to 4 dpi (37.5%) and 6 dpi (66.67%) in H9N2_L429 and H9N2_KNUSWR09 strains, respectively. In contrast, the Europium 45-D5 FICT assay showed positive TL/CL values of 2 to 6 dpi with 100% sensitivity using H9N2 strains (Table 1, Figure 9). Europium A45-D5 FICT was more effective in detecting the virus in fecal samples than Europium A39-G10 FICT and, therefore, showed higher sensitivity. Raw data obtained from the FICT analyses of clinical specimens of H9N2_L429 and H9N2_KNUSWR09 strains are presented in Figures S13 and S14, respectively. Further, the RDT kit using the same McAb pairs could detect 25% and 66.67% of fecal samples of the H9N2_L429 strain at 4 dpi and H9N2_KNUSWR09 strain at 6 dpi, respectively. The RDT screening with cloacal and fecal samples showed lower sensitivity than FICT (Table 1). The raw data of RDT results are shown in Figures S15 and S16. The results indicated that FICT was more sensitive for virus detection in fecal samples than the colloidal gold-based rapid diagnostic test.

Europium has been used in dry conditions of FICT in several previous reports, but the duration of the stability of Europium FICT was rarely reported. In this study, the Europium-McAb conjugate was dried at 25 °C to evaluate its stability. Significantly, Europium-McAb conjugate maintained LOD of spiked-H9N2 virus in fecal samples up to 21 days (Figure S17). The performance of Europium-McAb FICT at 7, 14, and 21 days in dry conditions was lower than the fresh conjugate and decreased over time. However, linear regression was still excellent in this range of virus even after 21 days, implying that Europium conjugates should be further optimized for more extended usage in dry conditions.

## 3. Discussion

The LPAI viruses like H9N2 circulate in multiple avian species resulting in significant economic losses due to low, moderate, or high mortality with apparent clinical signs characterized by depression, edema of the head, cyanosis of the comb and legs, and a drop in egg production [2,7,8,9,10,11,12]. Even though H9N2 avian influenza viruses circulate worldwide, only 59 human infection cases were reported between 1997 and July 2020.

H9N2 generally shows low pathogenicity and receptor-binding preference to avian species but some strains possess human-like receptor specificity [15], helping their transmission to mammalian species, including humans. In addition, the AIV genome with eight segments enabled the re-assortment of H9N2 viruses with other concurrently circulating AIVs contributing to the emergence of highly pathogenic avian influenza (HPAI) viruses like H5N1, H7N9, and H10N8 [31,32,33]. Therefore, given the future pandemic influenza potential of H9N2 viruses, developing a high-performance diagnostic system at the clinical level for primary screening of these viruses is essential.

Several diagnostic systems for detecting the H9N2 influenza virus have been developed, and these methods have shown ultrasensitive performance in detecting antibodies against H9N2 or the H9 viral antigen with 98.9% sensitivity and 98.1% specificity [26,34]; however, none qualified as a rapid diagnostic system.

A fluorescent material was applied in a previous study to develop the H9-specific diagnostic system with high sensitivity with LOD of 0.25 HAU of H9 virus [27]. The TL value was used without accurate normalization of the CL, resulting in qualitative rather than quantitative detection of targets. Moreover, the study did not perform clinical validation with animal models.

In our previous study, we developed a smartphone-based rapid fluorescent diagnostic system for detecting H9N2 virus in specific-pathogen-free chickens with a relative sensitivity of 94.44% and 95.23% in oropharyngeal and cloacal specimens, respectively [24]. However, the target detection was the internal nucleoprotein (NP) that did not provide the subtype information in our previous study.

For rapid diagnosis, a fecal examination would be more convenient as a primary screening method to diagnose infection in animal groups than using direct contact specimens of animal bodies, such as oropharyngeal and cloacal samples. However, the mass of feces is an obstacle for primary screening due to the highly aggrading substances in feces; thus, it is scarce of feces specimen applicable diagnostic system in the field.

In the current study, a rapid system for detecting the H9 virus-specific HA antigen was developed using two different antibody pairs with different epitope recognition (A27-9(capture)/A45-D5 (detect): one was for the linear epitope (A27-9(capture)/A39-G10(detect) and the other was a conformational epitope as a detection antibody in the pair, and the rapid fluorescent diagnostic assay was optimized in feces. Interestingly, although between two pairs (A27-9/A39-G10; A27-9/A45-D5), A27-9/A39-G10 yielded a higher fluorescence intensity than A27-9/A45-D5 (Figure 2B,D), feces data indicated that the A27-9/A45-D5 pair had the stable LOD (Figure 5). It seems that the antibody function of A27-9/A39-G10, but not of A27-9/A45-D5, was damaged in the presence of feces because a faint fluorescent intensity was observed. It is an interesting finding that OD or fluorescence intensity might not be the absolute parameters to develop a specific antibody for fecal samples.

As the KD value of A45-D5 corresponded to our observation of FICT in feces, KD could be a useful parameter. However, A39-G10 that is moderate FICT performance in feces, possessed still strong affinity in KD value, therefore, KD value could be considered together with epitope status to determine the efficient antibody applicable in fecal samples.

The varying performance of antibodies depends on the binding sites or epitopes they recognize [35]. Epitopes are generally divided in two categories, linear epitopes, where a stretch of continuously divided amino acids is sufficient for binding, and conformational epitopes, where key amino acid residues are brought together by protein folding [36]. Conformational epitope antibodies might be preferred for assays involving protein targets in their native state. In contrast, linear epitope antibodies might be preferred for applications in which the target antigen is wholly or partially denatured during the sample preparation step [35]. To improve FICT assay performance, we applied linear epitope antibodies (A45-D5) and conformational epitope antibodies (A39-G10) in FICT assays and evaluated their performance.

In the current study, we developed a rapid diagnostic system using Europium nanoparticles conjugated with H9 specific McAbs to detect the virus in fecal specimens. Before applying on the strip, fecal samples were treated with lysis buffer to expose target proteins, enabling McAbs used in the FICT assay to recognize their target. Lysis buffer used for fecal sample testing contained a high concentration of SDS to remove non-specific binding (Figure 3) caused by denatured target proteins on the surface of the virus. The Europium A45-D5 and Europium A39-G10 FICT assays had the same LODs of spiked virus (5 HAU/mL) as well as the KD value (the equilibrium dissociation constant between the antibody and its antigen) (Figure S7). However, interestingly, the clinical performance of the A39-G10 FICT assay (sensitivity = 37.5% to 66.67%) was relatively lower than the Europium A45-D5 FICT assay (sensitivity = 100%), although LODs for both systems were not very different with the spiked virus in DW and in feces, indicating that A39-G10 FICT assay has a lower efficiency in clinical applications.

We could not test all commercially available H9N2-specific diagnostic kits and used only the commercial RDT (Avian Influenza H9 Virus Antigen Rapid Test Kit—Abbexa, Cambridge, United Kingdom UK) for comparison with our assay. As it did not detect any H9N2 strains used in our study, we could not provide the relative performance between the commercial RDT and our assay. The negative reaction observed with the commercial kit might be because the RDT kit was designed for detecting viruses of other lineages that have significant variation with the Korean lineage, indicating that our assay had improved performance to recognize Korean lineage H9N2 viruses [Figure 8].

rRT-PCR is one of the most widely used methods for detecting viral genes because of its high sensitivity and specificity. Chan et al. reported the presence of the influenza A virus in stool specimens with viral loads ranging between 4.9 × 10^3^ to 8.0 × 10^7^ copies per g of stool [37]. In this study, the rRT-PCR of 2.79 × 10^2^ ± 2 × 10^1^ and 4.41 × 10^2^ ± 3.4 × 10^1^ cDNA copies corresponded to the median LOD of Europium A45-D5 FICT and Europium A39-G10 assays, respectively, for H9 HA in fecal specimens. Taken together, we believe that the LOD and quantitative range enabled the detection of influenza A virus in stool specimens.

Avian feces samples are mostly acidic, with pH ranging between 4.5 to 6.5 [38]. To optimize FICT in stool samples, SDS concentration and pH conditions of the lysis buffer were adjusted as these factors are critical for the lysis of massive aggregates to release viral antigens [39] and help neutralize feces pH suitable for Europium-antibody conjugation with the antigen. The LODs of FICTs were kept stable when high amounts of fecal samples were used (Figure 7).

In summary, to the best of our knowledge, our study is the first to clinically evaluate a specific and rapid kit with the ability to detect the influenza H9 subtype in fecal samples. We believe that the H9-specific FICT assay developed in the present study can be used in poultry surveillance and wild birds AIV case management.

## 4. Materials and Methods

### 4.1. Reagents

Rabbit anti-mouse IgG H&L (horseradish peroxidase (HRP)) and goat anti-mouse IgG H&L (FICT) ab6758 were bought from Abcam (Cambridge, UK). Europium nanoparticles (200 nm diameter) were purchased from Bangs Laboratories Inc. (Fishers, IN, USA). Colloidal gold (40 nm diameter) was obtained from Bore Da Biotech Co., (Gyeonggi, Republic of Korea). N-(3-Dimethylaminopropyl)-N′-ethylcarbodiimide hydrochloride (EDC) and N-hydroxysulfosuccinimide sodium salt (Sulfo-NHS) were acquired from Thermo Scientific (Waltham, MA, USA). All other chemicals were purchased from Sigma-Aldrich (St. Louis, MO, USA) and used without further purification. Anti-influenza A nucleoprotein (NP) (Clone 3D3) was provided by Professor Ho-Joon Shin, Ajou University, Suwon, Republic of Korea.

### 4.2. Cells and Viruses

Madin–Darby Canine kidney (MDCK, NBL-2, ATCC^®^ CCL-34TM) cells were purchased from American Type Culture Collection (ATCC, Manassas, VA, USA). H1N1 (A/California/07/09A/H1N1) was obtained from the Korea Center for Disease Control and Prevention. H3N2 was purchased from Korean National Research Resource Center (Seoul, Korea). AI virus (H5N3 (A/spot-billed duck/Korea/KNU SYG06/2006), H7N1 (A/common teal/Korea/KNU YSR12/2012), H7N7 (A/mallard/Korea/KNU GPH12/2011) and H9N2 (A/chicken/Korea/KNUSWR09/2009)) were kindly provided by Professor Haan Woo Sung, Kangwon National University, Gangwon, the Republic of Korea (Table S1). The egg-cultured virus stock was titrated by the HA assay performed, as previously described [40]. Other viruses from Chungbuk National University are listed in Table S2.

### 4.3. Cell Fusion and Hybridoma Cell Cloning

H9N2 (A/chicken/Korea/KNUSWR09/2009 (H9N2)) virus was incubated for 48 h at 37 °C in 0.2% final concentration of formalin in phosphate-buffered saline 1X (PBS 1X). After inactivation, formalin was removed by dialysis against PBS 1X, and the dialyzed samples were stored at −80 °C until analysis. The virus was mixed with an equal volume of Freund’s complete adjuvant (Sigma-Aldrich, St. Louis, MO, USA) and injected intraperitoneally into six-week-old female BALB/c mice obtained from Orient (Seongnam, Gyeonggi, Republic of Korea). Boosts consisted of the inactivated H9N2 virus (200 HAU/100 μL) mixed with an equal volume of Freund’s incomplete adjuvant biweekly.

We used enzyme-linked immunosorbent assay to confirm the antibody titer in mouse serum and produced cell lines that secrete McAbs using a cell-fusion technique described previously [41]. Splenocytes were extracted from a selected immune mouse and fused with myeloma cells (F/0 cell line) at a ratio of 1:5 to 1:10 in 50% polyethylene glycol solution (Sigma-Aldrich, St. Louis, MO, USA) and seeded in each well of a 96-well culture plate. Hybridoma cells were selected by subculturing in HAT (hypoxanthine, aminopterin, and thymidine) and HT (hypoxanthine and thymidine) media (Fishers, IN, USA) in a 5% CO_2_ incubator at 37 °C (Sanyo, Osaka, Japan) for 2 weeks. When colonies appeared in each well, the hybridoma cell culture supernatant was screened by ELISA. After sub-cloning with limiting dilutions, samples from selected hybridoma colonies were transferred to 75-cm^2^ tissue culture flasks. For scaled-up McAb production, McAb-producing cells were intraperitoneally injected into 10-week-old female BALB/c mice. Two weeks later, mouse ascites was collected and centrifuged at 5000× *g* for 15 min. The purified McAbs from the ascites were obtained using a protein A agarose column (Amersham Biosciences, Uppsala, Sweden) and identified by Western blotting.

### 4.4. Enzyme-Linked Immunosorbent Assay (ELISA)

Indirect ELISA was performed as described previously [23]. Briefly, the viruses were diluted with 50 mM bicarbonate/carbonate coating buffer (pH 9.6) at 1000 HAU/mL (Hemagglutination unit), coated on a 96-well microtiter plate (Greiner, Germany), and incubated at 37 °C for 2 h. The plate was washed with 200 μL PBS, 0.1% Tween 20 (PBS-T, pH 7.4), and then blocked with 5% non-fat dry milk at 37 °C for 2 h. To react the antibody, first McAbs (2 μg/100 μL/well) were added to each well and positive control (anti-influenza A NP, 2 μg/100 μL/well) following which the subtype virus was added to each well and incubated at 37 °C. After 1 h, horseradish peroxidase (HRP)-conjugated rabbit anti-mouse IgG (Abcam, Cambridge, UK) was added to each well according to the manufacture’s protocol. Stringent washing with PBS-T was performed five times to remove nonspecific binding, and 100 μL of 3,3′,5,5′-tetramethyl benzidine (Sigma-Aldrich, St. Louis, MO, USA) substrate solution was added.

### 4.5. Western Blot Analysis

The six subtype viruses (H1N1, H3N2, H5N3, H7N1, H7N7, and H9N2) and the uninfected control (Allantoic fluid of normal chicken egg) were subjected to 12% SDS-PAGE, and the gel was electrophoresed at 100 V for 2 h. The gel was soaked in the transfer buffer, and the resolved proteins were transferred to a polyvinylidene difluoride membrane. The membrane was blocked with 5% non-fat milk for 2 h at 37 °C and each antibody diluted to a concentration of 10 μg/mL was added for 1 h at 37 °C. The HRP-conjugated antibody was used according to the manufacturer’s protocol. Anti-influenza NP (Clone 3D3) was used for loading control of each virus. Finally, Clarity Western ECL substrate (Bio-Rad, Hercules, CA, USA) was used to visualize the band using the ChemiDoc MP System (Bio-Rad, Hercules, CA, USA).

### 4.6. Sandwich Fluorescent-Linked Immunosorbent Assay (FLISA)

A black 96-well microtiter plate (SPL, Gyeonggi, Republic of Korea) was coated with 100 µL/well of H9 subtype-specific monoclonal antibodies (McAbs) (10 µg/mL) and incubated at 4 °C for overnight. Subsequently, each well was washed with PBS, 0.1% Tween 20 (PBS-T) and blocked with 200 µL/well of 5% Bovine Serum Albumin (BSA, Sigma-Aldrich, St. Louis, MO, USA) and 10 mM Glycine (Sigma-Aldrich, St. Louis, MO, USA) in PBS-T for 2 h at 37 °C. Virus samples were diluted with coating buffer (50 mM bicarbonate/carbonate (pH 9.6)) to 1000 HAU/mL, 100 µL were loaded in each well, and incubated for 1 h at 37 °C. After washing 3 times with PBS-T, 100 µL of Europium-H9 subtype-specific monoclonal antibody conjugate diluted 20-folds with blocking buffer were added and incubated at 37 °C for 1 h. The plates were washed five times with PBS-T, 100 µL PBS was added and fluorescence (355 nm excitation, 612 nm emission) was measured with an Infinite F200 microplate reader (TECAN, Männedorf, Switzerland).

### 4.7. Immunofluorescence Assay (IFA)

IFA was performed as described previously [41,42]. MDCK cell monolayers were grown on glass coverslips placed in 6-well plates and infected with H1N1 virus, H9N2 virus at MOI 0.01 in DMEM containing 1% antibiotic, 1 µg/mL N-tosyl-L-phenylalanine chloromethyl ketone (TPCK-treated trypsin, Sigma-Aldrich, St. Louis, MO, USA). After 24 h, cells were washed with PBS and fixed with 4% paraformaldehyde in PBS (pH 6.9) for 10 min at room temperature. Subsequently, coverslips were washed three times with 0.1% Tween 20 in PBS, and 0.2% Triton X-100 was added to each well for permeabilization. Cells were blocked with 5% BSA and 10 mM Glycine in PBS-T at room temperature for 2 h. After washing 3 times with PBS-T, coverslips were incubated with the first monoclonal antibody anti Influenza A virus H9N2 subtype hemagglutinin 1:100 dilutions for 1 h at RT and incubated with the secondary antibody goat anti-mouse IgG H&L (FICT) ab6758 (Abcam) for 1 h. Finally, coverslips were dried and mounted with a mounting medium containing 4′,6-diaminodo-2-phenylindole (DAPI) (Vector lab, Burlingame, California, USA). Images were acquired using a fluorescence microscope (Olympus, Tokyo, Japan) at 400× magnification.

### 4.8. Conjugation of Europium Nanoparticles

Antibody was covalently conjugated to Europium by a well-established procedure from Bangs Laboratories. Then, 20 µL Europium (0.2 µm, 1% w/t) was added to 980 µL 0.05 M MES (pH 6.1) and incubated for 1 h at 25 °C in the presence of 26 µL 5 mM EDC (N-(3-Dimethylaminopropyl)-N’-ethylcarbodiimide hydrochloride) and 200 µL 50 mM sulfo-NHS (N-hydroxysulfosuccinimide sodium salt). Subsequently, sulfo-NHS was removed by centrifugation at 27,237× *g* for 5 min. The activated Europium was mixed with 60 µg of 1 mg/mL antibody in 1000 µL 0.1 M sodium phosphate (pH 8.0) and allowed to react for 2 h at 25 °C. After centrifugation at 27,237× *g* for 5 min, the Europium-conjugated antibody was collected and re-suspended in 400 µL 2 mM borax (pH 8.0) including 0.1% BSA and stored at 4 °C.

### 4.9. Lateral Flow Test Strips for FICT

The test strips used consisted of four components: sample application pad, conjugate pad, nitrocellulose (NC) membrane, and absorbent pad. The test line (TL) of the strip was prepared by dispensing the desired volume of 2.0 mg/mL mouse monoclonal (anti-influenza H9 subtype-specific McAbs) and 0.5 mg/mL polyclonal goat anti-mouse IgG was used on the control line (CL). The diagnostic strip was tested after drying the membrane at 30 °C for 2 days. To perform the FICT assay, 2 µL of Europium-conjugated H9 subtype-specific McAbs was applied onto the conjugate pad, and a mixture of 75 µL of samples with 75 µL of lysis buffer was added onto the sample pad for 20 m. The results of test strips were read with a portable fluorescent strip reader at excitation and emission wavelengths of 355 nm and 612 nm, respectively (Medisensor, Daegu, Republic of Korea). Both TL and CL signals were measured and the TL/CL ratio was calculated.

### 4.10. Real-Time Reverse Transcription-Polymerase Chain Reaction (rRT-PCR)

rRT-PCR was performed using a Quantitect Probe RT-PCR Kit (QIAGEN, Hilden, Germany) to determine the cycle threshold (Ct) values using a CFX96 Real-Time PCR Detection System (Bio-Rad, Hercules, CA, USA). The H9 primers, probes, and RT-PCR conditions were described previously (https://www.who.int/influenza/gisrs_laboratory/molecular_diagnosis/en/ (accessed on 16 January 2020)). For standard RNA copy number, the template was generated in plasmid pGEM-T Easy (Promega, Madison, WI, USA), including a 140-base pair (bp) HA1 insert. In vitro transcription of HA1 RNA used a RiboMax (Promega, Madison, WI, USA) kit to determine the RNA copy number for the limit of detection (LOD) of FICT. The standard curve was calculated automatically by plotting the Ct values against each standard of known RNA copy number and extrapolating the linear regression line of this curve. The PCR products were analyzed in agarose gels (2%).

### 4.11. Rapid Detection Test Assay (RDT)

Antibody was absorptive conjugated to colloidal gold (Au NP), and 3 mL (0.04 µm, OD (525) = 1.043) was centrifuged at 27,237× *g* for 5 min to collect the colloidal gold pellet. Subsequently, 950 µL, 20 mM Tris buffer (pH 9.5), and 50 µL of 1 mg/mL antibody were added and incubated for 3 h at 25 °C. After centrifugation at 27,237× *g* for 5 min, the Au NP-McAb conjugate was collected, washed with 1000 µL 2 mM borax (pH 9.0), re-suspended in 100 µL storage buffer (0.1% BSA in 2 mM borax (pH 9.0)), and stored at 4 °C. Further, the FICT assay performance was compared with the commercial Abbexa (Cambridge, UK) Avian Influenza H9 Virus Antigen Rapid Test Kit (RDT) (Cat. No.: abx092107) with an equal ratio of virus following the manufacturer’s instructions.

### 4.12. Clinical Study

For the clinical study with A/Chicken/Korea/LPM429/2016 (H9N2) strain at Chungbuk National University, 4–5-week-old specific-pathogen-free (SPF) white leg-horn chickens (G. gallus domesticus, Namdeok Senitek, Republic of Korea) (*n* = 6) were inoculated with 500 µL of 10^7^ EID_50_/mL of A/LPM/428 (H9N2) via oropharyngeal route. As a negative control, three white leg-horn chickens of the same age were inoculated with 500 µL PBS and kept in a separate cage. Cloacal swab samples (3 swabs/bird) were collected from 2-, 4-, and 6-days post-infection (dpi) from each bird. Additionally, during the sample collection, stool samples (*n* = 8/cage) were randomly collected from each birdcage.

For A/Chicken/Korea/KNUSWR09/2009 (H9N2) strain, 4-week-old white-leghorn chickens (*n* = 3) in three separate cages were inoculated with 100 µL of 10^5^ EID_50_/mL of A/chicken/Korea/KNUSWR09/2009 (H9N2) via oropharyngeal route and three white leg-horn chickens of the same age were inoculated with PBS as a negative control. Cloacal swab samples and fecal samples at 2-, 4-, and 6-days post-infection were collected from each bird. All collected samples were processed and measured using FICT/RDT.

### 4.13. Statistics

All data were shown as mean ± standard deviations (SD) of biological replicates and plotted using GraphPad Prism 5.0 (GraphPad, La Jolla, CA, USA) [43].

## Figures and Tables

**Table 1:** Comparison of the clinical diagnostic performance of Europium A45-D5 FICT assay with Europium A39-G10 FICT and the conventional RDT.

Specimen ^a^	Sensitivity (%)				
	2 dpi				
	RT-PCR	FICT		RDT	
		D5	G10	D5	G10
Cloacal	0 (0/6)	0 (0/6)	0 (0/6)	0 (0/6)	0 (0/6)
Feces	37.50 (3/8)	75.00 (6/8)	12.50 (1/8)	12.50 (1/8)	12.50 (1/8)
	4 dpi				
	RT-PCR	FICT		RDT	
		D5	G10	D5	G10
Cloacal	50.00 (3/6)	0 (0/6)	0 (0/6)	0 (0/6)	0 (0/6)
Feces	75.00 (6/8)	37.50 (3/8)	37.50 (3/8)	25.00 (2/8)	12.50 (1/8)
	6 dpi				
	RT-PCR	FICT		RDT	
		D5	G10	D5	G10
Cloacal	50.00 (3/6)	0 (0/6)	0 (0/6)	0 (0/6)	0 (0/6)
Feces	100 (8/8)	100 (8/8)	0 (0/8)	0 (0/8)	0 (0/8)
**Specimen ^b^**	**Sensitivity (%)**				
	2 dpi				
	RT-PCR	FICT		RDT	
		D5	G10	D5	G10
Cloacal	33.33 (1/3)	0 (0/3)	0 (0/3)	0 (0/3)	0 (0/3)
Feces	33.33 (1/3)	0 (0/3)	0 (0/3)	0 (0/3)	0 (0/3)
	4 dpi				
	RT-PCR	FICT		RDT	
		D5	G10	D5	G10
Cloacal	66.67 (2/3)	33.33 (1/3)	0 (0/3)	0 (0/3)	0 (0/3)
Feces	100 (3/3)	66.67 (2/3)	33.33 (1/3)	33.33 (1/3)	33.33 (1/3)
	6 dpi				
	RT-PCR	FICT		RDT	
		D5	G10	D5	G10
Cloacal	66.67 (2/3)	66.67 (2/3)	0 (0/3)	33.33 (1/3)	0 (0/3)
Feces	100 (3/3)	100 (3/3)	66.67 (2/3)	66.67 (2/3)	66.67 (2/3)

^a^ A/Chicken/Korea/LPM429/2016 (H9N2) strain; ^b^ A/Chicken/Korea/KNUSWR09/2009(H9N2) strain.

## Data Availability

The data presented in this study are available in Supplementary Materials.

## Supplementary Materials

The following are available online at https://www.mdpi.com/article/10.3390/ijms22168823/s1.
