# Supplementary material for: Development of a Rapid Fluorescent Diagnostic System to Detect Subtype H9 Influenza A Virus in Chicken Feces
Source: Int J Mol Sci. 2021 Aug 17;22(16):8823. doi: 10.3390/ijms22168823 (PMC8396311; doi:10.3390/ijms22168823)
Supplement: Supplementary file 1 [file ijms-22-08823-s001.zip › ijms-1307235-supplementary.pdf]

Supplementary Information

Supplementary tables

Table.S1. List of virus in Wonkwang University

Table.S2. List of virus in Chungbuk University

Table.S3. Real time RT PCR (rRT PCR) with clinical specimens (A/Chicken/Korea/LPM429/2016 (H9N2) strain).

Table.S4. Real time RT PCR (rRT PCR) with clinical specimens (A/chicken/Korea/KNUSWR09/2009(H9N2) strain).

Supplementary figures

Fig.S1. Raw data of lysis buffer optimization.

Fig.S2. Raw data of limit of detection for spiked-H9N2 virus in distilled water (DW).

Fig.S3. Limit of detection of conventional rapid diagnostic test (RDT).

Fig.S4. Raw data of specificity for spiked-H9N2 virus in DW.

Fig.S5: Optimization amount of lysis buffer for feces condition.

Fig.S6. Raw data of limit of detection for spiked-H9N2 virus in feces.

Fig.S7. Measurement of KD values for two antibodies (A39-G10 and A45-D5).

Fig.S8. Raw data of limit of feces amount to keep FICT performance.

Fig.S9. The specificity of FICT assay with maximum amount of feces condition.

Fig.S10. Raw data of specificity of FICT for influenza A/B virus in Chungbuk.

Fig.S11. The limit of detection of four H9N2 virus strains in Chungbuk.

Fig.S12. Analysis of Europium A45-D5 FICT and Europium A39-G10 FICT using cloacal samples from SPF chickens at different day post inoculation with two H9N2 strains.

Fig.S13. Raw data of Europium A45-D5-FICT and Europium A39-G10 FICT with clinical specimens from SPF chickens at different day post-infection with A/Chicken/Korea/LPM429/2016 (H9N2) strain.

Fig.S14. Raw data of Europium A45-D5-FICT and Europium A39-G10 FICT with clinical specimens from SPF chickens at different day post-infection with A/chicken/Korea/KNUSWR09/2009(H9N2) strain.

Fig.S15. RDT with clinical specimens from SPF chickens at different day post-infection with A/Chicken/Korea/LPM429/2016 (H9N2) strain.

Fig.S16. RDT with clinical specimens from SPF chickens at different day post-infection with A/chicken/Korea/KNUSWR09/2009(H9N2) strain.

Fig.S17. Duration of FICT in dry condition.

Table.S1. List of virus in Wonkwang University

Avian influenza viruses in Wonkwang University		
1	H1N1	A/H1N1/2009/CA
2	H3N2	Influenza A/H3N2 isolated from humans in 2014 was donated from the Korean National Research Resource Center (Registration number: KBPV-VR-85)
3	H5N3	A/spot-billed duck/Korea/KNU SYG06/2006(H5N3)
4	H7N1	A/common teal/Korea/KNU YSR12/2012(H7N1)
5	H7N7	A/mallard/Korea/KNU GPH12/2011(H7N7)
6	H9N2	A/chicken/Korea/KNUSWR09/2009(H9N2)

Table.S2. List of virus in Chungbuk University

Avian influenza viruses in Chungbuk University		
1	H1	A/California/04/2009 (H1N1)
2	H2	A/Em/Korea/ W357/2008 (H2N3)
3	H3	A/Perth/16/2009 (H3N2)
4	H4	A/aquatic bird/Korea/W137/2006 (H4N7)
5	H5	W149 (H5N1) A/EM/Korea/W149/2006(H5N1)
6	H5	W452 (H5N8) A/MD/Korea/W452/2014(H5N8)
7	H6	A/Em/Korea/W502/2015 (H6N2)
8	H7	W557 (H7N7) A/duck/Korea/557/2016 (H7N7)
9	H8	A/Em/Korea/W563/ 2016 (H8N6)
10	H9-L428	A/Chicken/Korea/LPM428/2016 (H9N2)
12	H9-L95	A/Chicken/Korea/LPM95/2006 (H9N2)
13	H9-W757	A/Em/Korea/W757/2019 (H9N2)

14	H9-SU-11	A/Em/Korea/Su-11/2019 (H9N2)
15	H9-HCO09	A/chicken/Korea/HC09/2009 (H9N2)
16	H9-L429	A/Chicken/Korea/LPM429/2016 (H9N2)
17	H10	A/aquatic bird/Korea/w296/2007 (H10N4)
18	H11	A/Em/Korea/W552/2016 (H11N9)
19	H12	A/Em/Korea/W373/2008 (H12N5)
20	Influenza B	B/Brisbane/60/2008 (Victoria lineage)
21	Influenza B	B/Phuket/3073/2013 (Yamagata lineage)

Table.S3. Real time RT PCR (rRT PCR) with clinical specimens (A/Chicken/Korea/LPM429/2016 (H9N2) strain).

	Clinical specimens		2 dpi					4 dpi					6 dpi				
			Ct	FICT (Ratio of TL/CL)				Ct	FICT (Ratio of TL/CL)				Ct	FICT (Ratio of TL/CL)			
				D5 pair		G10 pair			D5 pair		G10 pair			D5 pair		G10 pair	
	C	N	N/A	-	10.47	-	0.12	N/A	-	0.69	-	0.32	N/A	-	8.52	-	0.13
	C	N		-	54.30	-	4.66		-	30.20	-	6.47		-	32.74	-	2.19
	C	N		-	32.04	-	3.61		-	29.59	-	4.91		-	26.66	-	7.11
	C	1	N/A	-	5.11	-	0.06	27.91	-	13.79	-	0.06	N/A	-	7.20	-	0.00
	C	2	N/A	-	10.29	-	0.00	26.91	-	8.74	-	0.54	35.38	-	10.33	-	0.00
	C	3	N/A	-	9.37	-	0.64	N/A	-	12.13	-	0.45	N/A	-	23.26	-	0.05
	C	4	N/A	-	11.31	-	0.37	29.9	-	11.72	-	0.40	34.7	-	14.66	-	0.24
	C	5	N/A	-	11.85	-	0.03	N/A	-	4.51	-	0.91	N/A	-	18.77	-	0.12
Cloacal	C	6	N/A	-	8.18	-	0.36	N/A	-	12.85	-	1.57	37.01	-	15.01	-	0.48
	S	N	N/A	-	3.31	-	0.15	N/A	-	3.61	-	4.81	N/A	-	4.15	-	0.82
	S	N		-	2.50	-	4.17		-	5.42	-	2.55		-	4.46	-	5.30
	S	N		-	1.45	-	0.00		-	2.52	-	5.30		-	3.37	-	5.42
	S	1	37.17	+	6.82	-	0.00	29.48	-	3.12	-	1.99	33.72	+	11.80	-	2.16
	S	2	38.06	+	25.02	-	0.00	N/A	-	5.53	+	137.85	28	+	12.86	-	0.98
	S	3	N/A	+	11.67	+	6.18	N/A	-	0.53	+	6.51	26.8	+	18.98	-	0.42
	S	4	N/A	+	9.18	-	0.01	26.61	-	4.61	-	0.70	31.96	+	17.11	-	0.50
	S	5	N/A	+	11.72	-	0.22	37.32	+	10.84	-	0.60	30.33	+	10.48	-	0.02
	S	6	N/A	-	3.87	-	0.03	27.47	+	11.15	-	2.84	30.84	+	10.17	-	0.06
	S	7	38.11	-	2.99	-	2.71	35.94	+	14.13	-	5.55	33.55	+	9.25	-	0.00
Stool	S	8	N/A	+	6.83	-	0.43	20.33	-	0	+	31.47	31.79	+	17.31	-	0.74
	H9N2		22.33					23.02					21.1				
Control	Neg Ctrl		N/A					N/A					N/A				

Table.S4. Real time RT PCR (rRT PCR) with clinical specimens (A/chicken/Korea/KNUSWR09/2009(H9N2)).

	Clinical specimens		2 dpi					4 dpi					6 dpi				
			Ct	FICT (Ratio of TL/CL)				Ct	FICT (Ratio of TL/CL)				Ct	FICT (Ratio of TL/CL)			
				D5 pair		G10 pair			D5 pair		G10 pair			D5 pair		G10 pair	
Cloacal	C	NC-1	N/A	-	2.8	-	0.02	N/A	-	3.26	-	0.18	N/A	-	3.56	-	0.16
	C	NC-2	N/A	-	2.45	-	0.18	N/A	-	1.91	-	1.12	N/A	-	2.38	-	0.24
	C	NC-3	N/A	-	2.49	-	0.35	N/A	-	2.59	-	0.57	N/A	-	4.38	-	0.21
	C	H9N2-1	N/A	-	3.03	-	0.28	38.22	+	15.75	-	2.47	30.49	+	33.84	-	3.95
	C	H9N2-2	N/A	-	2.03	-	0.6771	N/A	-	3.73	-	0.76	N/A	-	4.52	-	0.05
	C	H9N2-3	38.89	-	2.87	-	0.40	39.26	-	2.34	-	0.15	32.79	+	7.24	-	0.08
Stool	S	NC-1	N/A	-	2.97	-	0.17	N/A	-	1.09	-	0.75	N/A	-	3.44	-	0.28
	S	NC-2	N/A	-	2.73	-	0.06	N/A	-	0.09	-	0.12	N/A	-	3.55	-	0.26
	S	NC-3	N/A	-	2.20	-	1.32	N/A	-	1.49	-	0.58	N/A	-	3.42	-	1.97
	S	H9N2-1	35.02	-	3.48	-	0.60	31.44	+	1094.3	+	394.16	23.1	+	675.84	+	466.25
	S	H9N2-2	N/A	-	3.01	-	0.71	33.74	-	1.07	-	3.09	27.14	+	7.61	-	0.91
	S	H9N2-3	N/A	-	2.53	-	0.24	35.35	+	15.92	-	0.21	26	+	866.35	+	125.12
Control	H9N2		19.24														
	Neg Ctrl		N/A														

A

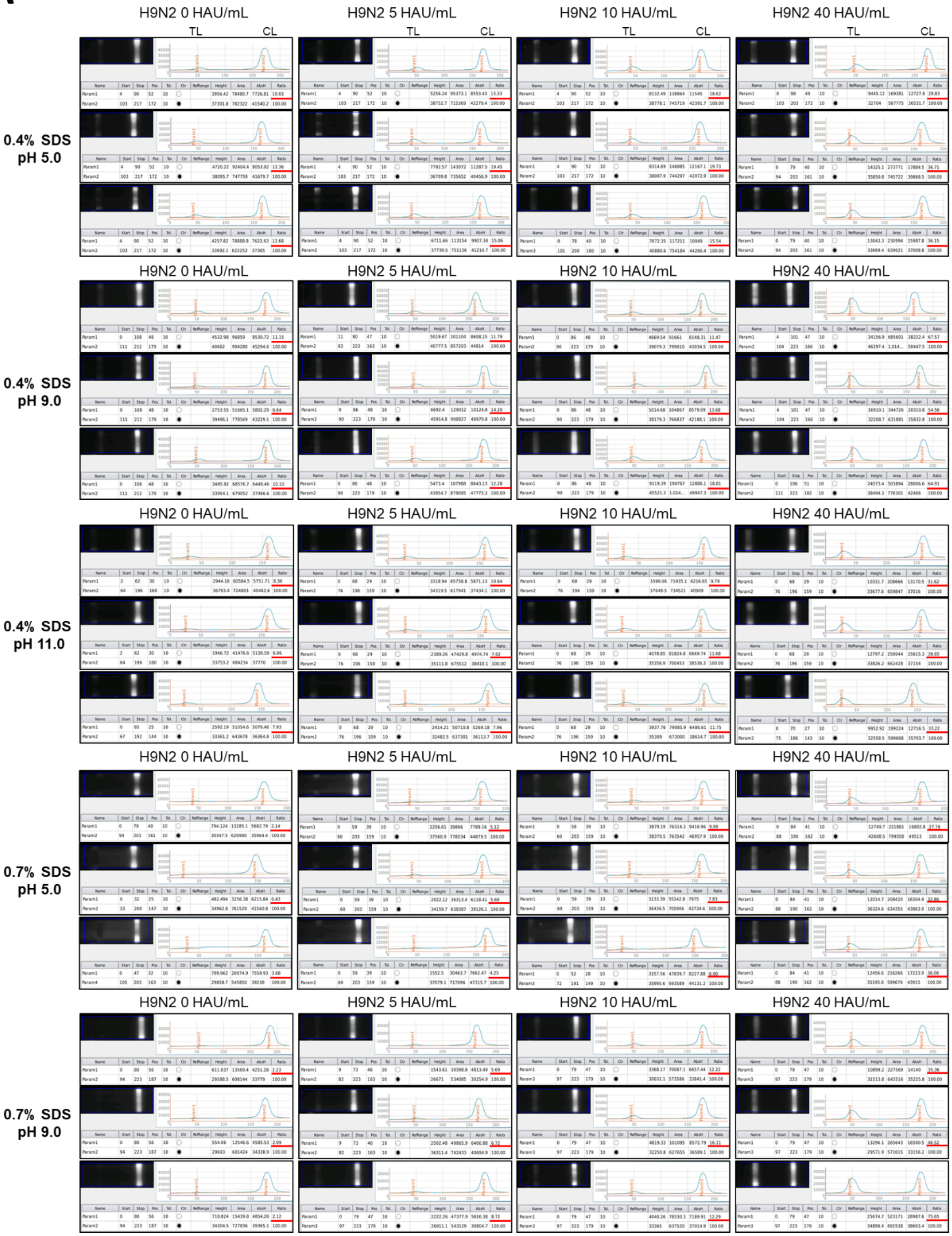

H9N2 0 HAU/mL
H9N2 5 HAU/mL
H9N2 10 HAU/mL
H9N2 40 HAU/mL
TL
CL
0.4% SDS pH 5.0
0.4% SDS pH 9.0
0.4% SDS pH 11.0
0.7% SDS pH 5.0
0.7% SDS pH 9.0

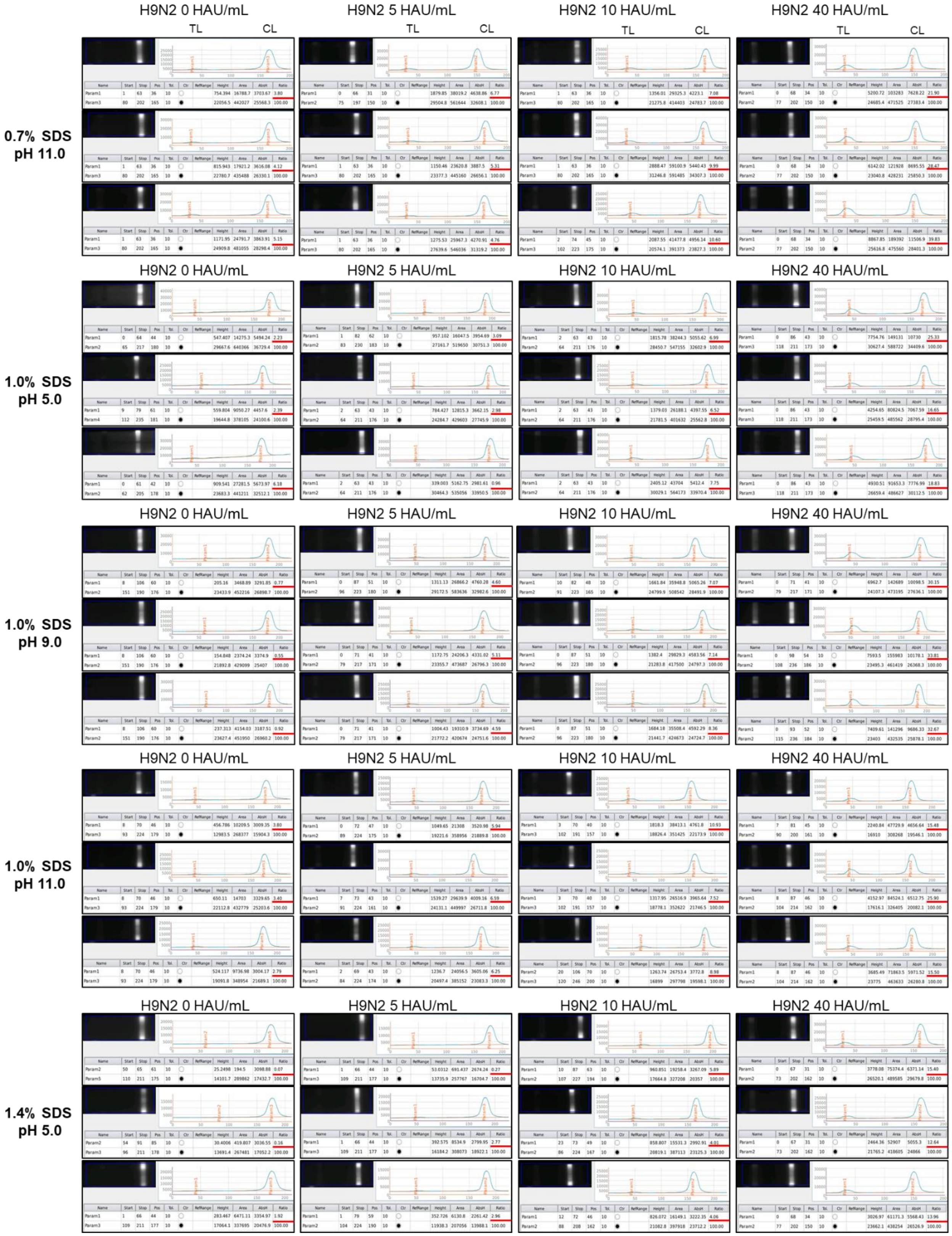
H9N2 0 HAU/mL
H9N2 5 HAU/mL
H9N2 10 HAU/mL
H9N2 40 HAU/mL
TL
CL
0.7% SDS pH 11.0
1.0% SDS pH 5.0
1.0% SDS pH 9.0
1.0% SDS pH 11.0
1.4% SDS pH 5.0

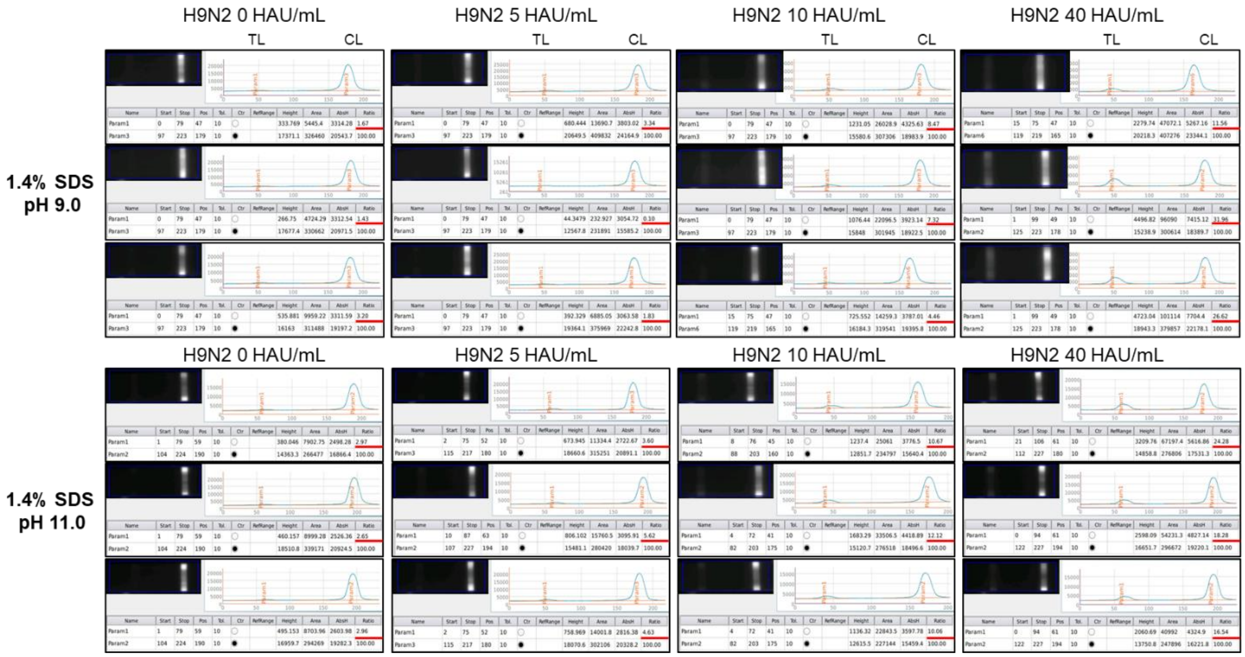
H9N2 0 HAU/mL
H9N2 5 HAU/mL
H9N2 10 HAU/mL
H9N2 40 HAU/mL
TL
CL
1.4% SDS pH 9.0
1.4% SDS pH 11.0

B

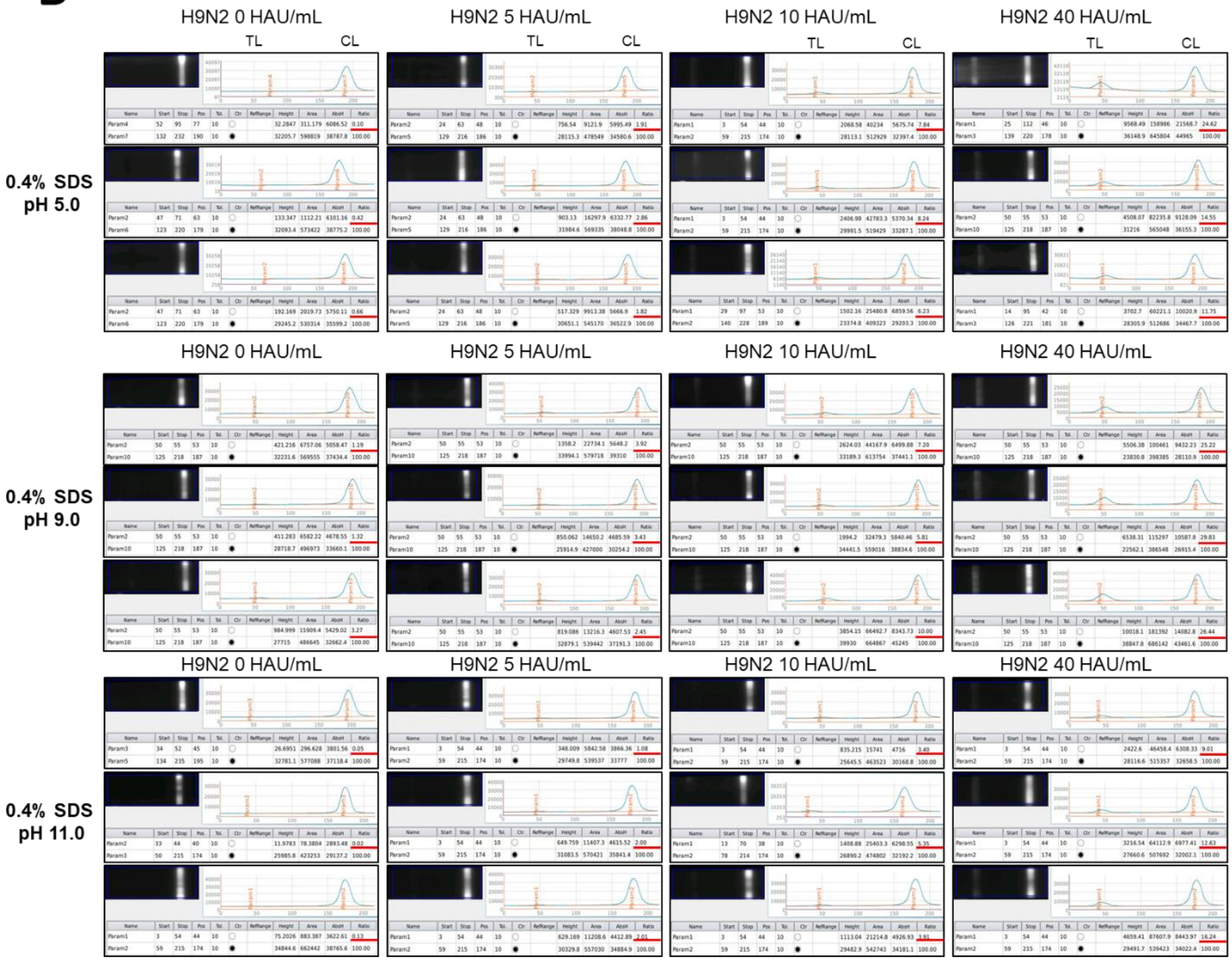
H9N2 0 HAU/mL
H9N2 5 HAU/mL
H9N2 10 HAU/mL
H9N2 40 HAU/mL
TL
CL
0.4% SDS pH 5.0
0.4% SDS pH 9.0
0.4% SDS pH 11.0

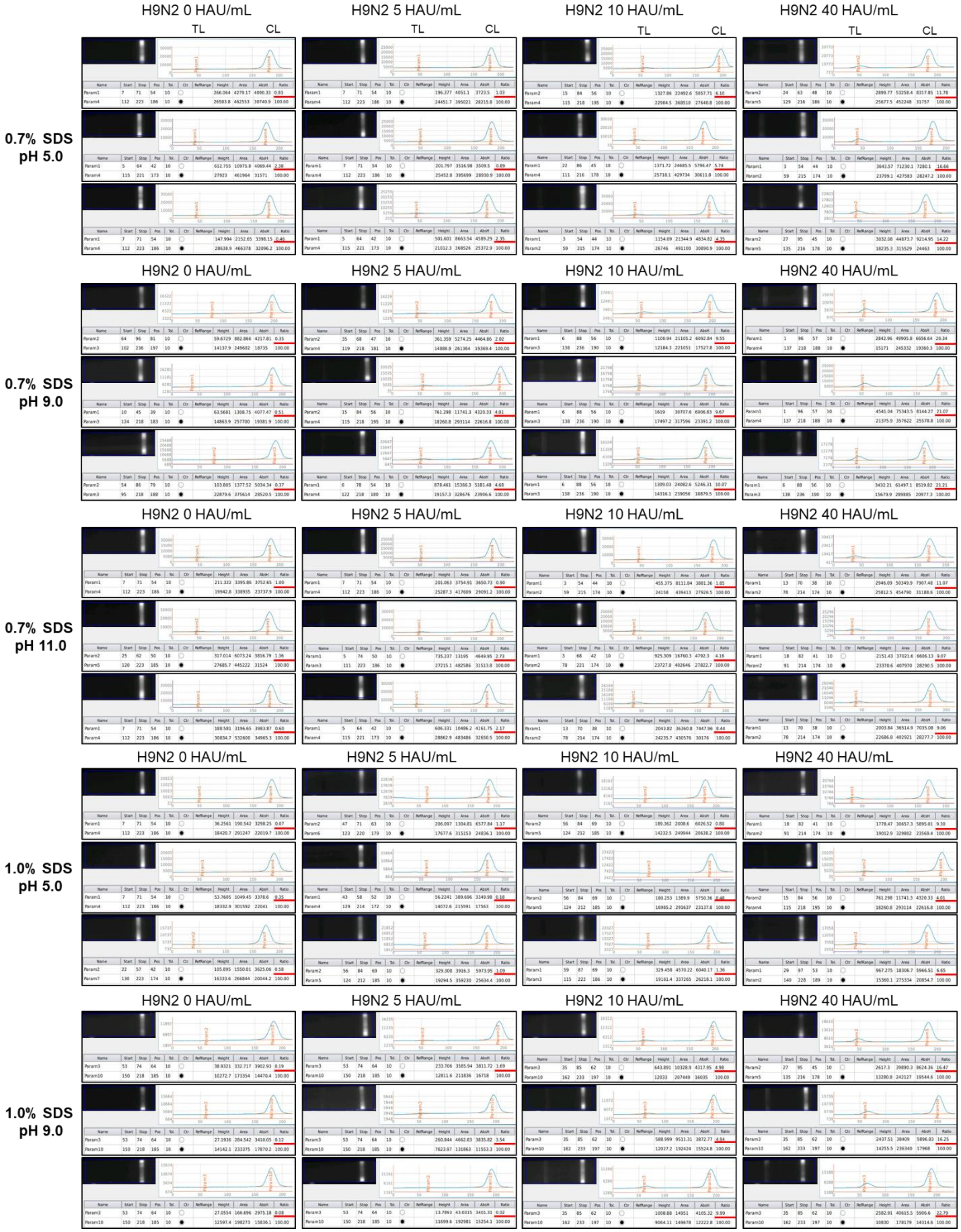
H9N2 0 HAU/mL
H9N2 5 HAU/mL
H9N2 10 HAU/mL
H9N2 40 HAU/mL
TL
CL
0.7% SDS
pH 5.0
0.7% SDS
pH 9.0
0.7% SDS
pH 11.0
1.0% SDS
pH 5.0
1.0% SDS
pH 9.0

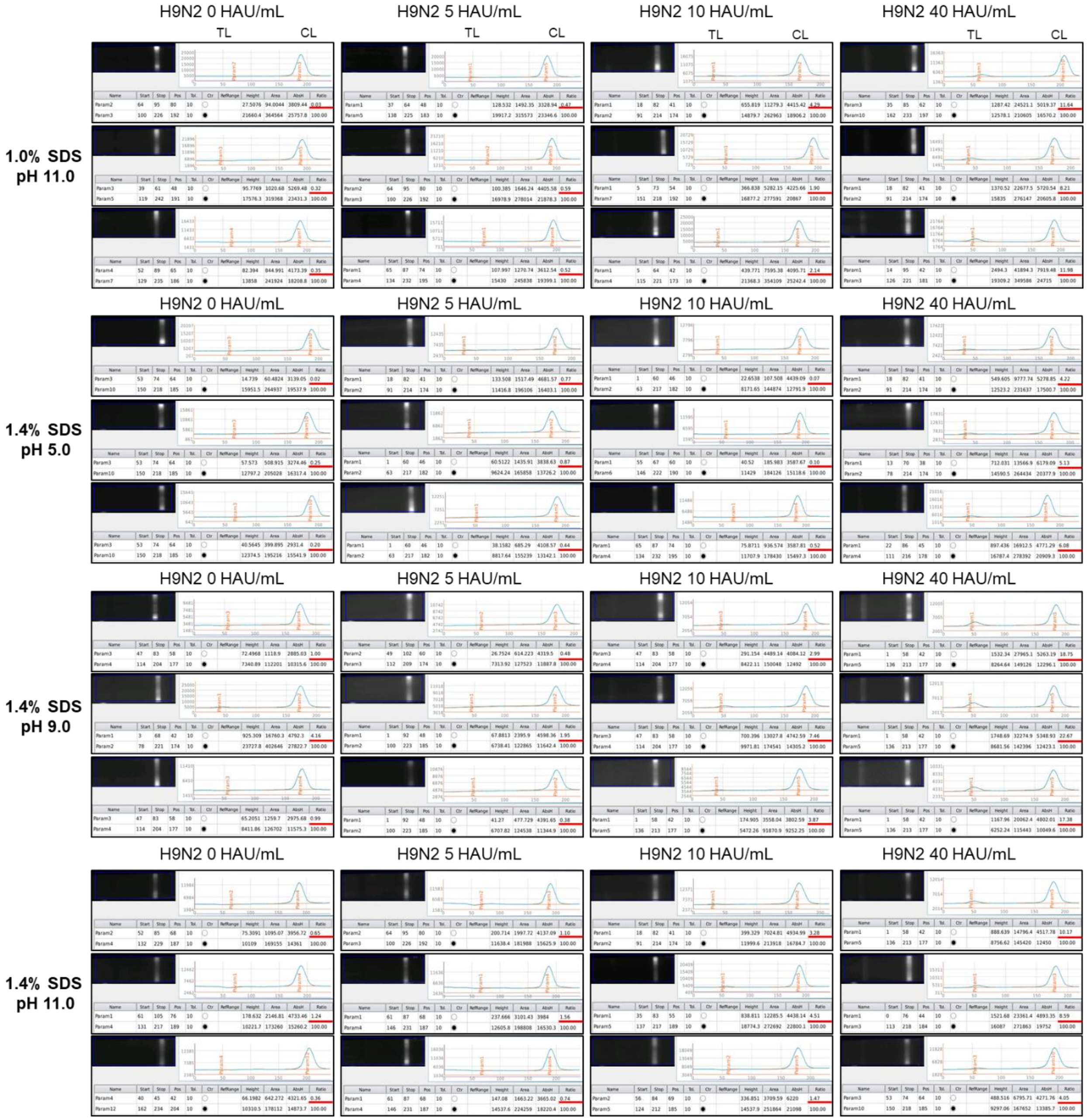

Fig.S1. Raw data of lysis buffer optimization. (A) Europium A45-D5 FICT, (B) Europium A39-G10 FICT. TL/CL is underlined with red color. (H9N2: A/chicken/Korea/KNUSWR09/2009(H9N2))

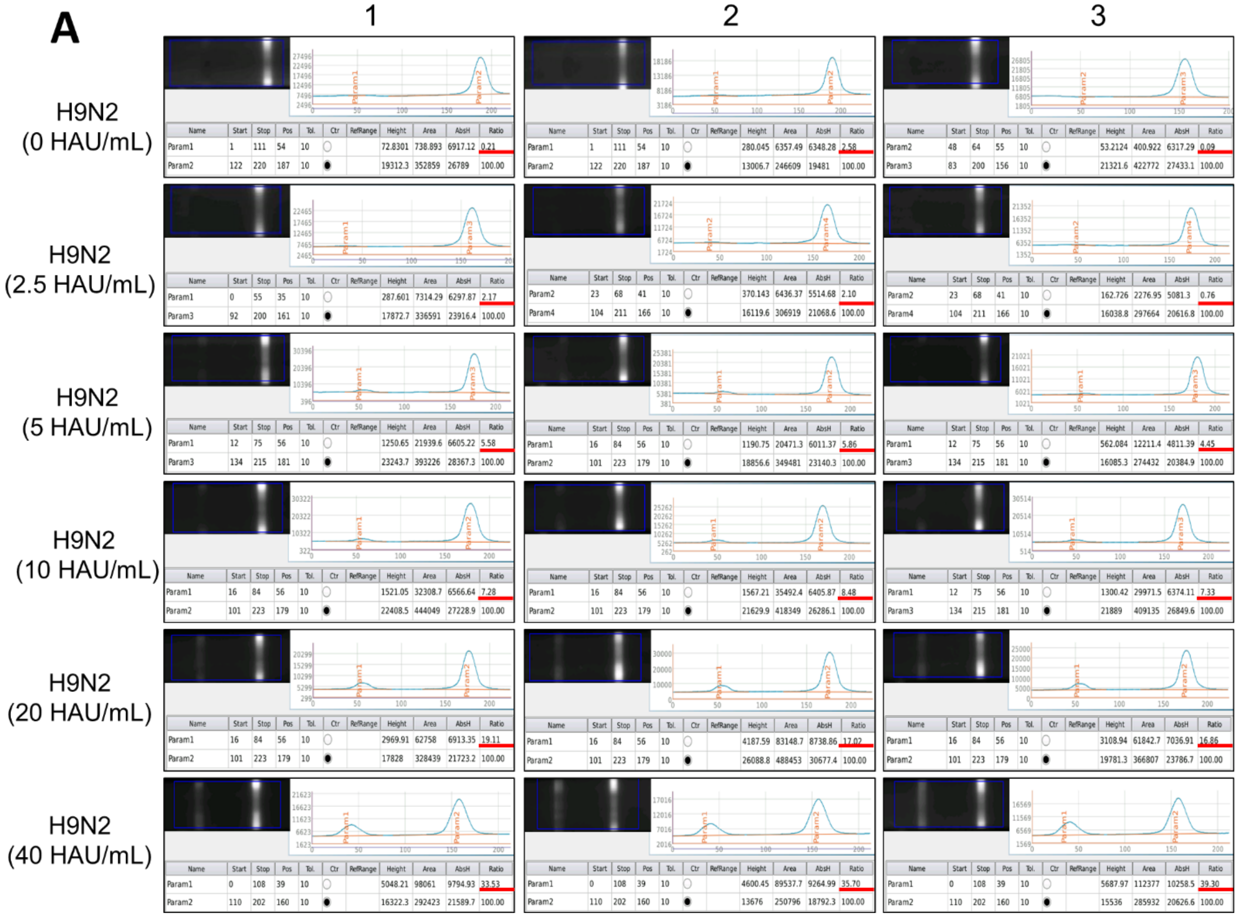
A
1
2
3
H9N2
(0 HAU/mL)
H9N2
(2.5 HAU/mL)
H9N2
(5 HAU/mL)
H9N2
(10 HAU/mL)
H9N2
(20 HAU/mL)
H9N2
(40 HAU/mL)

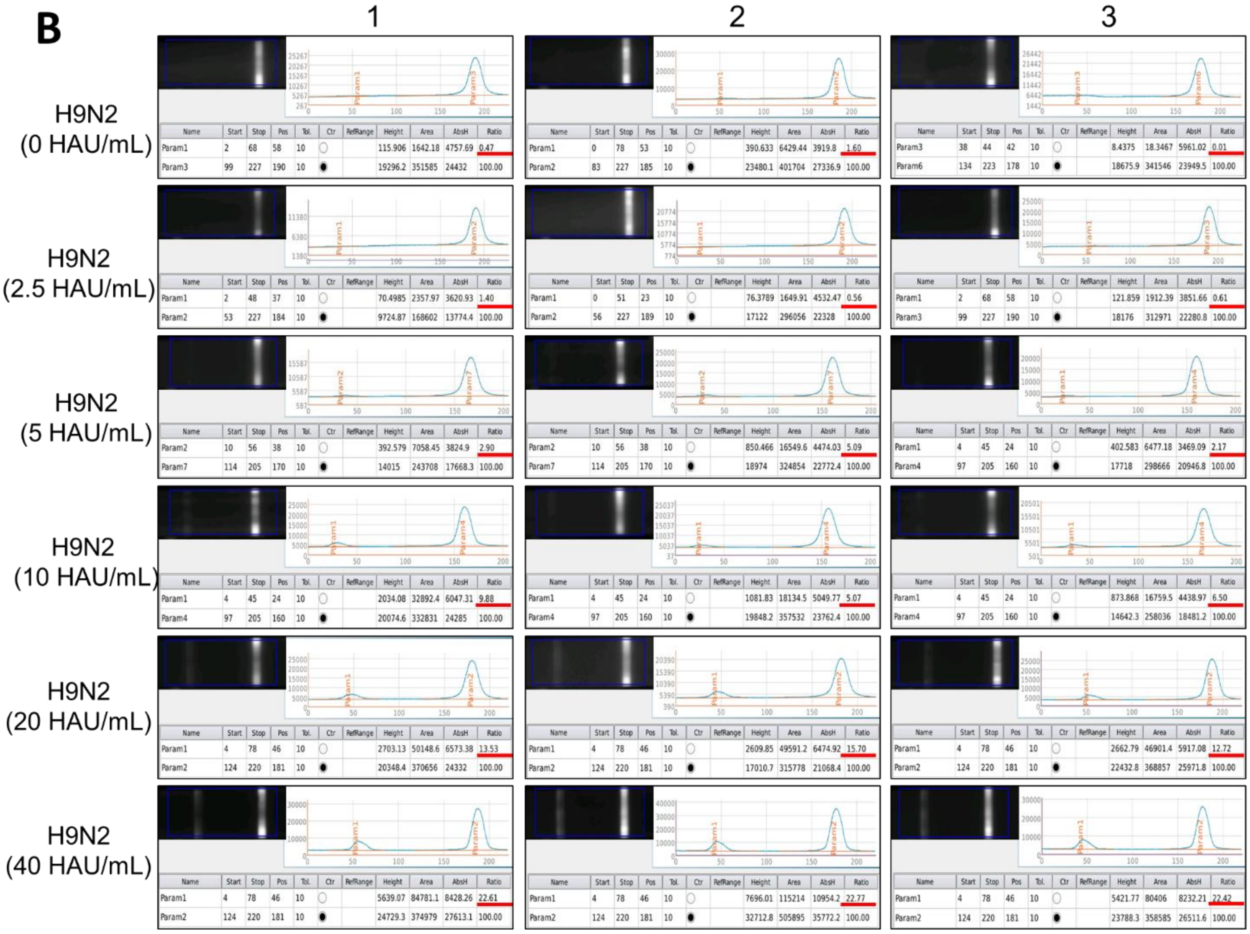

Fig.S2. Raw data of limit of detection for spiked-H9N2 virus in distilled water (DW). (A) Europium A45-D5 FICT, (B) Europium A39-G10 FICT. TL/CL is underlined with red color. (H9N2: A/chicken/Korea/KNUSWR09/2009(H9N2))

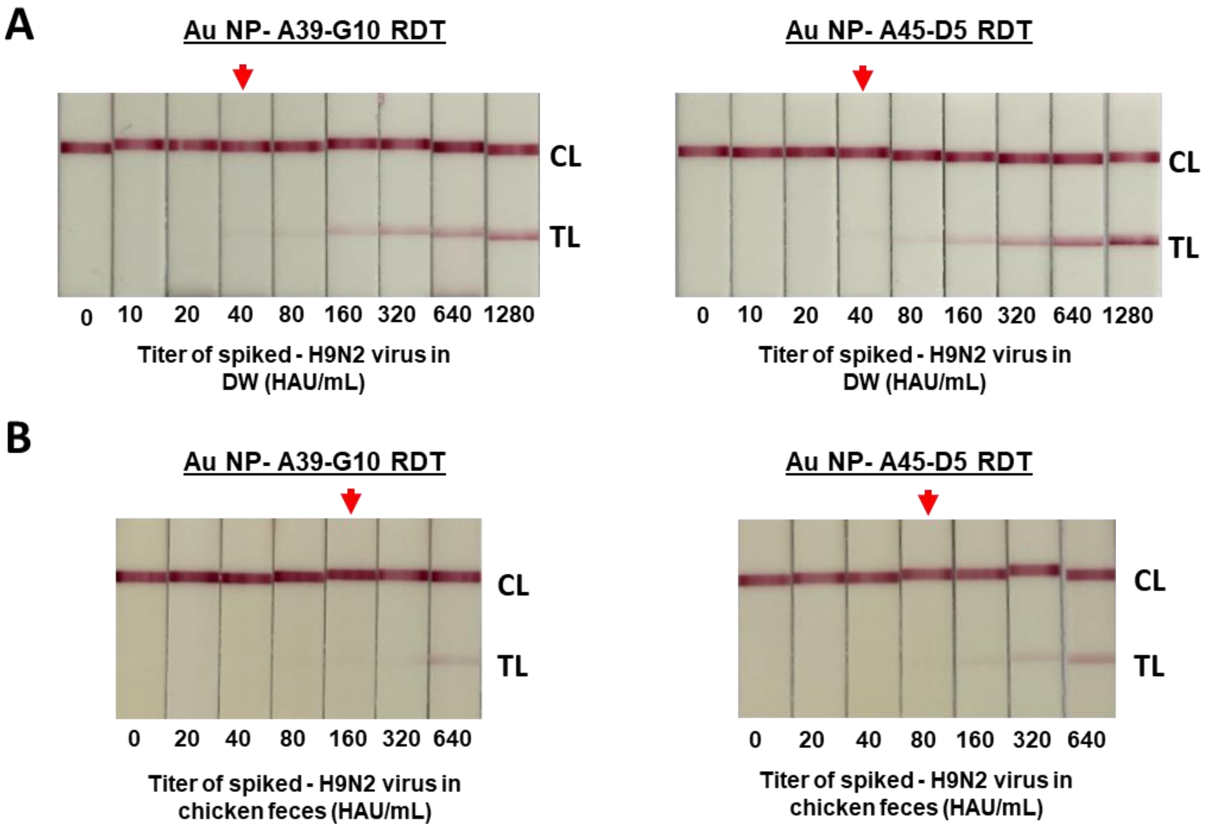

Fig.S3. Limit of detection of conventional rapid diagnostic test (RDT). The A45-D5 and A39-G10 McAbs conjugated with colloidal gold nanoparticle (Au NP) was captured on the TL (A27-9 McAb) if there was virus during lateral flow reaction. After 20 min, the strip was read with the naked eye for a binary decision. Detection limit of FICT assay targeting influenza A virus H9N2 hemaglutinin (HA) were tested with the two-fold serially diluted from 10 to 1280 HAU/mL in deionized sterile water (DW) (A) and the two-fold serially diluted from 10 to 1280 HAU/mL in in normal chicken feces (B). The arrow indicates the LOD.

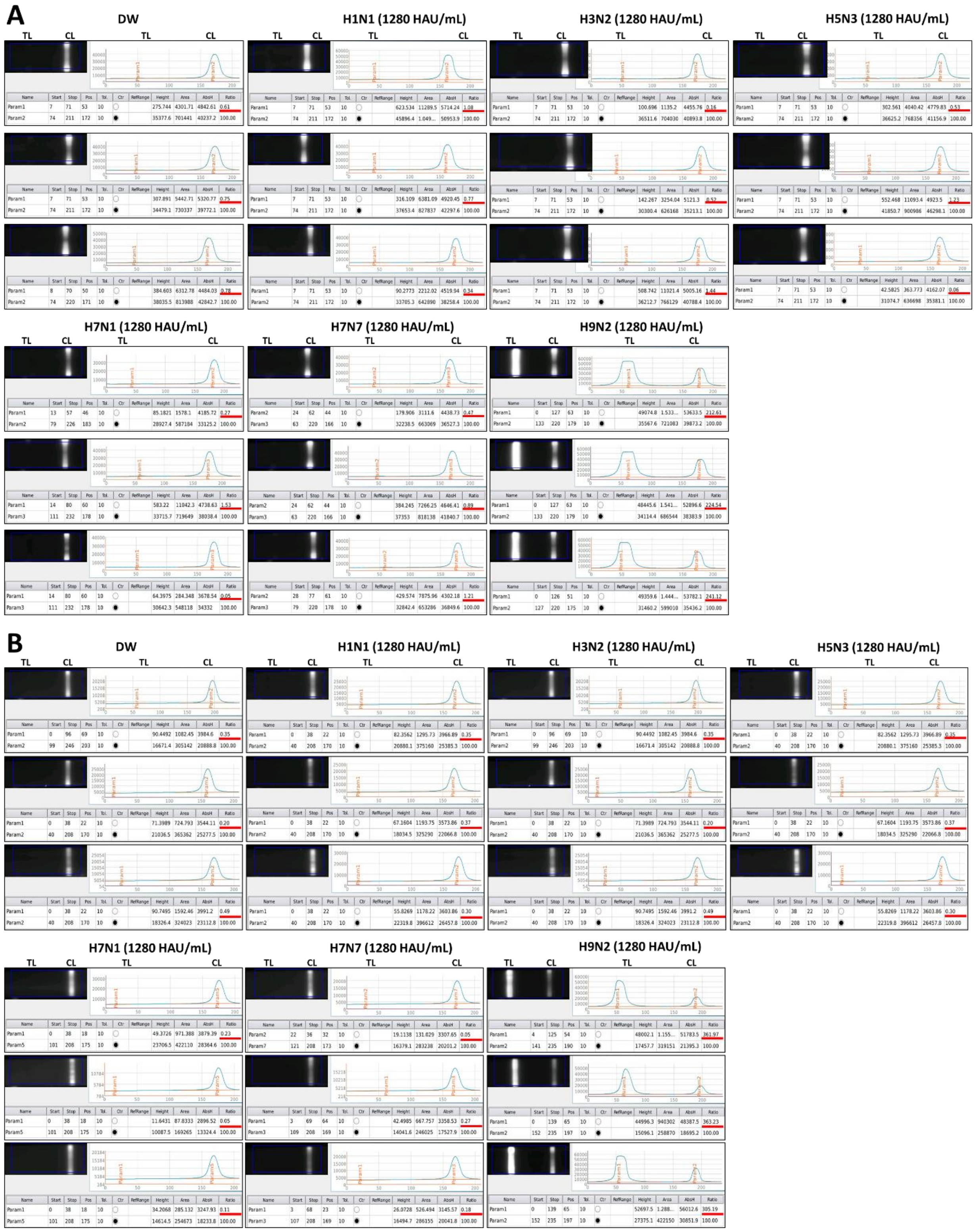

Fig.S4. Raw data of specificity for spiked-H9N2 virus in DW. (A) Europium A45-D5 FICT, (B) Europium A39-G10 FICT. TL/CL is underlined with red color.

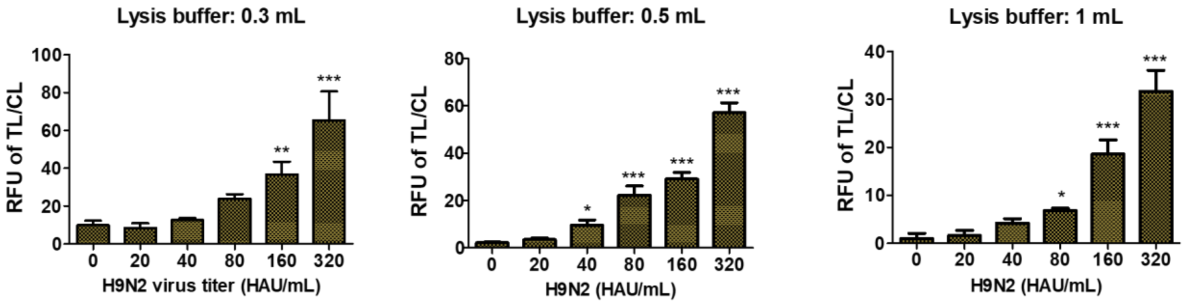

Lysis buffer: 0.3 mL
RFU of TL/CL
H9N2 virus titer (HAU/mL)
0
20
40
80
160
320
**
***
Lysis buffer: 0.5 mL
RFU of TL/CL
H9N2 (HAU/mL)
*
***
***
***
Lysis buffer: 1 mL
RFU of TL/CL
H9N2 (HAU/mL)
*
***
***

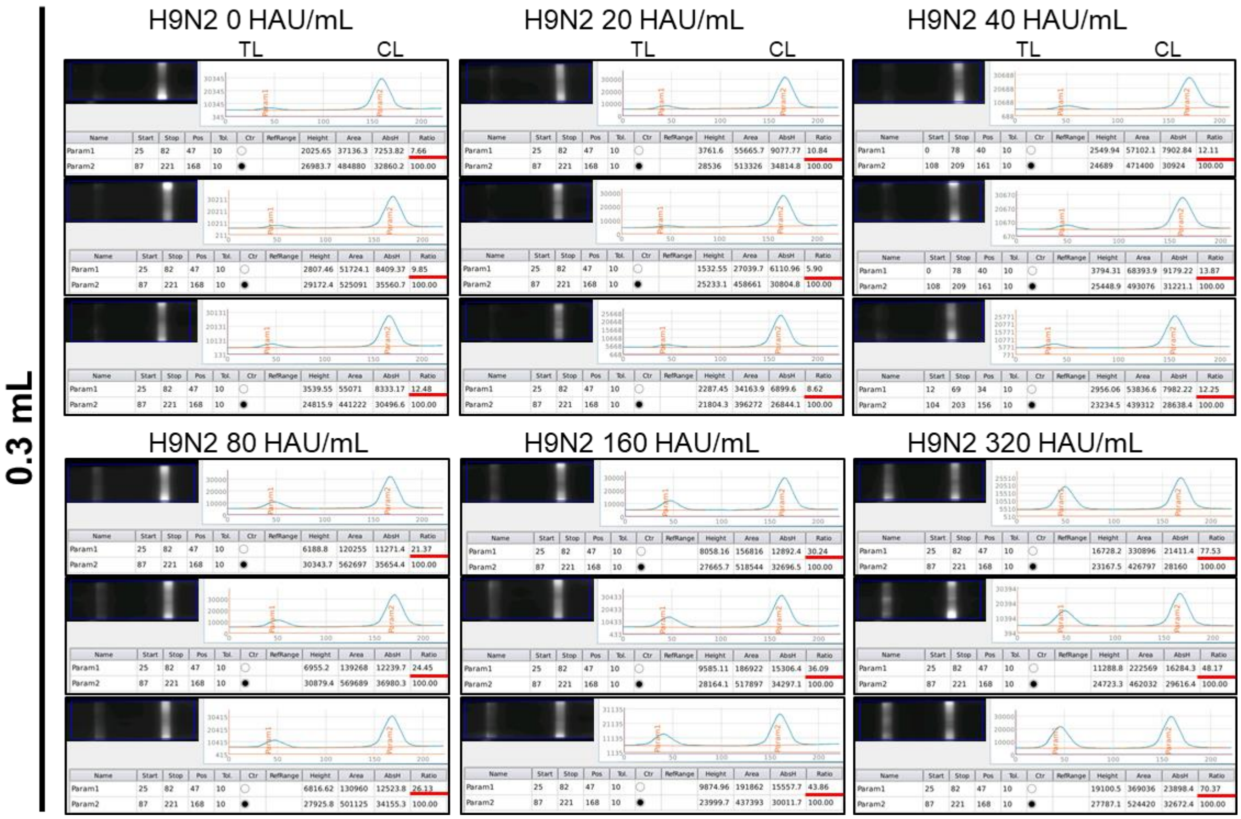

0.3 mL
H9N2 0 HAU/mL
H9N2 20 HAU/mL
H9N2 40 HAU/mL
TL
CL
H9N2 80 HAU/mL
H9N2 160 HAU/mL
H9N2 320 HAU/mL

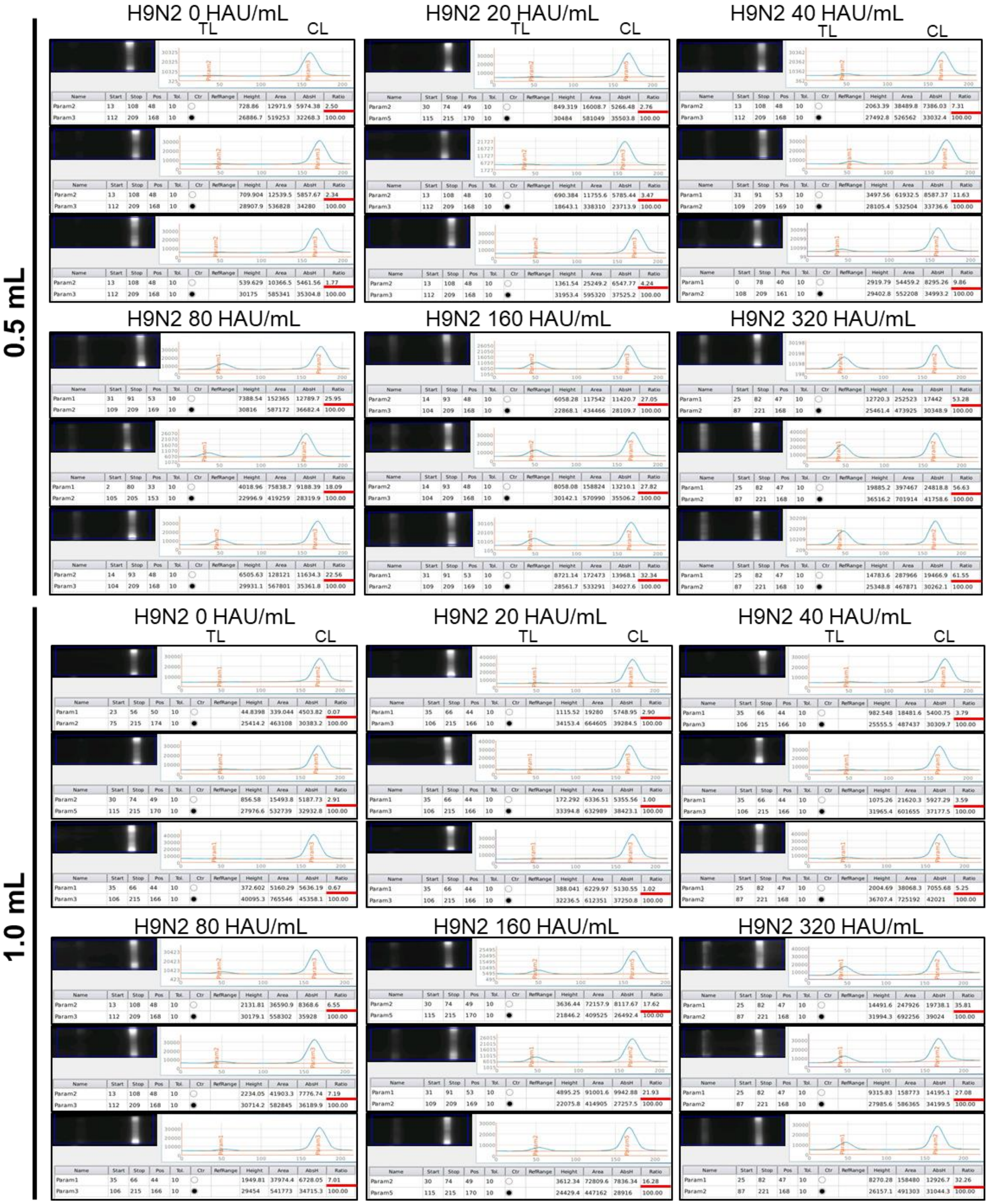

Fig.S5: Optimization amount of lysis buffer for feces condition. Lysis buffer (0.1M Tris pH9.0, 0.1M EDTA, 1.0%SDS and 0.5% Triton X-100) with various volume (0.3mL, 0.5mL and 1mL) were tested. *, *P < 0.05; ***, *P < 0.01; ****, *P < 0.001*. TL/CL is underlined with red color. (H9N2: A/chicken/Korea/KNUSWR09/2009(H9N2))

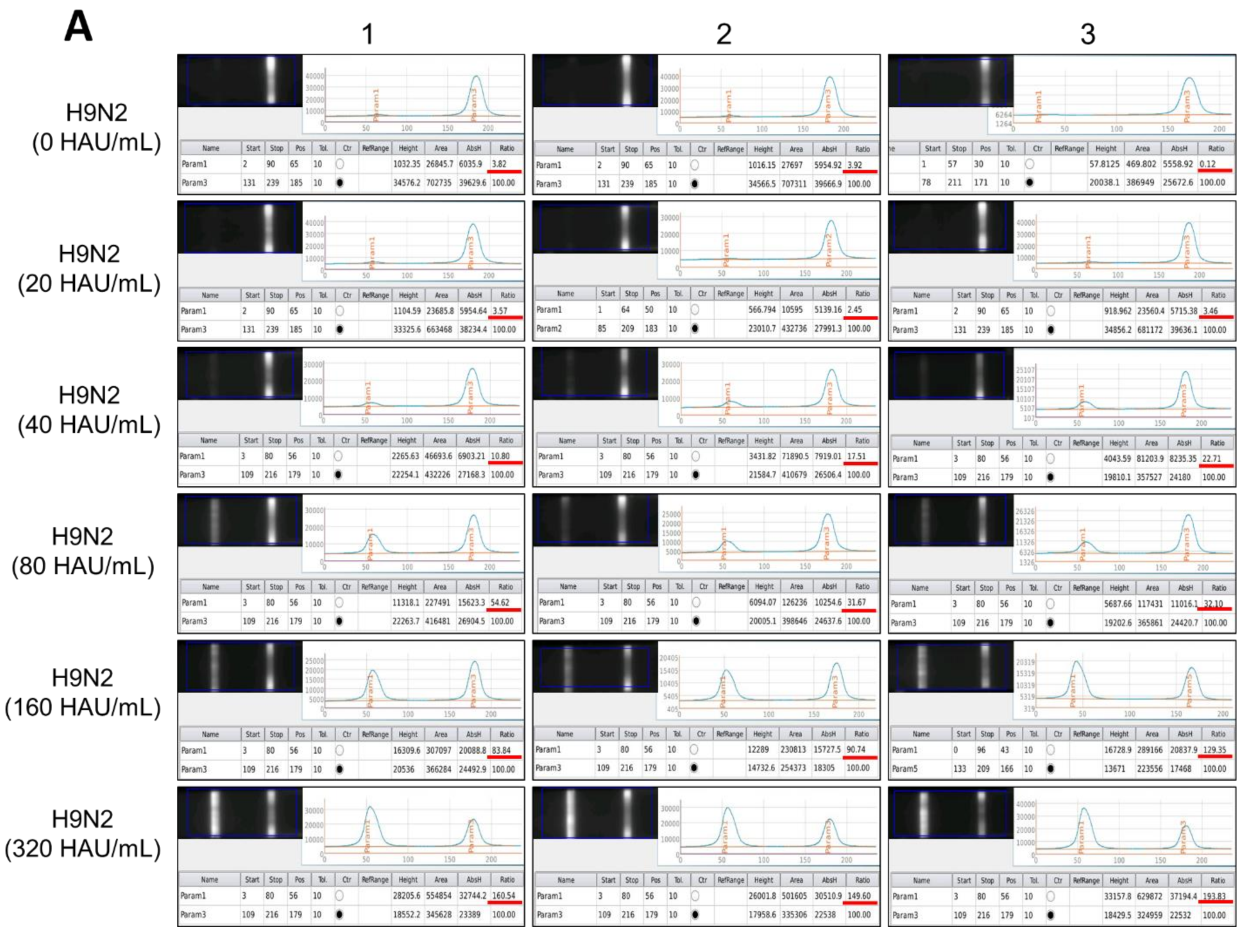

A
1
2
3
H9N2
(0 HAU/mL)
H9N2
(20 HAU/mL)
H9N2
(40 HAU/mL)
H9N2
(80 HAU/mL)
H9N2
(160 HAU/mL)
H9N2
(320 HAU/mL)

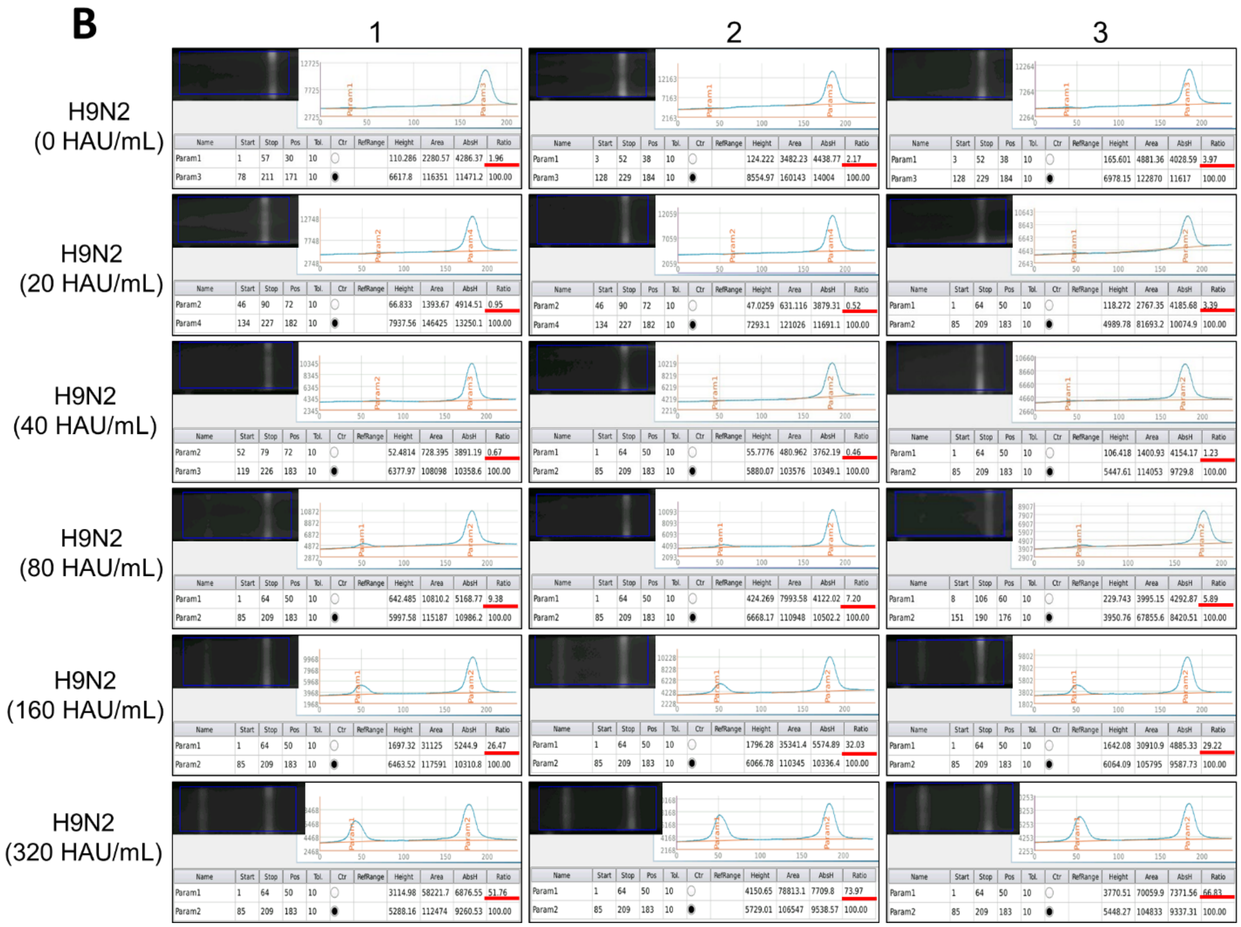

Fig.S6. Raw data of limit of detection for spiked-virus in feces. (A) Europium A45-D5 FICT, (B) Europium A39-G10 FICT. TL/CL is underlined with red color. (H9N2: A/chicken/Korea/KNUSWR09/2009(H9N2))

A

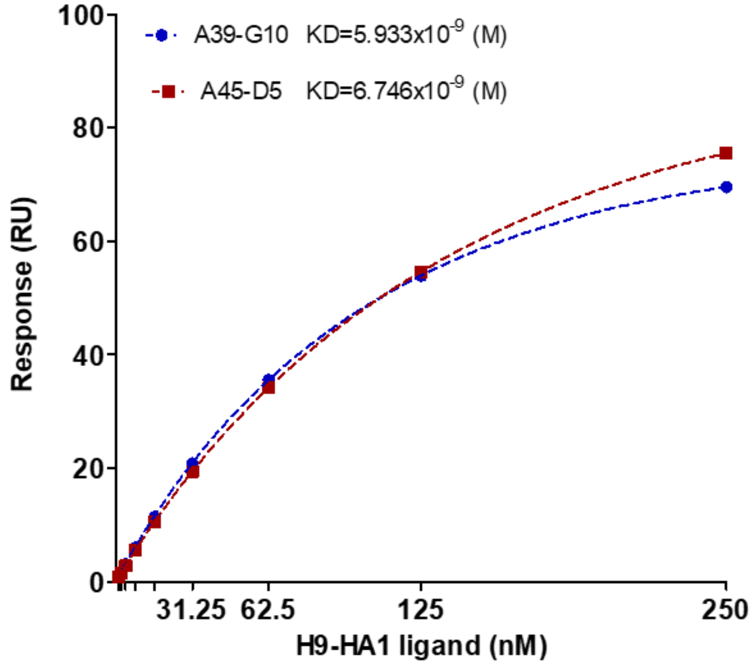

Ligand	Analyte	Conc.	ka (1/Ms)	kd (1/s)	KD (M)	Rmax (RU)	Chi^2 (RU^2)
H9-HA1	A39-G10	0.977, 1.953, 3.906, 7.813, 15.625, 31.25, 62.5, 125, 250 nM	4.641E+04	2.753E-04	5.933E-09	88.84	2.16
	A45-D5	0.977, 1.953, 3.906, 7.813, 15.625, 31.25, 62.5, 125, 250 nM	3.513E+04	2.370E-04	6.746E-09	102.2	1.96

KD (Equilibrium dissociation rate constant)

ka (association rate constant)

kd (dissociation rate constant)

B

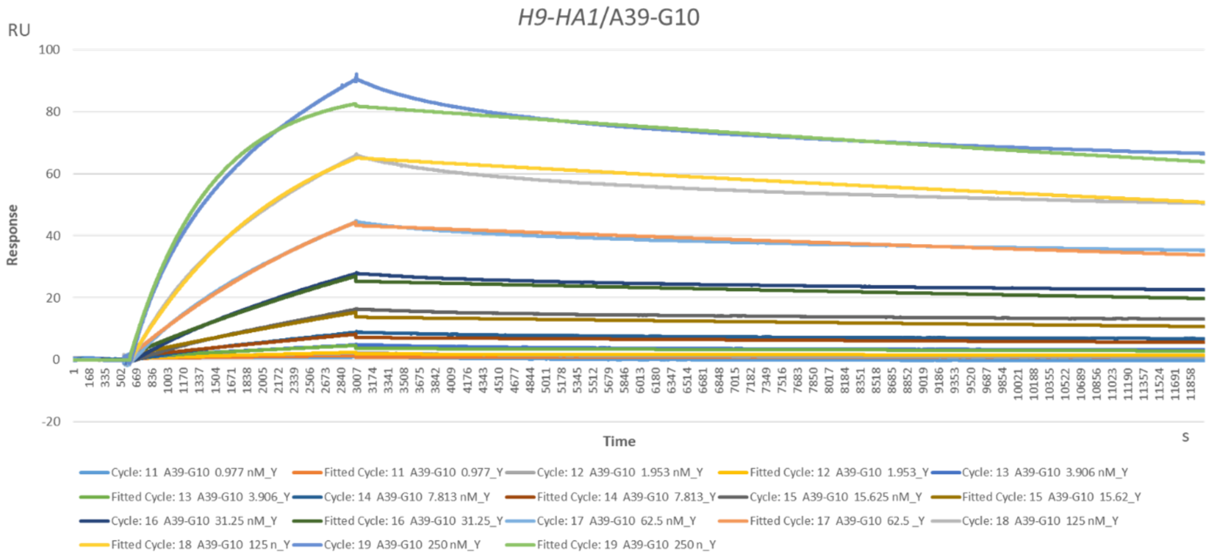

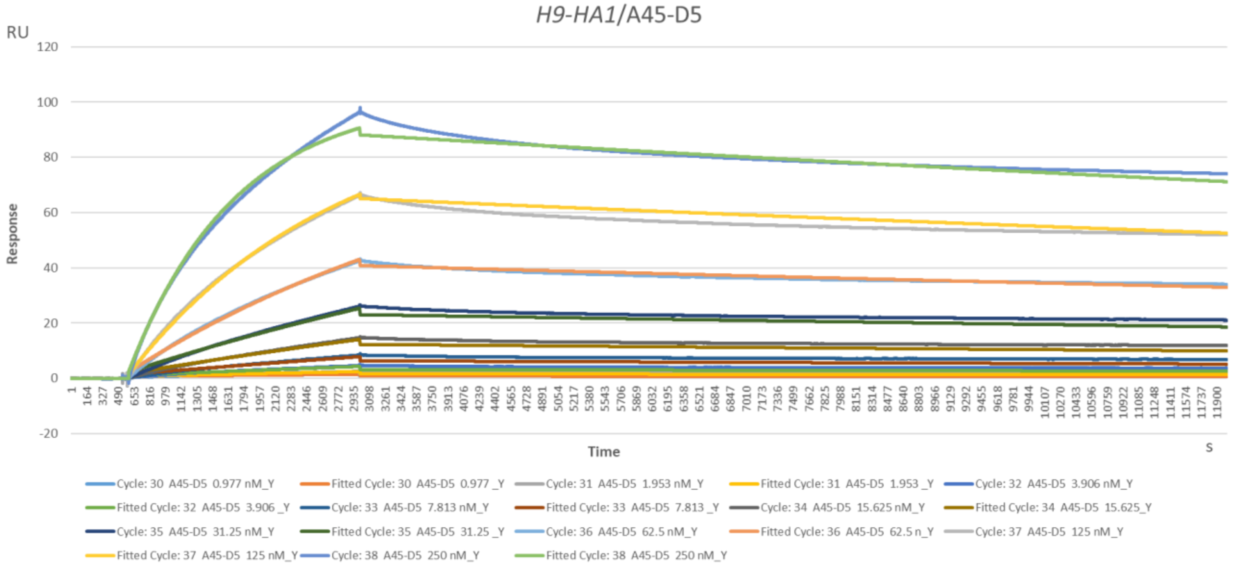

Fig.S7. Measurement of KD values for two antibodies (A39-G10 and A45-D5) by surface plasmon resonance (SPR) with Biacore T200 (GE Healthcare (Sweden) of WOOJUNG BIO, Inc Company. (A) The H9N2 HA1 recombinant protein express in E.coli system was used for KD measurement. The Graph was re-draw with raw data by GraphPad Prism5.0. (B) Raw data of KD values measurement.

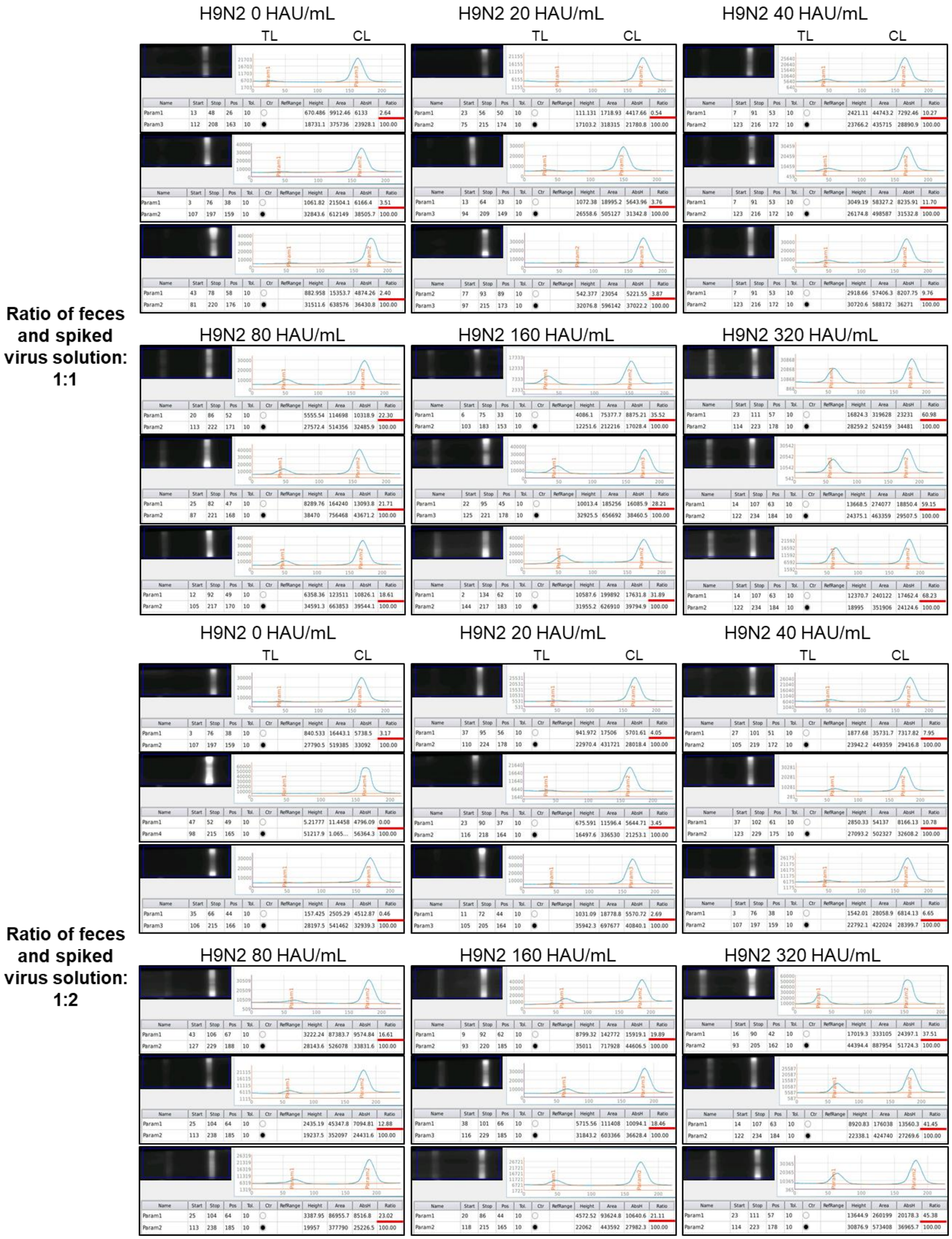
H9N2 0 HAU/mL
H9N2 20 HAU/mL
H9N2 40 HAU/mL
TL
CL
Ratio of feces and spiked virus solution: 1:1
H9N2 80 HAU/mL
H9N2 160 HAU/mL
H9N2 320 HAU/mL
H9N2 0 HAU/mL
H9N2 20 HAU/mL
H9N2 40 HAU/mL
TL
CL
Ratio of feces and spiked virus solution: 1:2
H9N2 80 HAU/mL
H9N2 160 HAU/mL
H9N2 320 HAU/mL

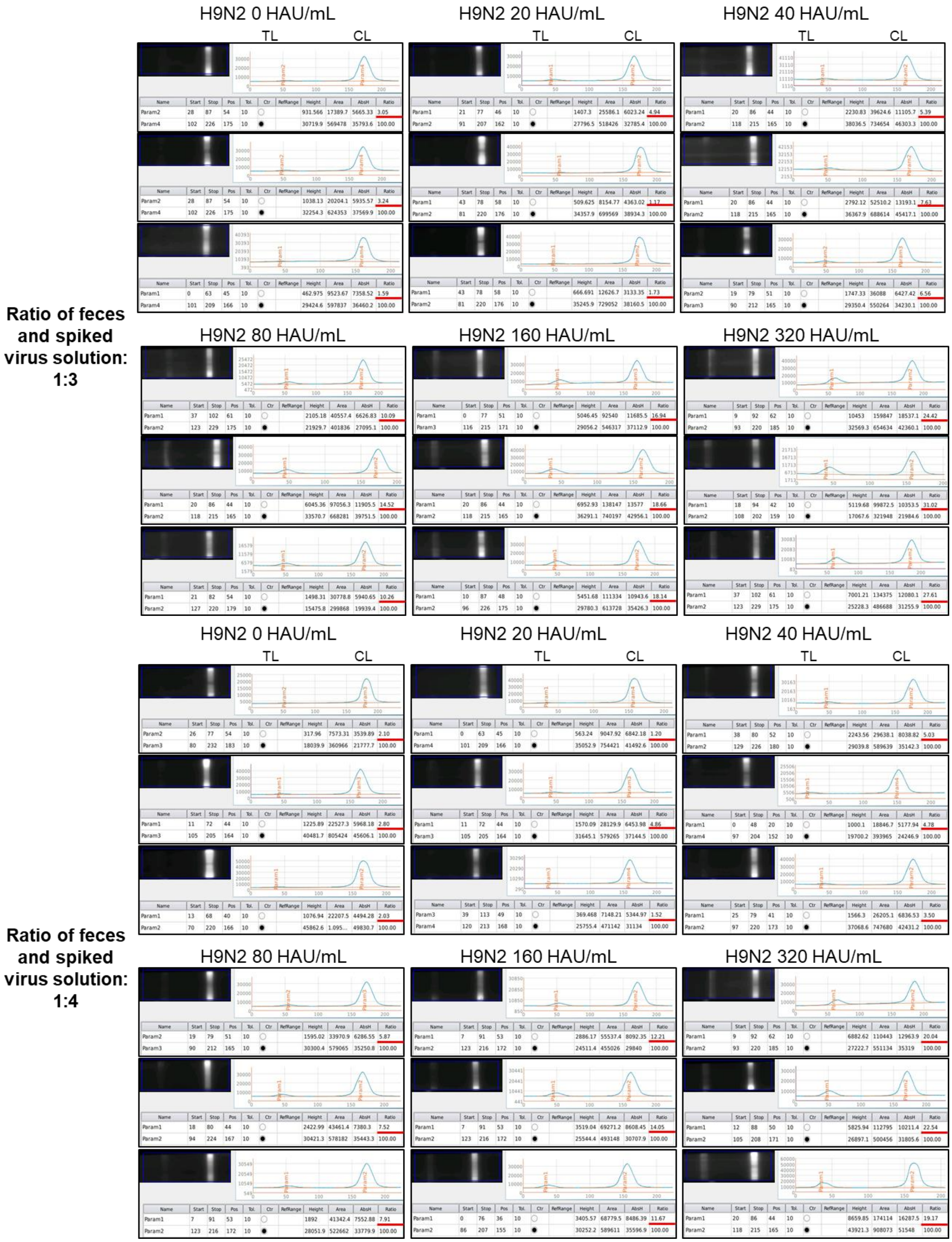

Fig.S8. Raw data of limit of feces amount to keep FICT performance. TL/CL is underlined with red color. (H9N2: A/chicken/Korea/KNUSWR09/2009(H9N2))

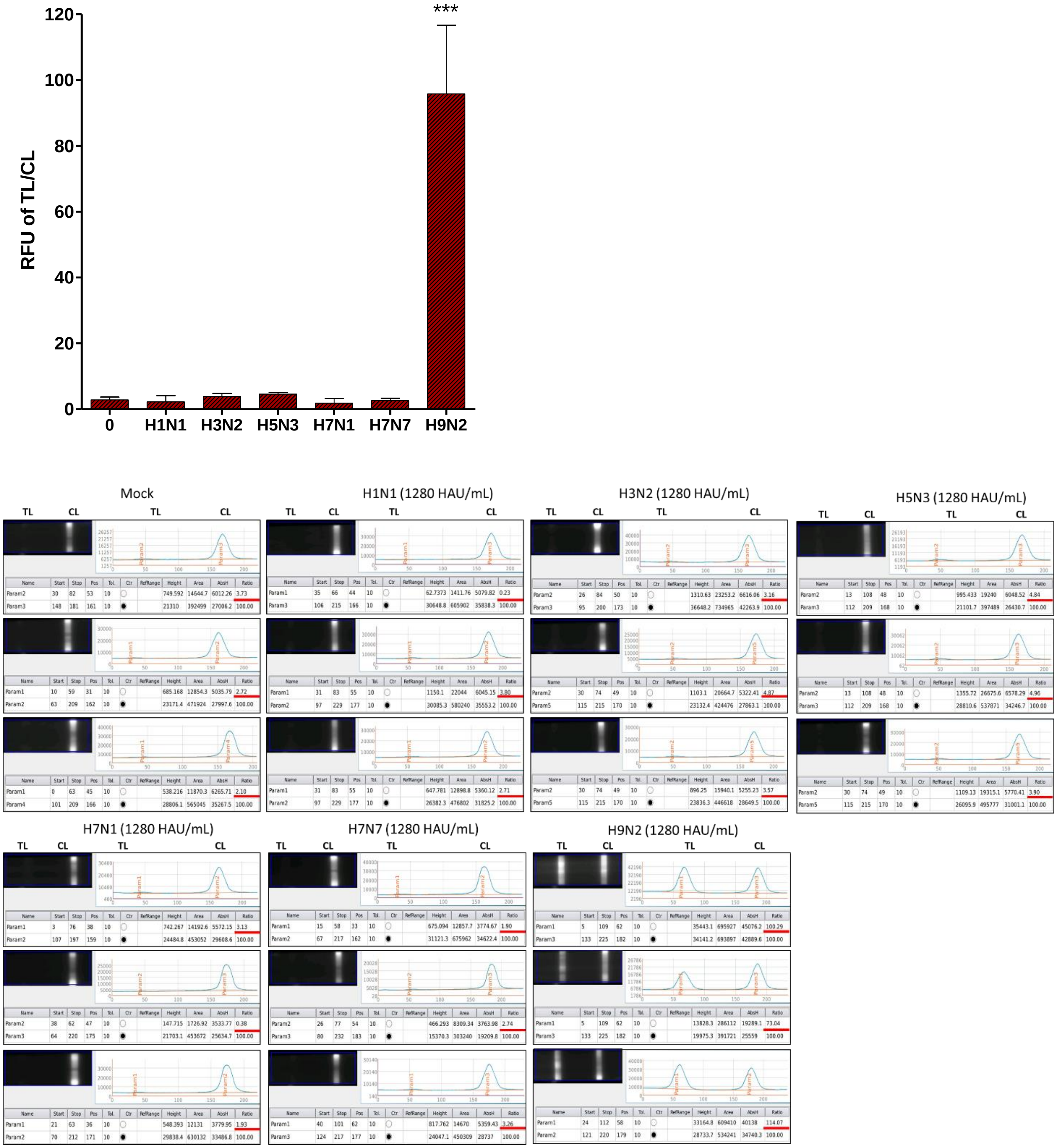

Fig.S9. The specificity of FICT for maximum feces condition. The specificity of FICT was evaluated by using influenza A virus in Wonkwang (H1N1, H3N2, H5N3, H7N1, H7N7 and H9N2) that spiked in chicken feces which were applied in high titer (1280 HAU/mL). ***, $P < 0.001$**.** TL/CL is underlined with red color.

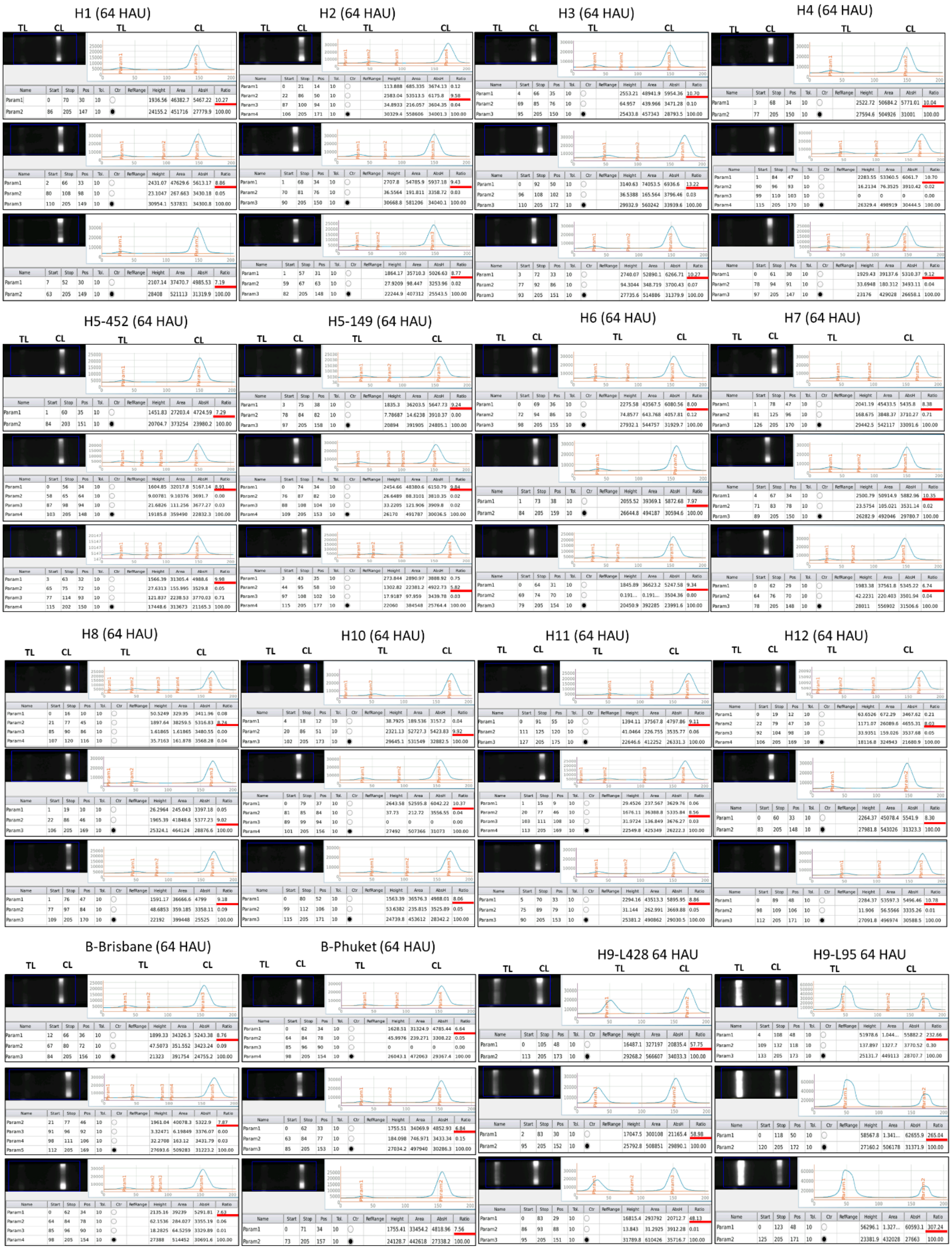
H1 (64 HAU)
H2 (64 HAU)
H3 (64 HAU)
H4 (64 HAU)
H5-452 (64 HAU)
H5-149 (64 HAU)
H6 (64 HAU)
H7 (64 HAU)
H8 (64 HAU)
H10 (64 HAU)
H11 (64 HAU)
H12 (64 HAU)
B-Brisbane (64 HAU)
B-Phuket (64 HAU)
H9-L428 64 HAU
H9-L95 64 HAU

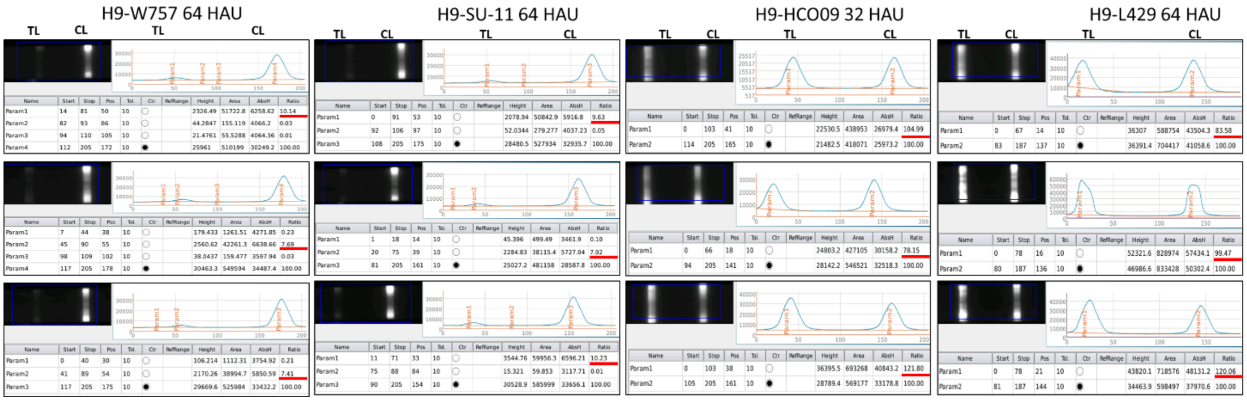

Fig.S10. Raw data of specificity of FICT for influenza A/B virus in Chungbuk. TL/CL is underlined with red color.

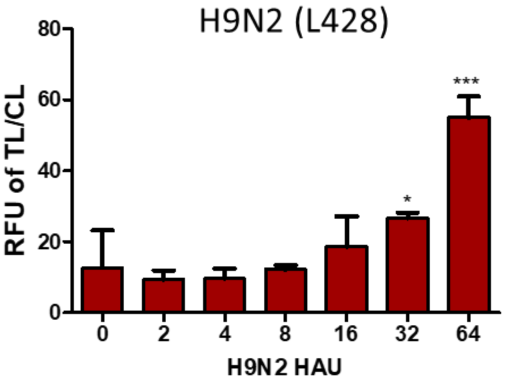
H9N2 (L428)
RFU of TL/CL
80
60
40
20
0
***
*
0
2
4
8
16
32
64
H9N2 HAU

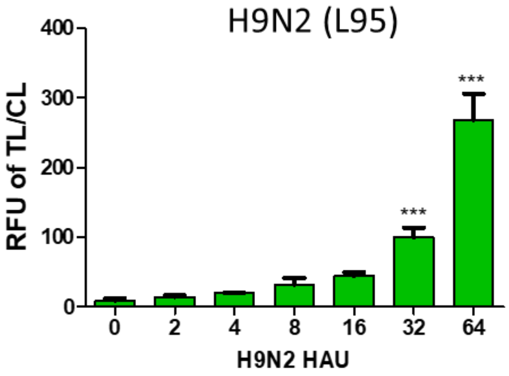
H9N2 (L95)
RFU of TL/CL
400
300
200
100
0
***
***
0
2
4
8
16
32
64
H9N2 HAU

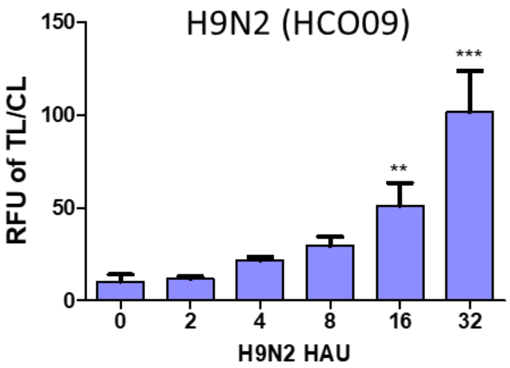
H9N2 (HCO09)
RFU of TL/CL
150
100
50
0
***
**
0
2
4
8
16
32
H9N2 HAU

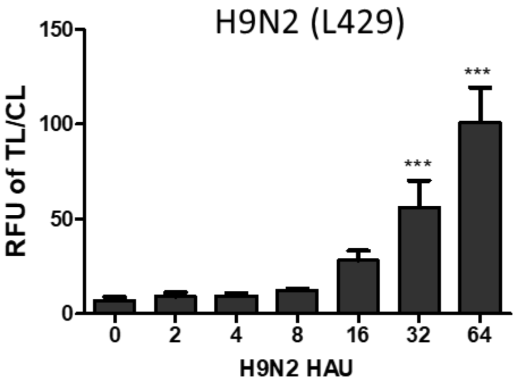
H9N2 (L429)
RFU of TL/CL
150
100
50
0
***
***
0
2
4
8
16
32
64
H9N2 HAU

A

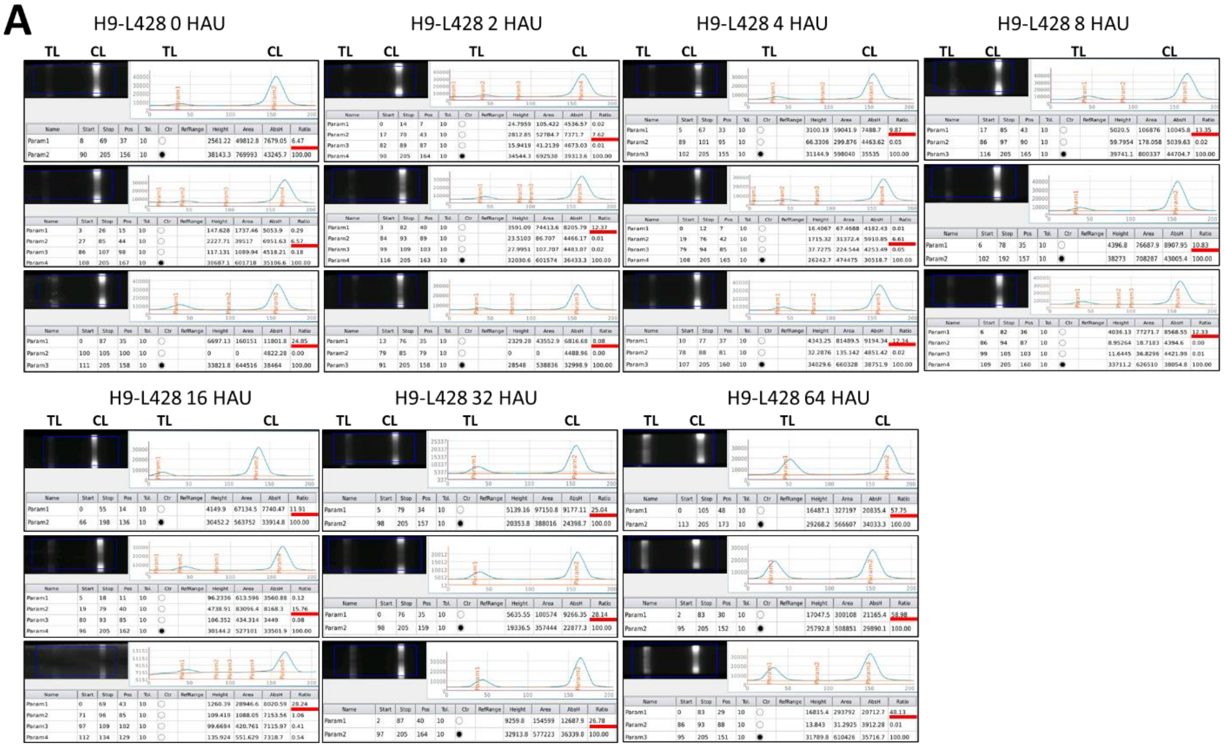
H9-L428 0 HAU
H9-L428 2 HAU
H9-L428 4 HAU
H9-L428 8 HAU
H9-L428 16 HAU
H9-L428 32 HAU
H9-L428 64 HAU
TL
CL

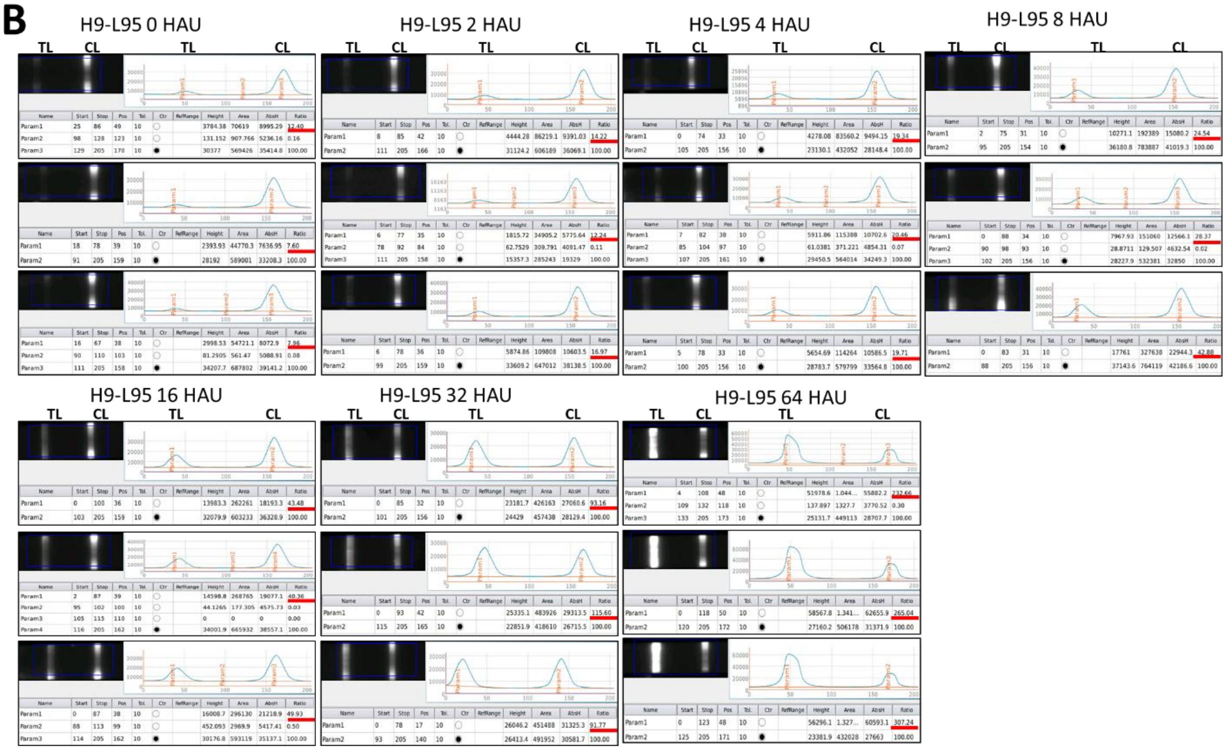
B
H9-L95 0 HAU
H9-L95 2 HAU
H9-L95 4 HAU
H9-L95 8 HAU
H9-L95 16 HAU
H9-L95 32 HAU
H9-L95 64 HAU
TL
CL

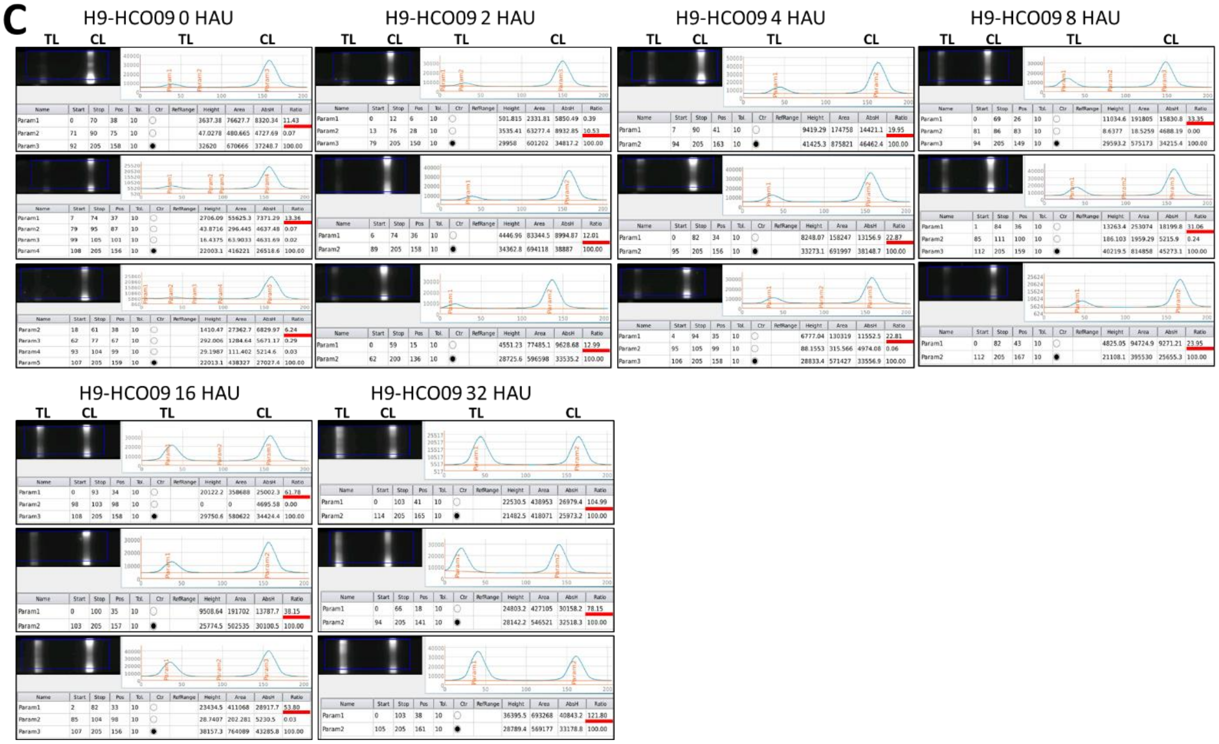
C
H9-HCO09 0 HAU
H9-HCO09 2 HAU
H9-HCO09 4 HAU
H9-HCO09 8 HAU
H9-HCO09 16 HAU
H9-HCO09 32 HAU
TL
CL

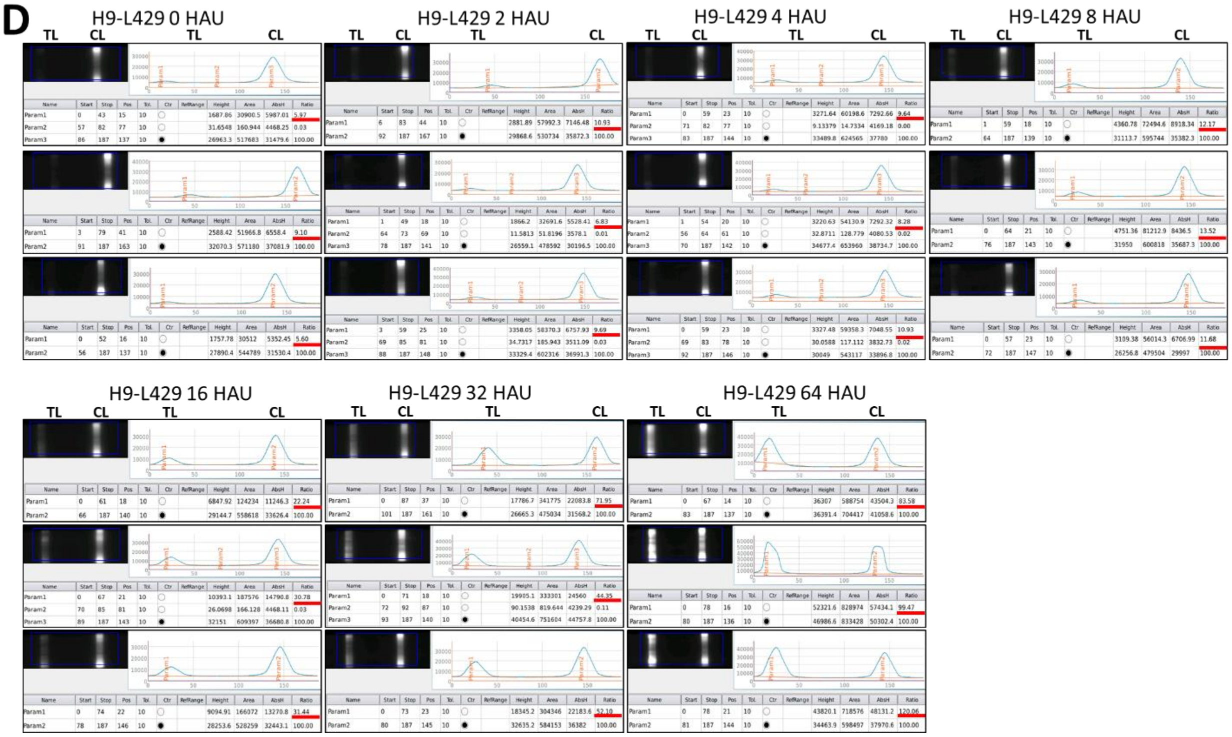

Fig.S11. The limit of detection of four H9N2 virus strains in Chungbuk. TL/CL is underlined with red color.

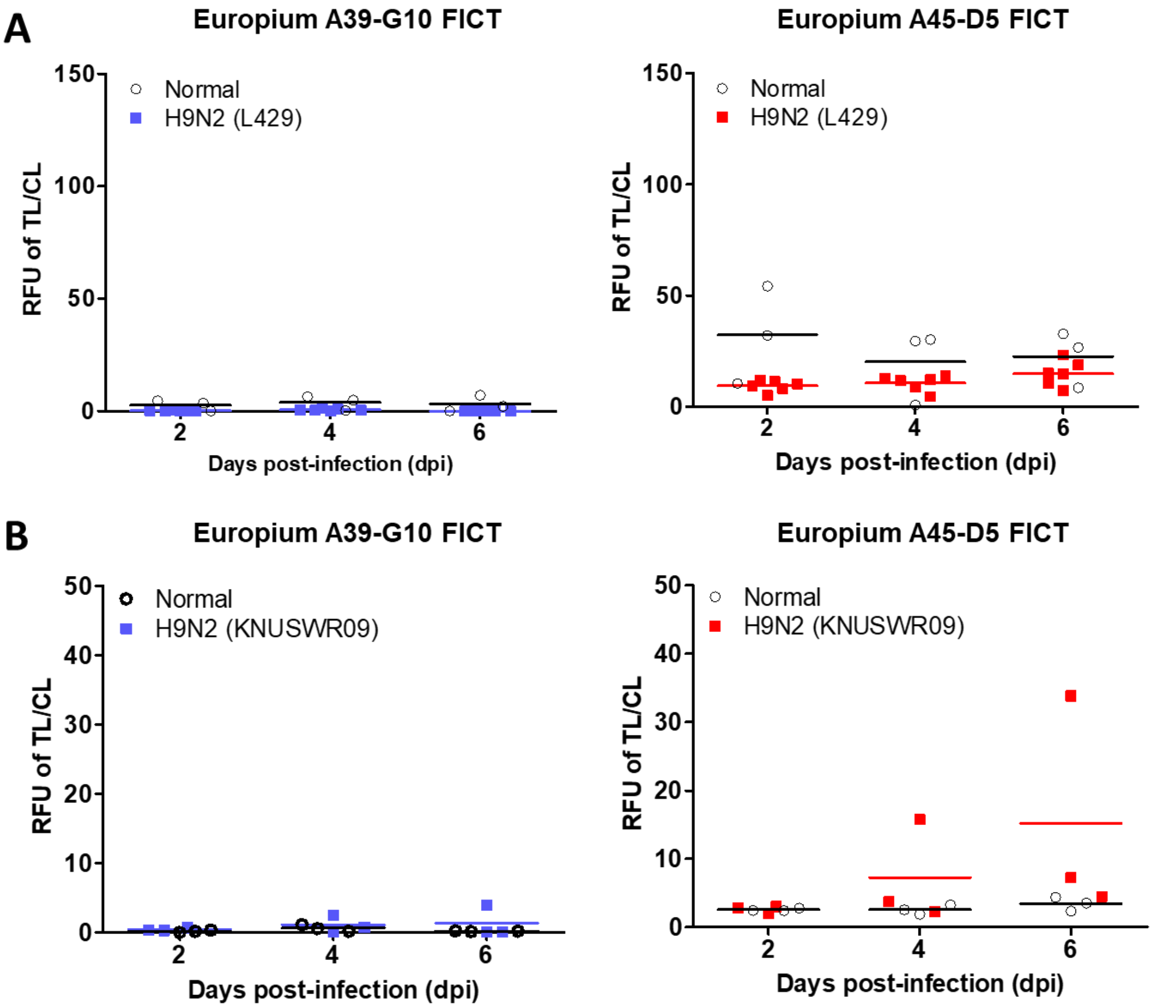

Fig.S12. Analysis of Europium A45-D5 FICT and Europium A39-G10 FICT using cloacal samples from SPF chickens at different day post inoculation with two H9N2 strains. The cut-off value used for detection of H9N2 virus was applied to determine the present of the virus in fecal samples. (A) A/Chicken/Korea/LPM429/2016 (H9N2) strain, (B) A/chicken/Korea/KNUSWR09/2009(H9N2) strain

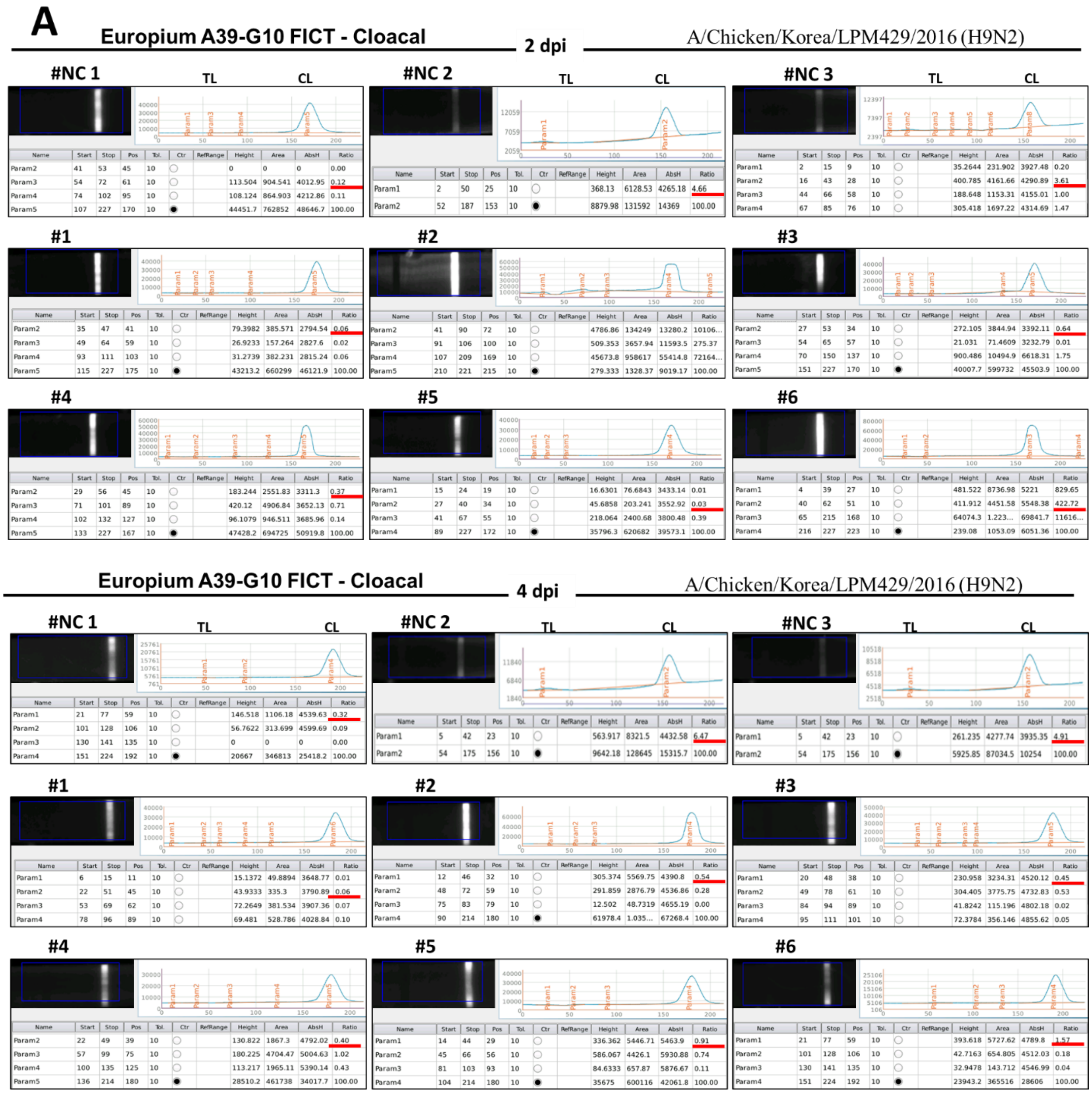
A
Europium A39-G10 FICT - Cloacal
2 dpi
A/Chicken/Korea/LPM429/2016 (H9N2)
#NC 1
#NC 2
#NC 3
TL
CL
#1
#2
#3
#4
#5
#6
Europium A39-G10 FICT - Cloacal
4 dpi
A/Chicken/Korea/LPM429/2016 (H9N2)
#NC 1
#NC 2
#NC 3
TL
CL
#1
#2
#3
#4
#5
#6

Europium A39-G10 FICT - Cloacal | **6 dpi** | A/Chicken/Korea/LPM429/2016 (H9N2)

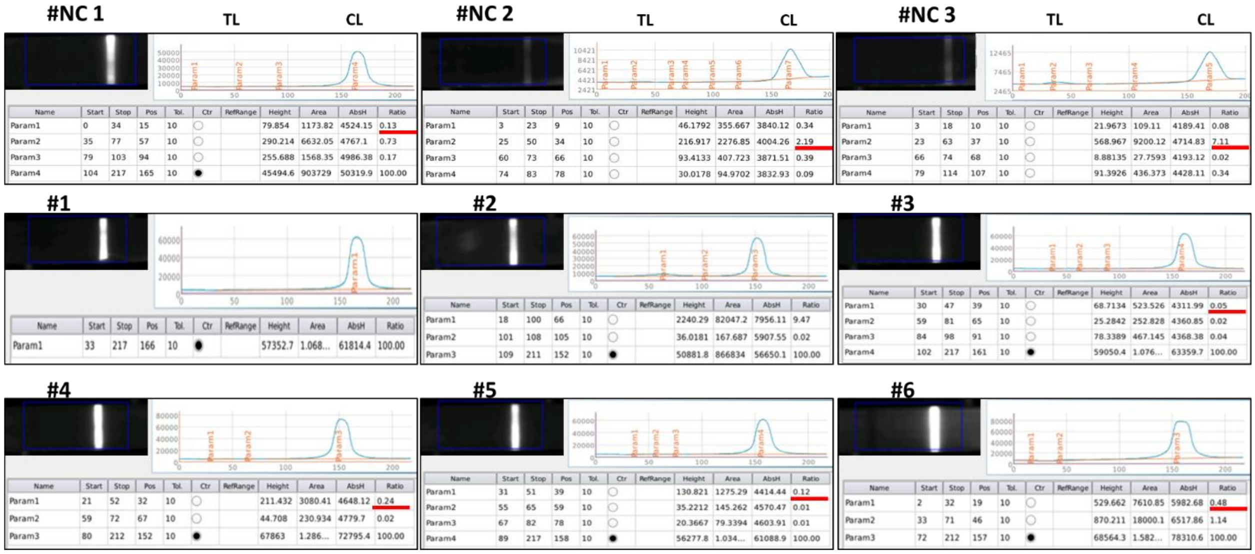

Europium A45-D5 FICT - Cloacal | **2 dpi** | A/Chicken/Korea/LPM429/2016 (H9N2)

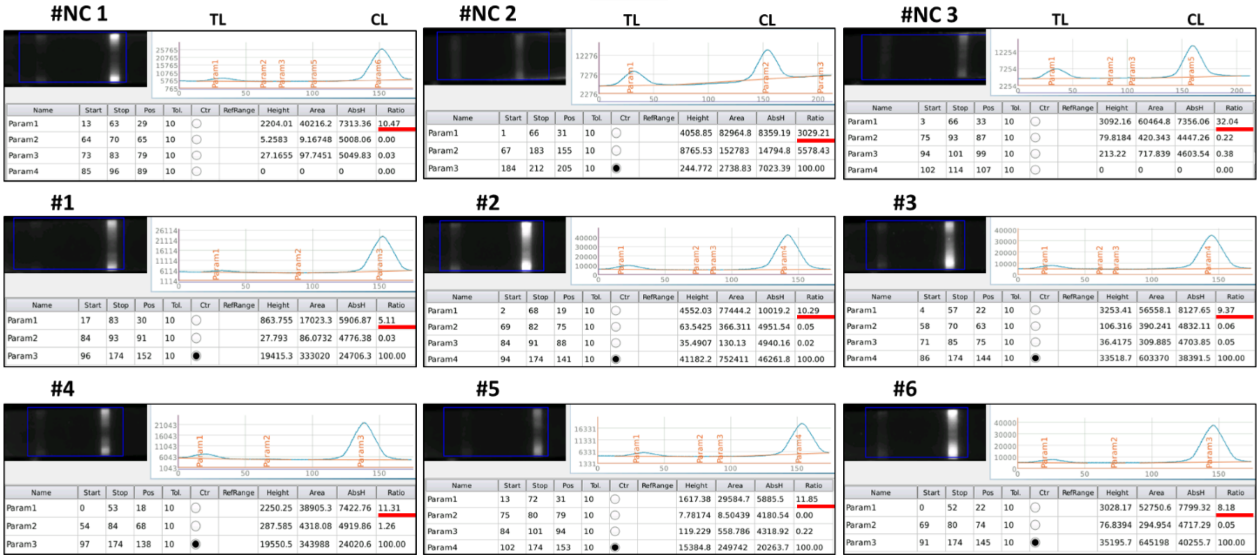

Europium A45-D5 FICT - Cloacal — 4 dpi — A/Chicken/Korea/LPM429/2016 (H9N2)

#NC 1

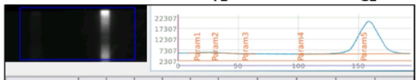

Name	Start	Stop	Pos	Tol.	Ctr	RefRange	Height	Area	AbsH	Ratio
Param2	26	53	33	10	○		290.004	2120.06	6169.73	0.69
Param3	54	65	58	10	○		52.7183	269.654	5503.47	0.09
Param4	99	110	104	10	○		36.8613	101.798	5372.5	0.03
Param5	111	193	157	10	●		16684.7	305970	22105.7	100.00

#NC 2

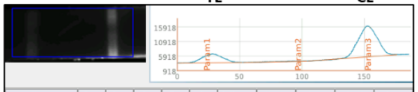

Name	Start	Stop	Pos	Tol.	Ctr	RefRange	Height	Area	AbsH	Ratio
Param1	1	52	26	10	○		3293.11	58081	6852.07	30.20
Param2	53	104	99	10	○		96.6719	2052.33	4172.88	1.07
Param3	105	176	155	10	●		12262.9	192297	17897.4	100.00

#NC 3

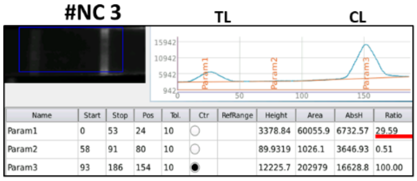

Name	Start	Stop	Pos	Tol.	Ctr	RefRange	Height	Area	AbsH	Ratio
Param1	0	53	24	10	○		3378.84	60055.9	6732.57	29.59
Param2	58	91	80	10	○		89.9319	1026.1	3646.93	0.51
Param3	93	186	154	10	●		12225.7	202979	16628.8	100.00

#1

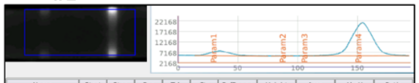

Name	Start	Stop	Pos	Tol.	Ctr	RefRange	Height	Area	AbsH	Ratio
Param1	14	75	32	10	○		2334.44	44923.7	7816.78	13.79
Param2	78	96	90	10	○		42.355	411.703	5426.67	0.13
Param3	102	113	108	10	○		37.3306	110.728	5468.24	0.03
Param4	114	193	153	10	●		17515.8	325828	22986	100.00

#2

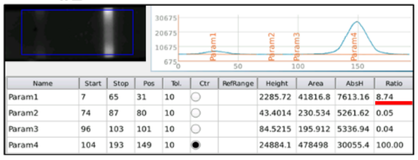

Name	Start	Stop	Pos	Tol.	Ctr	RefRange	Height	Area	AbsH	Ratio
Param1	7	65	31	10	○		2285.72	41816.8	7613.16	8.74
Param2	74	87	80	10	○		43.4014	230.534	5261.62	0.05
Param3	96	103	101	10	○		84.5215	195.912	5336.94	0.04
Param4	104	193	149	10	●		24884.1	478498	30055.4	100.00

#3

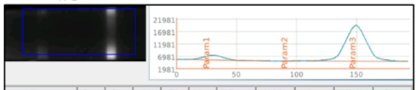

Name	Start	Stop	Pos	Tol.	Ctr	RefRange	Height	Area	AbsH	Ratio
Param1	10	78	27	10	○		2069.56	36195.1	7568.53	12.13
Param2	89	103	92	10	○		10.2812	43.5894	5056.71	0.01
Param3	108	193	149	10	●		16271.8	298321	21244.6	100.00

#4

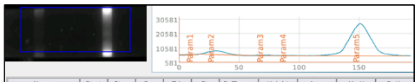

Name	Start	Stop	Pos	Tol.	Ctr	RefRange	Height	Area	AbsH	Ratio
Param1	0	16	11	10	○		362.548	1735.98	6011.63	0.38
Param2	17	62	29	10	○		3176	53932.9	8770.72	11.72
Param3	63	81	70	10	○		519.936	2972.69	5672.12	0.65
Param4	82	108	89	10	○		165.147	1604.64	5310.7	0.35

#5

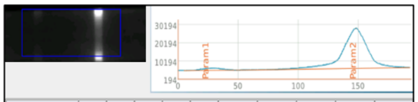

Name	Start	Stop	Pos	Tol.	Ctr	RefRange	Height	Area	AbsH	Ratio
Param1	5	47	25	10	○		1491.74	23466.2	6430.99	4.51
Param2	48	193	148	10	●		24424.1	519954	30483	100.00

#6

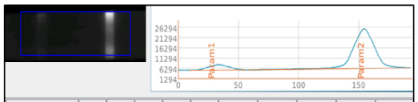

Name	Start	Stop	Pos	Tol.	Ctr	RefRange	Height	Area	AbsH	Ratio
Param1	9	56	30	10	○		2937.48	50101.5	8323.86	12.85
Param2	58	193	154	10	●		21398.7	389931	27603.3	100.00

Europium A45-D5 FICT - Cloacal — 6 dpi — A/Chicken/Korea/LPM429/2016 (H9N2)

#NC 1

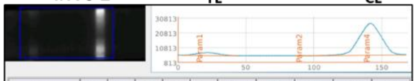

Name	Start	Stop	Pos	Tol.	Ctr	RefRange	Height	Area	AbsH	Ratio
Param1	3	44	18	10	○		2231.01	36080.6	7627.84	8.52
Param2	63	85	81	10	○		97.2598	513.191	5297.43	0.12
Param3	86	97	91	10	○		0	0	0	0.00
Param4	98	168	141	10	●		24249.6	423718	29626.8	100.00

#NC 2

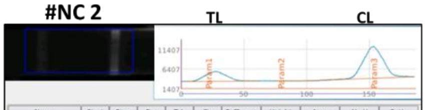

Name	Start	Stop	Pos	Tol.	Ctr	RefRange	Height	Area	AbsH	Ratio
Param1	3	62	24	10	○		2735.47	50006	5953.36	32.74
Param2	74	93	82	10	○		60.8787	558.13	3375.87	0.37
Param3	94	186	156	10	●		9264.4	152727	13273	100.00

#NC 3

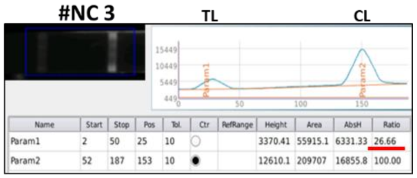

Name	Start	Stop	Pos	Tol.	Ctr	RefRange	Height	Area	AbsH	Ratio
Param1	2	50	25	10	○		3370.41	55915.1	6331.33	26.66
Param2	52	187	153	10	●		12610.1	209707	16855.8	100.00

#1

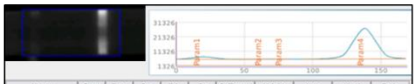

Name	Start	Stop	Pos	Tol.	Ctr	RefRange	Height	Area	AbsH	Ratio
Param1	2	45	17	10	○		1794.29	30508.1	7509.69	7.20
Param2	46	68	62	10	○		79.0513	491.687	5760.19	0.12
Param3	69	84	77	10	○		49.5786	172.433	5763.5	0.04
Param4	92	168	137	10	●		23736.2	423721	29544.3	100.00

#2

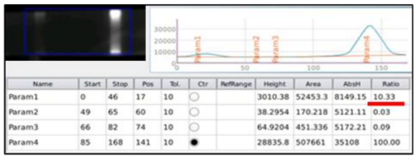

Name	Start	Stop	Pos	Tol.	Ctr	RefRange	Height	Area	AbsH	Ratio
Param1	0	46	17	10	○		3010.38	52453.3	8149.15	10.33
Param2	49	65	60	10	○		38.2954	170.218	5121.11	0.03
Param3	66	82	74	10	○		64.9204	451.336	5172.21	0.09
Param4	85	168	141	10	●		28835.8	507661	35108	100.00

#3

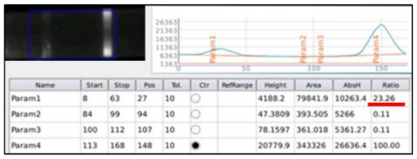

Name	Start	Stop	Pos	Tol.	Ctr	RefRange	Height	Area	AbsH	Ratio
Param1	8	63	27	10	○		4188.2	79841.9	10263.4	23.26
Param2	84	99	94	10	○		47.3809	393.505	5266	0.11
Param3	100	112	107	10	○		78.1597	361.018	5361.27	0.11
Param4	113	168	148	10	●		20779.9	343326	26636.4	100.00

#4

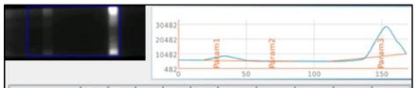

Name	Start	Stop	Pos	Tol.	Ctr	RefRange	Height	Area	AbsH	Ratio
Param1	18	63	30	10	○		3305.67	57326.5	9132.11	14.66
Param2	65	87	71	10	○		30.1582	138.803	5399.94	0.04
Param3	111	168	151	10	●		24634.9	391122	31120.9	100.00

#5

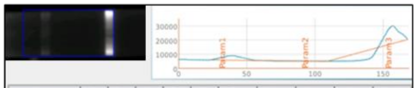

Name	Start	Stop	Pos	Tol.	Ctr	RefRange	Height	Area	AbsH	Ratio
Param1	24	89	34	10	○		3607.21	64077	9153.77	18.77
Param2	90	108	95	10	○		76.9102	653.58	5111.03	0.19
Param3	109	168	156	10	●		23762.9	341409	32747	100.00

#6

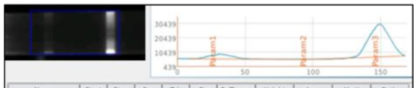

Name	Start	Stop	Pos	Tol.	Ctr	RefRange	Height	Area	AbsH	Ratio
Param1	9	84	28	10	○		3541.59	65810.7	9124.94	15.01
Param2	85	101	95	10	○		88.085	586.908	5415.44	0.13
Param3	102	168	148	10	●		25824.7	438325	32103	100.00

B Europium A39-G10 FICT - Feces — 2 dpi — A/Chicken/Korea/LPM429/2016 (H9N2)

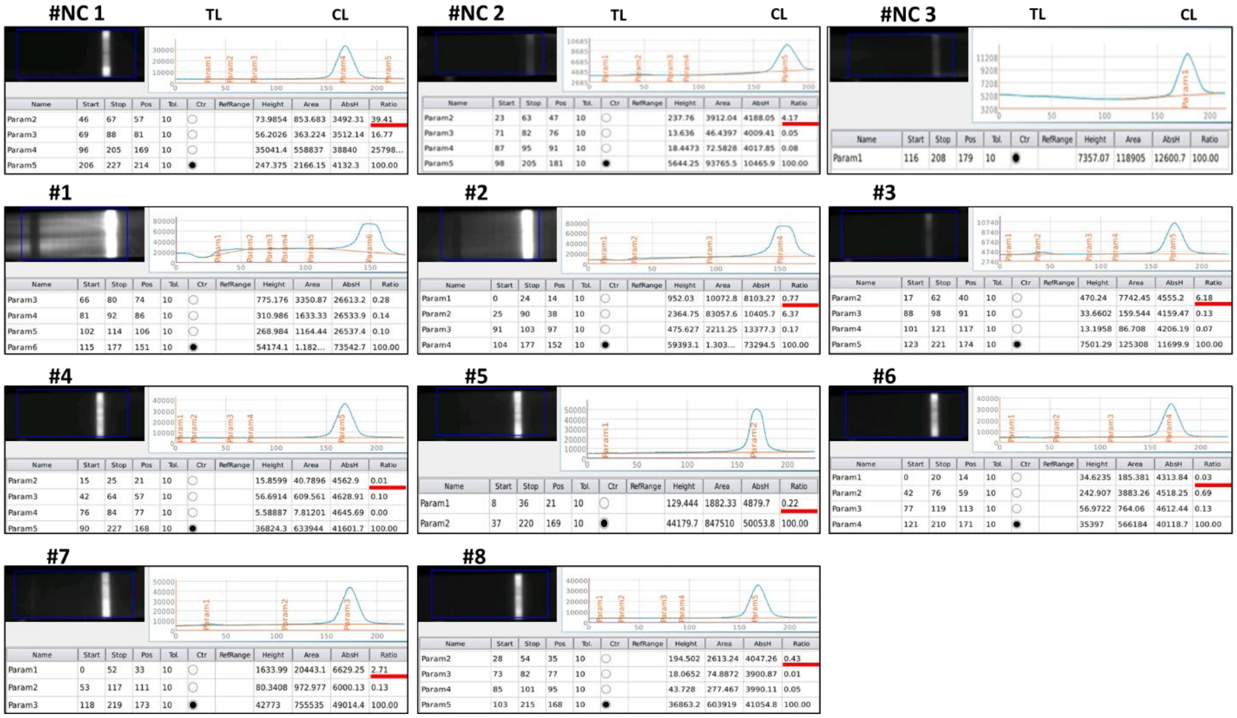

#NC 1 (TL, CL)

Name	Start	Stop	Pos	Tol.	Ctr	RefRange	Height	Area	AbsH	Ratio
Param2	46	67	57	10	○		73.9854	853.683	3492.31	39.41
Param3	69	88	81	10	○		56.2026	363.224	3512.14	16.77
Param4	96	205	169	10	○		35041.4	558837	38840	25798...
Param5	206	227	214	10	●		247.375	2166.15	4132.3	100.00

#NC 2 (TL, CL)

Name	Start	Stop	Pos	Tol.	Ctr	RefRange	Height	Area	AbsH	Ratio
Param2	23	63	47	10	○		237.76	3912.04	4188.05	4.17
Param3	71	82	76	10	○		13.636	46.4397	4009.41	0.05
Param4	87	95	91	10	○		18.4473	72.5828	4017.85	0.08
Param5	98	205	181	10	●		5644.25	93765.5	10465.9	100.00

#NC 3 (TL, CL)

Name	Start	Stop	Pos	Tol.	Ctr	RefRange	Height	Area	AbsH	Ratio
Param1	116	208	179	10	●		7357.07	118905	12600.7	100.00

#1

Name	Start	Stop	Pos	Tol.	Ctr	RefRange	Height	Area	AbsH	Ratio
Param3	66	80	74	10	○		775.176	3350.87	26613.2	0.28
Param4	81	92	86	10	○		310.986	1633.33	26533.9	0.14
Param5	102	114	106	10	○		268.984	1164.44	26537.4	0.10
Param6	115	177	151	10	●		54174.1	1.182...	73542.7	100.00

#2

Name	Start	Stop	Pos	Tol.	Ctr	RefRange	Height	Area	AbsH	Ratio
Param1	0	24	14	10	○		952.03	10072.8	8103.27	0.77
Param2	25	90	38	10	○		2364.75	83057.6	10405.7	6.37
Param3	91	103	97	10	○		475.627	2211.25	13377.3	0.17
Param4	104	177	152	10	●		59393.1	1.303...	73294.5	100.00

#3

Name	Start	Stop	Pos	Tol.	Ctr	RefRange	Height	Area	AbsH	Ratio
Param2	17	62	40	10	○		470.24	7742.45	4555.2	6.18
Param3	88	98	91	10	○		33.6602	159.544	4159.47	0.13
Param4	101	121	117	10	○		13.1958	86.708	4206.19	0.07
Param5	123	221	174	10	●		7501.29	125308	11699.9	100.00

#4

Name	Start	Stop	Pos	Tol.	Ctr	RefRange	Height	Area	AbsH	Ratio
Param2	15	25	21	10	○		15.8599	40.7896	4562.9	0.01
Param3	42	64	57	10	○		56.6914	609.561	4628.91	0.10
Param4	76	84	77	10	○		5.58887	7.81201	4645.69	0.00
Param5	90	227	168	10	●		36824.3	633944	41601.7	100.00

#5

Name	Start	Stop	Pos	Tol.	Ctr	RefRange	Height	Area	AbsH	Ratio
Param1	8	36	21	10	○		129.444	1882.33	4879.7	0.22
Param2	37	220	169	10	●		44179.7	847510	50053.8	100.00

#6

Name	Start	Stop	Pos	Tol.	Ctr	RefRange	Height	Area	AbsH	Ratio
Param1	0	20	14	10	○		34.6235	185.381	4313.84	0.03
Param2	42	76	59	10	○		242.907	3883.26	4518.25	0.69
Param3	77	119	113	10	○		56.9722	764.06	4612.44	0.13
Param4	121	210	171	10	●		35397	566184	40118.7	100.00

#7

Name	Start	Stop	Pos	Tol.	Ctr	RefRange	Height	Area	AbsH	Ratio
Param1	0	52	33	10	○		1633.99	20443.1	6629.25	2.71
Param2	53	117	111	10	○		80.3408	972.977	6000.13	0.13
Param3	118	219	173	10	●		42773	755535	49014.4	100.00

#8

Name	Start	Stop	Pos	Tol.	Ctr	RefRange	Height	Area	AbsH	Ratio
Param2	28	54	35	10	○		194.502	2613.24	4047.26	0.43
Param3	73	82	77	10	○		18.0652	74.8872	3900.87	0.01
Param4	85	101	95	10	○		43.728	277.467	3990.11	0.05
Param5	103	215	168	10	●		36863.2	603919	41054.8	100.00

Europium A39-G10 FICT - Feces — 4 dpi — A/Chicken/Korea/LPM429/2016 (H9N2)

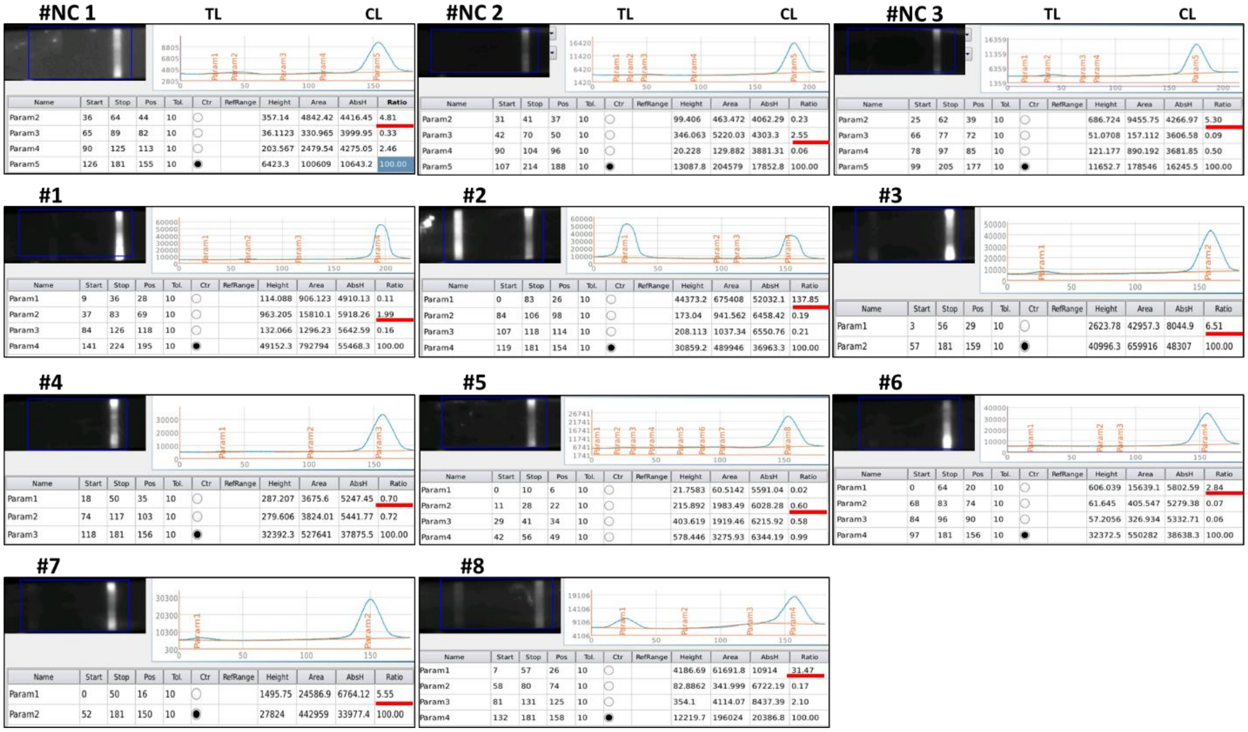

#NC 1 (TL, CL)

Name	Start	Stop	Pos	Tol.	Ctr	RefRange	Height	Area	AbsH	Ratio
Param2	36	64	44	10	○		357.14	4842.42	4416.45	4.81
Param3	65	89	82	10	○		36.1123	330.965	3999.95	0.33
Param4	90	125	113	10	○		203.567	2479.54	4275.05	2.46
Param5	126	181	155	10	●		6423.3	100609	10643.2	100.00

#NC 2 (TL, CL)

Name	Start	Stop	Pos	Tol.	Ctr	RefRange	Height	Area	AbsH	Ratio
Param2	31	41	37	10	○		99.406	463.472	4062.29	0.23
Param3	42	70	50	10	○		346.063	5220.03	4303.3	2.55
Param4	90	104	96	10	○		20.228	129.882	3881.31	0.06
Param5	107	214	188	10	●		13087.8	204579	17852.8	100.00

#NC 3 (TL, CL)

Name	Start	Stop	Pos	Tol.	Ctr	RefRange	Height	Area	AbsH	Ratio
Param2	25	62	39	10	○		686.724	9455.75	4266.97	5.30
Param3	66	77	72	10	○		51.0708	157.112	3606.58	0.09
Param4	78	97	85	10	○		121.177	890.192	3681.85	0.50
Param5	99	205	177	10	●		11652.7	178546	16245.5	100.00

#1

Name	Start	Stop	Pos	Tol.	Ctr	RefRange	Height	Area	AbsH	Ratio
Param1	9	36	28	10	○		114.088	906.123	4910.13	0.11
Param2	37	83	69	10	○		963.205	15810.1	5918.26	1.99
Param3	84	126	118	10	○		132.066	1296.23	5642.59	0.16
Param4	141	224	195	10	●		49152.3	792794	55468.3	100.00

#2

Name	Start	Stop	Pos	Tol.	Ctr	RefRange	Height	Area	AbsH	Ratio
Param1	0	83	26	10	○		44373.2	675408	52032.1	137.85
Param2	84	106	98	10	○		173.04	941.562	6458.42	0.19
Param3	107	118	114	10	○		208.113	1037.34	6550.76	0.21
Param4	119	181	154	10	●		30859.2	489946	36963.3	100.00

#3

Name	Start	Stop	Pos	Tol.	Ctr	RefRange	Height	Area	AbsH	Ratio
Param1	3	56	29	10	○		2623.78	42957.3	8044.9	6.51
Param2	57	181	159	10	●		40996.3	659916	48307	100.00

#4

Name	Start	Stop	Pos	Tol.	Ctr	RefRange	Height	Area	AbsH	Ratio
Param1	18	50	35	10	○		287.207	3675.6	5247.45	0.70
Param2	74	117	103	10	○		279.606	3824.01	5441.77	0.72
Param3	118	181	156	10	●		32392.3	527641	37875.5	100.00

#5

Name	Start	Stop	Pos	Tol.	Ctr	RefRange	Height	Area	AbsH	Ratio
Param1	0	10	6	10	○		21.7583	60.5142	5591.04	0.02
Param2	11	28	22	10	○		215.892	1983.49	6028.28	0.60
Param3	29	41	34	10	○		403.619	1919.46	6215.92	0.58
Param4	42	56	49	10	○		578.446	3275.93	6344.19	0.99

#6

Name	Start	Stop	Pos	Tol.	Ctr	RefRange	Height	Area	AbsH	Ratio
Param1	0	64	20	10	○		606.039	15639.1	5802.59	2.84
Param2	68	83	74	10	○		61.645	405.547	5279.38	0.07
Param3	84	96	90	10	○		57.2056	326.934	5332.71	0.06
Param4	97	181	156	10	●		32372.5	550282	38638.3	100.00

#7

Name	Start	Stop	Pos	Tol.	Ctr	RefRange	Height	Area	AbsH	Ratio
Param1	0	50	16	10	○		1495.75	24586.9	6764.12	5.55
Param2	52	181	150	10	●		27824	442959	33977.4	100.00

#8

Name	Start	Stop	Pos	Tol.	Ctr	RefRange	Height	Area	AbsH	Ratio
Param1	7	57	26	10	○		4186.69	61691.8	10914	31.47
Param2	58	80	74	10	○		82.8862	341.999	6722.19	0.17
Param3	81	131	125	10	○		354.1	4114.07	8437.39	2.10
Param4	132	181	158	10	●		12219.7	196024	20386.8	100.00

Europium A39-G10 FICT - Feces — 6 dpi — A/Chicken/Korea/LPM429/2016 (H9N2)

#NC 1 TL CL

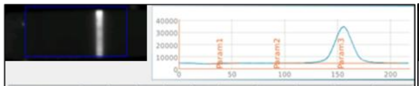

Name	Start	Stop	Pos	Tol.	Ctr	RefRange	Height	Area	AbsH	Ratio
Param1	27	57	41	10	○		324.233	4997.3	4496.77	0.82
Param2	89	108	95	10	○		15.2388	87.9009	4495.29	0.01
Param3	109	210	156	10	●		35486	609101	40008.8	100.00

#NC 2 TL CL

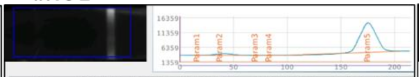

Name	Start	Stop	Pos	Tol.	Ctr	RefRange	Height	Area	AbsH	Ratio
Param1	13	24	18	10	○		31.5496	163.751	3634.22	0.09
Param2	25	62	39	10	○		686.724	9455.75	4266.97	5.30
Param3	66	77	72	10	○		51.0708	157.112	3606.58	0.09
Param4	78	97	85	10	○		121.177	890.192	3681.85	0.50

#NC 3 TL CL

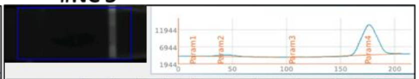

Name	Start	Stop	Pos	Tol.	Ctr	RefRange	Height	Area	AbsH	Ratio
Param1	9	26	16	10	○		46.0752	341.195	4210.43	0.23
Param2	29	93	42	10	○		471.599	8094.36	4561.73	5.42
Param3	99	117	108	10	○		110.026	763.094	3972.29	0.51
Param4	119	206	178	10	●		10610	149472	15165	100.00

#1

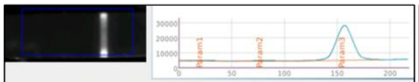

Name	Start	Stop	Pos	Tol.	Ctr	RefRange	Height	Area	AbsH	Ratio
Param1	0	47	22	10	○		469.46	9357.83	4956.04	2.16
Param2	50	105	79	10	○		458.754	9015.84	4868.76	2.09
Param3	107	192	157	10	●		28190.8	432261	33164.4	100.00

#2

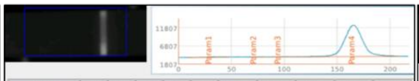

Name	Start	Stop	Pos	Tol.	Ctr	RefRange	Height	Area	AbsH	Ratio
Param1	9	48	31	10	○		111.38	1690.65	3751.93	0.98
Param2	69	83	73	10	○		35.3579	260.432	3735.87	0.15
Param3	86	109	96	10	○		102.627	1087.23	3839.27	0.63
Param4	111	209	166	10	●		10311	172042	14158.1	100.00

#3

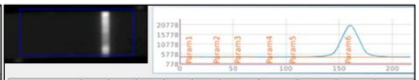

Name	Start	Stop	Pos	Tol.	Ctr	RefRange	Height	Area	AbsH	Ratio
Param3	43	72	57	10	○		115.001	1351.58	4166.13	0.42
Param4	83	94	87	10	○		23.989	84.0581	4048.02	0.03
Param5	104	115	109	10	○		28.4241	108.688	4072.31	0.03
Param6	116	212	161	10	●		18785.4	323119	22823.6	100.00

#4

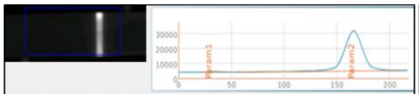

Name	Start	Stop	Pos	Tol.	Ctr	RefRange	Height	Area	AbsH	Ratio
Param1	20	50	31	10	○		190.098	2716.05	4400.22	0.50
Param2	54	212	166	10	●		31581.2	545919	36505.2	100.00

#5

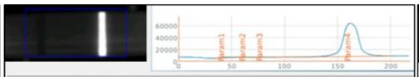

Name	Start	Stop	Pos	Tol.	Ctr	RefRange	Height	Area	AbsH	Ratio
Param1	32	55	43	10	○		718.338	7085.05	6083.65	0.74
Param2	56	69	63	10	○		38.311	157.54	6286.07	0.02
Param3	70	83	79	10	○		92.4565	455.885	6339.3	0.05
Param4	85	203	162	10	●		55817.1	956184	63508.1	100.00

#6

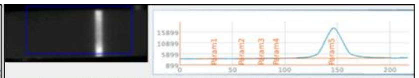

Name	Start	Stop	Pos	Tol.	Ctr	RefRange	Height	Area	AbsH	Ratio
Param2	53	65	61	10	○		40.0081	174.784	3825.78	0.06
Param3	66	86	80	10	○		70.8228	637.16	3919.41	0.23
Param4	88	99	94	10	○		17.8958	45.8682	3936.46	0.02
Param5	102	201	147	10	●		15738.8	282473	19721.4	100.00

#7

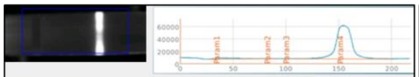

Name	Start	Stop	Pos	Tol.	Ctr	RefRange	Height	Area	AbsH	Ratio
Param1	27	79	37	10	○		1617.18	35774	9072.19	3.86
Param2	81	92	85	10	○		70.2832	255.701	7951.24	0.03
Param3	100	106	103	10	○		58.9961	203.999	7884	0.02
Param4	108	202	154	10	●		53328.2	927173	61024.2	100.00

#8

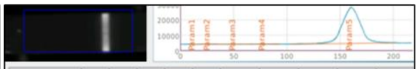

Name	Start	Stop	Pos	Tol.	Ctr	RefRange	Height	Area	AbsH	Ratio
Param2	19	45	27	10	○		261.406	3395.12	4184.53	0.74
Param3	46	57	51	10	○		52.8923	202.15	4012.35	0.04
Param4	58	94	79	10	○		77.7932	1292.73	4142.6	0.28
Param5	104	202	161	10	●		27319.5	458356	31929.5	100.00

Europium A45-D5 FICT - Feces — 2 dpi — A/Chicken/Korea/LPM429/2016 (H9N2)

#NC 1 TL CL

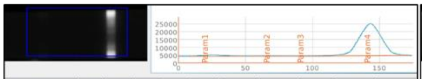

Name	Start	Stop	Pos	Tol.	Ctr	RefRange	Height	Area	AbsH	Ratio
Param1	8	51	22	10	○		755.489	13391.6	4947.91	3.31
Param2	54	87	68	10	○		114.651	1748.72	4345.05	0.43
Param3	88	98	93	10	○		40.1748	182.216	4302.25	0.05
Param4	99	174	143	10	●		22927	403999	27394.2	100.00

#NC 2 TL CL

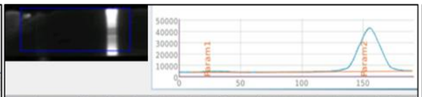

Name	Start	Stop	Pos	Tol.	Ctr	RefRange	Height	Area	AbsH	Ratio
Param1	9	59	25	10	○		1071.41	19987.8	4759.69	2.50
Param2	63	190	153	10	●		40559.4	800228	44947.7	100.00

#NC 3 TL CL

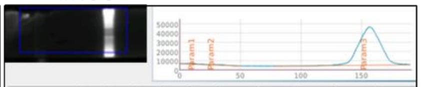

Name	Start	Stop	Pos	Tol.	Ctr	RefRange	Height	Area	AbsH	Ratio
Param1	0	21	12	10	○		1619.49	14958.4	7306.04	1.73
Param2	22	51	28	10	○		1001.92	12509.9	5966.19	1.45
Param3	78	190	154	10	●		43049.7	865316	47974.9	100.00

#1

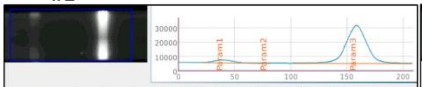

Name	Start	Stop	Pos	Tol.	Ctr	RefRange	Height	Area	AbsH	Ratio
Param1	21	76	39	10	○		2142.35	38474.2	7589.43	6.82
Param2	78	82	78	10	○		0	0	5154.36	0.00
Param3	103	208	158	10	●		29812.9	563773	34862.8	100.00

#2

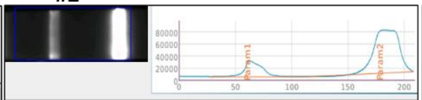

Name	Start	Stop	Pos	Tol.	Ctr	RefRange	Height	Area	AbsH	Ratio
Param1	27	111	63	10	○		26128.5	469879	31810.5	25.02
Param2	112	208	181	10	●		70580	1.878...	82341.6	100.00

#3

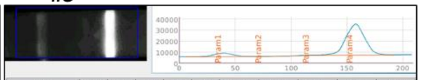

Name	Start	Stop	Pos	Tol.	Ctr	RefRange	Height	Area	AbsH	Ratio
Param1	6	66	37	10	○		3813.86	64301	9461.94	11.67
Param2	67	78	73	10	○		58.7334	388.851	5843.06	0.07
Param3	79	125	116	10	○		498.864	5340.43	7270.17	0.97
Param4	126	192	155	10	●		33427.9	551213	40530.8	100.00

#4

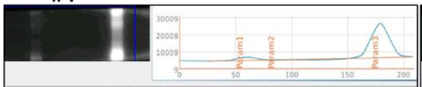

Name	Start	Stop	Pos	Tol.	Ctr	RefRange	Height	Area	AbsH	Ratio
Param1	25	78	56	10	○		2191.9	35771.5	7005.9	9.18
Param2	79	88	84	10	○		51.9565	180.344	5136.34	0.05
Param3	90	208	177	10	●		22018.5	389617	28476.6	100.00

#5

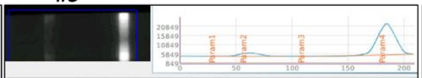

Name	Start	Stop	Pos	Tol.	Ctr	RefRange	Height	Area	AbsH	Ratio
Param1	23	38	31	10	○		43.0815	266.082	4479.06	0.08
Param2	39	95	59	10	○		2309.7	38352.9	6740.61	11.72
Param3	103	117	111	10	○		23.2881	72.1689	4441	0.02
Param4	121	208	183	10	●		19382.2	327373	24329.7	100.00

#6

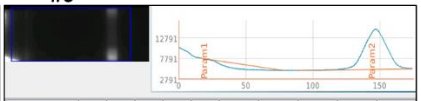

Name	Start	Stop	Pos	Tol.	Ctr	RefRange	Height	Area	AbsH	Ratio
Param1	15	79	21	10	○		1328.87	7439.09	8388.78	3.87
Param2	88	174	146	10	●		11016.2	192362	16044.7	100.00

#7

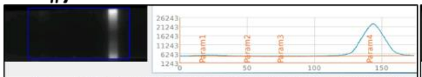

Name	Start	Stop	Pos	Tol.	Ctr	RefRange	Height	Area	AbsH	Ratio
Param1	7	41	19	10	○		756.567	10583.3	5670.01	2.99
Param2	42	69	52	10	○		88.6431	974.918	4917.57	0.28
Param3	70	89	77	10	○		153.026	1239.94	4952.78	0.35
Param4	90	174	143	10	●		19322.5	353676	24311.4	100.00

#8

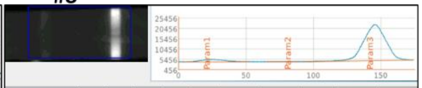

Name	Start	Stop	Pos	Tol.	Ctr	RefRange	Height	Area	AbsH	Ratio
Param1	3	53	23	10	○		1338.02	22906.2	5447.94	6.83
Param2	65	89	83	10	○		42.0435	334.087	4239.2	0.10
Param3	91	174	144	10	●		19648.3	335407	24259.6	100.00

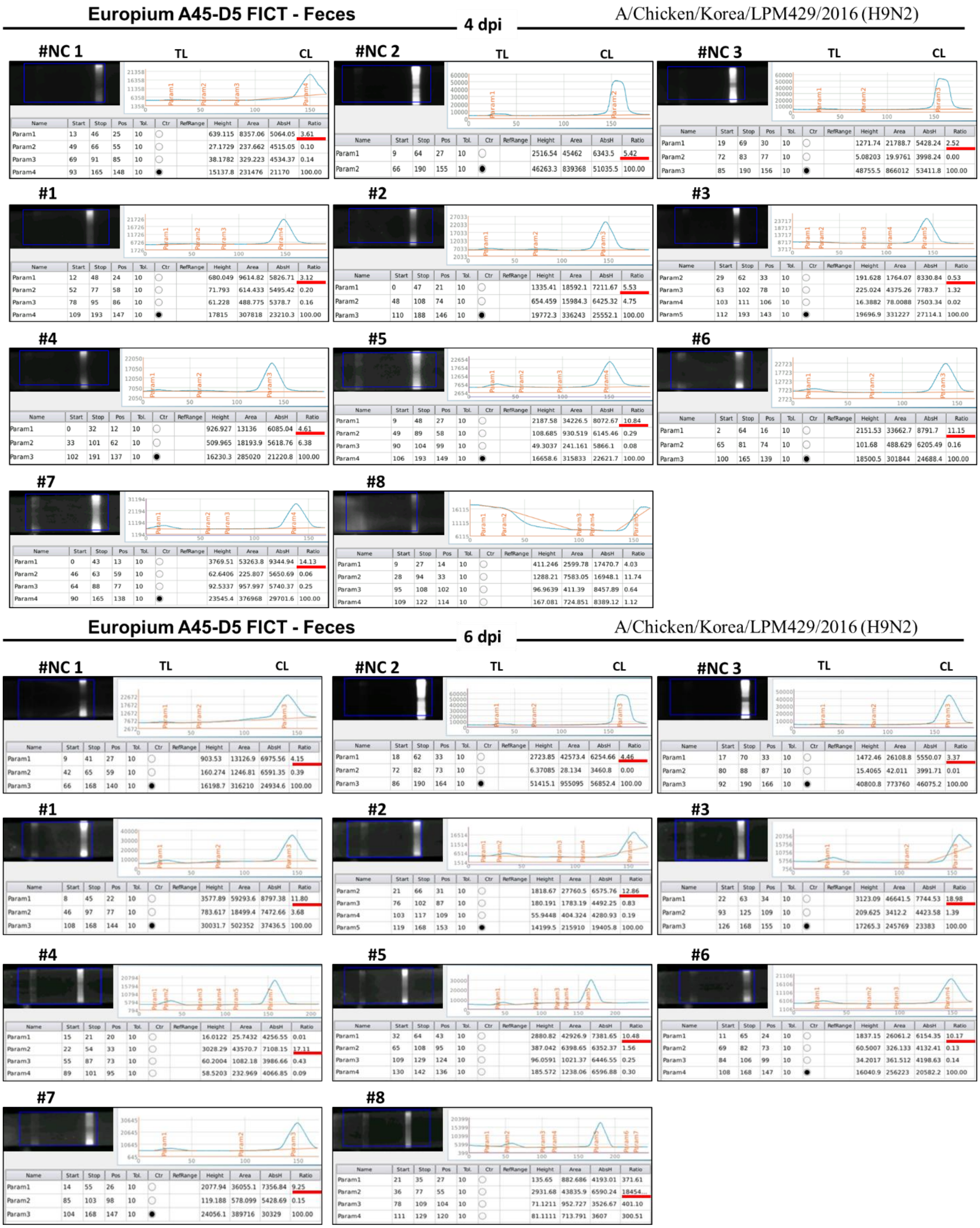

Fig.S13. Raw data of Europium A45-D5-FICT and Europium A39-G10 FICT using clinical specimens from SPF chickens at different day post inoculation with

A/Chicken/Korea/LPM429/2016 (H9N2) strain (A) Cloacal swabs, (B) feces samples. TL/CL is underlined with red color.

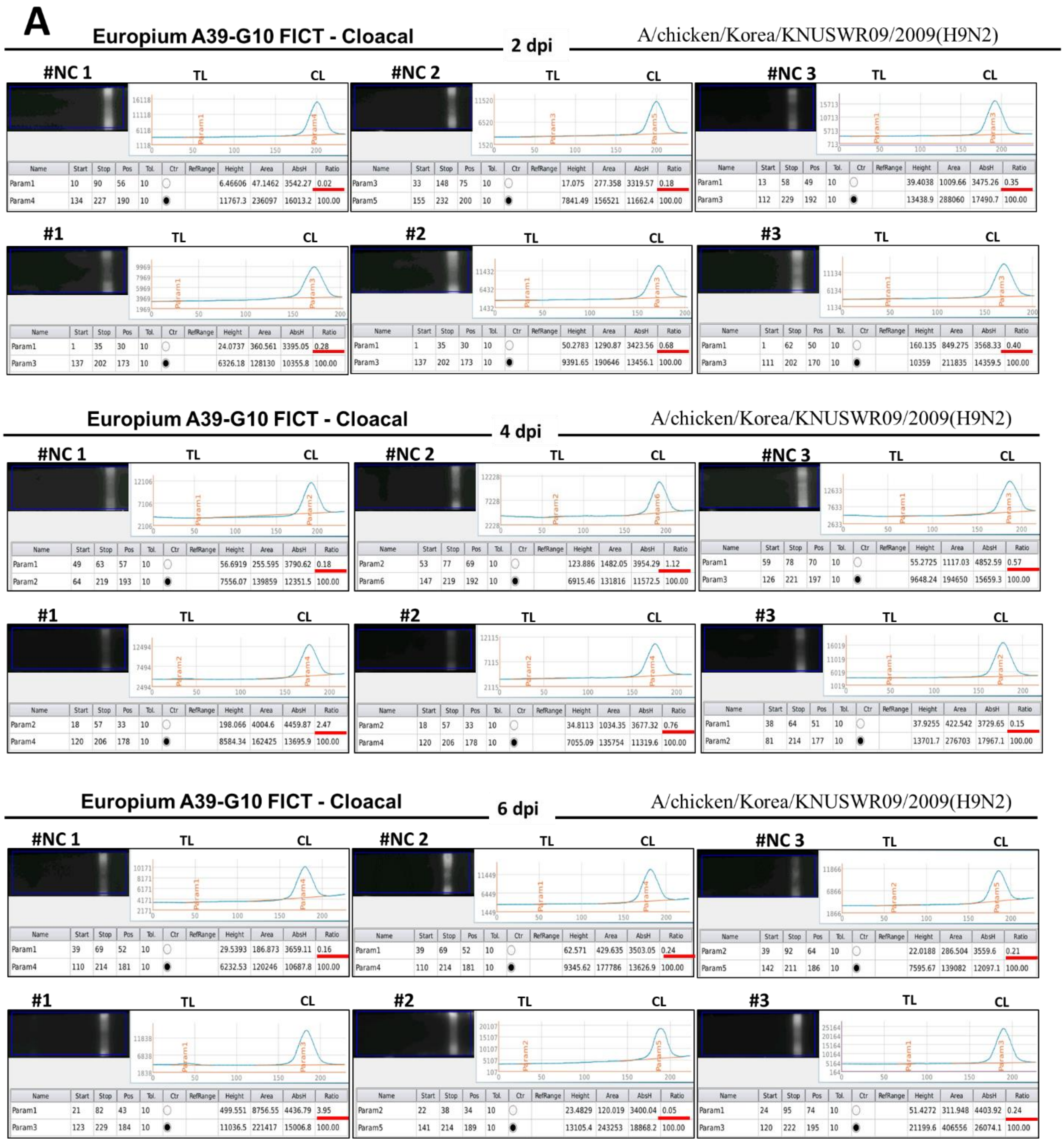
A
Europium A39-G10 FICT - Cloacal
2 dpi
A/chicken/Korea/KNUSWR09/2009(H9N2)
#NC 1 TL CL
Name Start Stop Pos Tol. Ctr RefRange Height Area AbsH Ratio
Param1 10 90 56 10 6.46606 47.1462 3542.27 0.02
Param4 134 227 190 10 11767.3 236097 16013.2 100.00
#NC 2 TL CL
Param3 33 148 75 10 17.075 277.358 3319.57 0.18
Param5 155 232 200 10 7841.49 156521 11662.4 100.00
#NC 3 TL CL
Param1 13 58 49 10 39.4038 1009.66 3475.26 0.35
Param3 112 229 192 10 13438.9 288060 17490.7 100.00
#1 TL CL
Param1 1 35 30 10 24.0737 360.561 3395.05 0.28
Param3 137 202 173 10 6326.18 128130 10355.8 100.00
#2 TL CL
Param1 1 35 30 10 50.2783 1290.87 3423.56 0.68
Param3 137 202 173 10 9391.65 190646 13456.1 100.00
#3 TL CL
Param1 1 62 50 10 160.135 849.275 3568.33 0.40
Param3 111 202 170 10 10359 211835 14359.5 100.00
Europium A39-G10 FICT - Cloacal
4 dpi
A/chicken/Korea/KNUSWR09/2009(H9N2)
#NC 1 TL CL
Param1 49 63 57 10 56.6919 255.595 3790.62 0.18
Param2 64 219 193 10 7556.07 139859 12351.5 100.00
#NC 2 TL CL
Param2 53 77 69 10 123.886 1482.05 3954.29 1.12
Param6 147 219 192 10 6915.46 131816 11572.5 100.00
#NC 3 TL CL
Param1 59 78 70 10 55.2725 1117.03 4852.59 0.57
Param3 126 221 197 10 9648.24 194650 15659.3 100.00
#1 TL CL
Param2 18 57 33 10 198.066 4004.6 4459.87 2.47
Param4 120 206 178 10 8584.34 162425 13695.9 100.00
#2 TL CL
Param2 18 57 33 10 34.8113 1034.35 3677.32 0.76
Param4 120 206 178 10 7055.09 135754 11319.6 100.00
#3 TL CL
Param1 38 64 51 10 37.9255 422.542 3729.65 0.15
Param2 81 214 177 10 13701.7 276703 17967.1 100.00
Europium A39-G10 FICT - Cloacal
6 dpi
A/chicken/Korea/KNUSWR09/2009(H9N2)
#NC 1 TL CL
Param1 39 69 52 10 29.5393 186.873 3659.11 0.16
Param4 110 214 181 10 6232.53 120246 10687.8 100.00
#NC 2 TL CL
Param1 39 69 52 10 62.571 429.635 3503.05 0.24
Param4 110 214 181 10 9345.62 177786 13626.9 100.00
#NC 3 TL CL
Param2 39 92 64 10 22.0188 286.504 3559.6 0.21
Param5 142 211 186 10 7595.67 139082 12097.1 100.00
#1 TL CL
Param1 21 82 43 10 499.551 8756.55 4436.79 3.95
Param3 123 229 184 10 11036.5 221417 15006.8 100.00
#2 TL CL
Param2 22 38 34 10 23.4829 120.019 3400.04 0.05
Param5 141 214 189 10 13105.4 243253 18868.2 100.00
#3 TL CL
Param1 24 95 74 10 51.4272 311.948 4403.92 0.24
Param3 120 222 195 10 21199.6 406556 26074.1 100.00

Europium A45-D5 FICT - Cloacal

2 dpi

A/chicken/Korea/KNUSWR09/2009(H9N2)

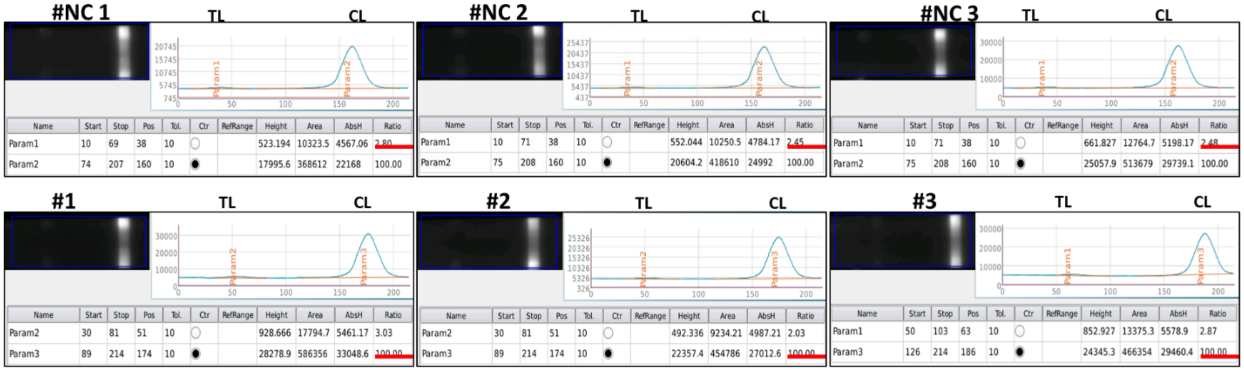

#NC 1 (TL / CL)

Name	Start	Stop	Pos	Tol.	Ctr	RefRange	Height	Area	AbsH	Ratio
Param1	10	69	38	10	○		523.194	10323.5	4567.06	2.80
Param2	74	207	160	10	●		17995.6	368612	22168	100.00

#NC 2 (TL / CL)

Name	Start	Stop	Pos	Tol.	Ctr	RefRange	Height	Area	AbsH	Ratio
Param1	10	71	38	10	○		552.044	10250.5	4784.17	2.45
Param2	75	208	160	10	●		20604.2	418610	24992	100.00

#NC 3 (TL / CL)

Name	Start	Stop	Pos	Tol.	Ctr	RefRange	Height	Area	AbsH	Ratio
Param1	10	71	38	10	○		661.827	12764.7	5198.17	2.48
Param2	75	208	160	10	●		25057.9	513679	29739.1	100.00

#1 (TL / CL)

Name	Start	Stop	Pos	Tol.	Ctr	RefRange	Height	Area	AbsH	Ratio
Param2	30	81	51	10	○		928.666	17794.7	5461.17	3.03
Param3	89	214	174	10	●		28278.9	586356	33048.6	100.00

#2 (TL / CL)

Name	Start	Stop	Pos	Tol.	Ctr	RefRange	Height	Area	AbsH	Ratio
Param2	30	81	51	10	○		492.336	9234.21	4987.21	2.03
Param3	89	214	174	10	●		22357.4	454786	27012.6	100.00

#3 (TL / CL)

Name	Start	Stop	Pos	Tol.	Ctr	RefRange	Height	Area	AbsH	Ratio
Param1	50	103	63	10	○		852.927	13375.3	5578.9	2.87
Param3	126	214	186	10	●		24345.3	466354	29460.4	100.00

Europium A45-D5 FICT - Cloacal

4 dpi

A/chicken/Korea/KNUSWR09/2009(H9N2)

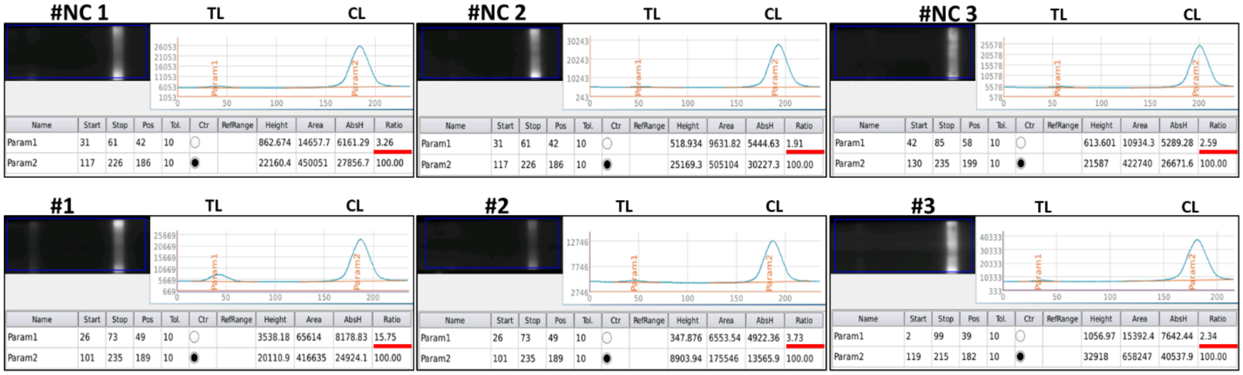

#NC 1 (TL / CL)

Name	Start	Stop	Pos	Tol.	Ctr	RefRange	Height	Area	AbsH	Ratio
Param1	31	61	42	10	○		862.674	14657.7	6161.29	3.26
Param2	117	226	186	10	●		22160.4	450051	27856.7	100.00

#NC 2 (TL / CL)

Name	Start	Stop	Pos	Tol.	Ctr	RefRange	Height	Area	AbsH	Ratio
Param1	31	61	42	10	○		518.934	9631.82	5444.63	1.91
Param2	117	226	186	10	●		25169.3	505104	30227.3	100.00

#NC 3 (TL / CL)

Name	Start	Stop	Pos	Tol.	Ctr	RefRange	Height	Area	AbsH	Ratio
Param1	42	85	58	10	○		613.601	10934.3	5289.28	2.59
Param2	130	235	199	10	●		21587	422740	26671.6	100.00

#1 (TL / CL)

Name	Start	Stop	Pos	Tol.	Ctr	RefRange	Height	Area	AbsH	Ratio
Param1	26	73	49	10	○		3538.18	65614	8178.83	15.75
Param2	101	235	189	10	●		20110.9	416635	24924.1	100.00

#2 (TL / CL)

Name	Start	Stop	Pos	Tol.	Ctr	RefRange	Height	Area	AbsH	Ratio
Param1	26	73	49	10	○		347.876	6553.54	4922.36	3.73
Param2	101	235	189	10	●		8903.94	175546	13565.9	100.00

#3 (TL / CL)

Name	Start	Stop	Pos	Tol.	Ctr	RefRange	Height	Area	AbsH	Ratio
Param1	2	99	39	10	○		1056.97	15392.4	7642.44	2.34
Param2	119	215	182	10	●		32918	658247	40537.9	100.00

Europium A45-D5 FICT - Cloacal

6 dpi

A/chicken/Korea/KNUSWR09/2009(H9N2)

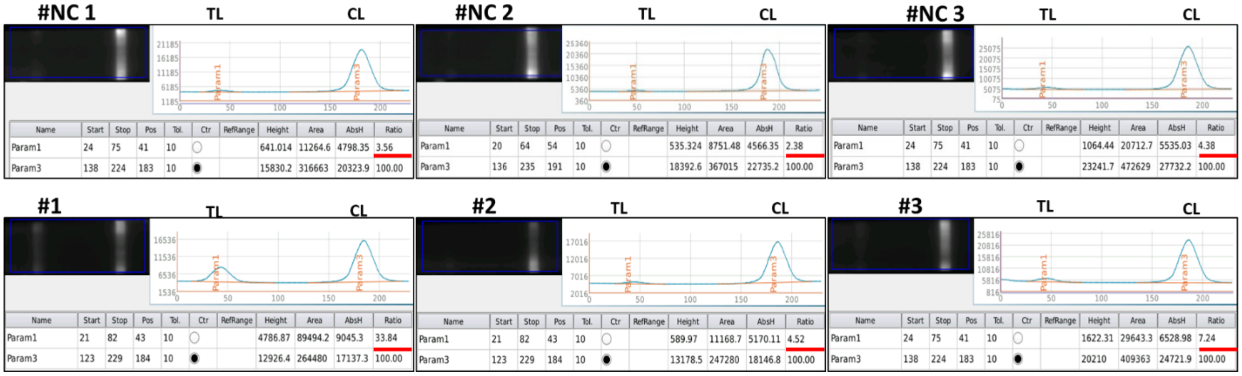

#NC 1 (TL / CL)

Name	Start	Stop	Pos	Tol.	Ctr	RefRange	Height	Area	AbsH	Ratio
Param1	24	75	41	10	○		641.014	11264.6	4798.35	3.56
Param3	138	224	183	10	●		15830.2	316663	20323.9	100.00

#NC 2 (TL / CL)

Name	Start	Stop	Pos	Tol.	Ctr	RefRange	Height	Area	AbsH	Ratio
Param1	20	64	54	10	○		535.324	8751.48	4566.35	2.38
Param3	136	235	191	10	●		18392.6	367015	22735.2	100.00

#NC 3 (TL / CL)

Name	Start	Stop	Pos	Tol.	Ctr	RefRange	Height	Area	AbsH	Ratio
Param1	24	75	41	10	○		1064.44	20712.7	5535.03	4.38
Param3	138	224	183	10	●		23241.7	472629	27732.2	100.00

#1 (TL / CL)

Name	Start	Stop	Pos	Tol.	Ctr	RefRange	Height	Area	AbsH	Ratio
Param1	21	82	43	10	○		4786.87	89494.2	9045.3	33.84
Param3	123	229	184	10	●		12926.4	264480	17137.3	100.00

#2 (TL / CL)

Name	Start	Stop	Pos	Tol.	Ctr	RefRange	Height	Area	AbsH	Ratio
Param1	21	82	43	10	○		589.97	11168.7	5170.11	4.52
Param3	123	229	184	10	●		13178.5	247280	18146.8	100.00

#3 (TL / CL)

Name	Start	Stop	Pos	Tol.	Ctr	RefRange	Height	Area	AbsH	Ratio
Param1	24	75	41	10	○		1622.31	29643.3	6528.98	7.24
Param3	138	224	183	10	●		20210	409363	24721.9	100.00

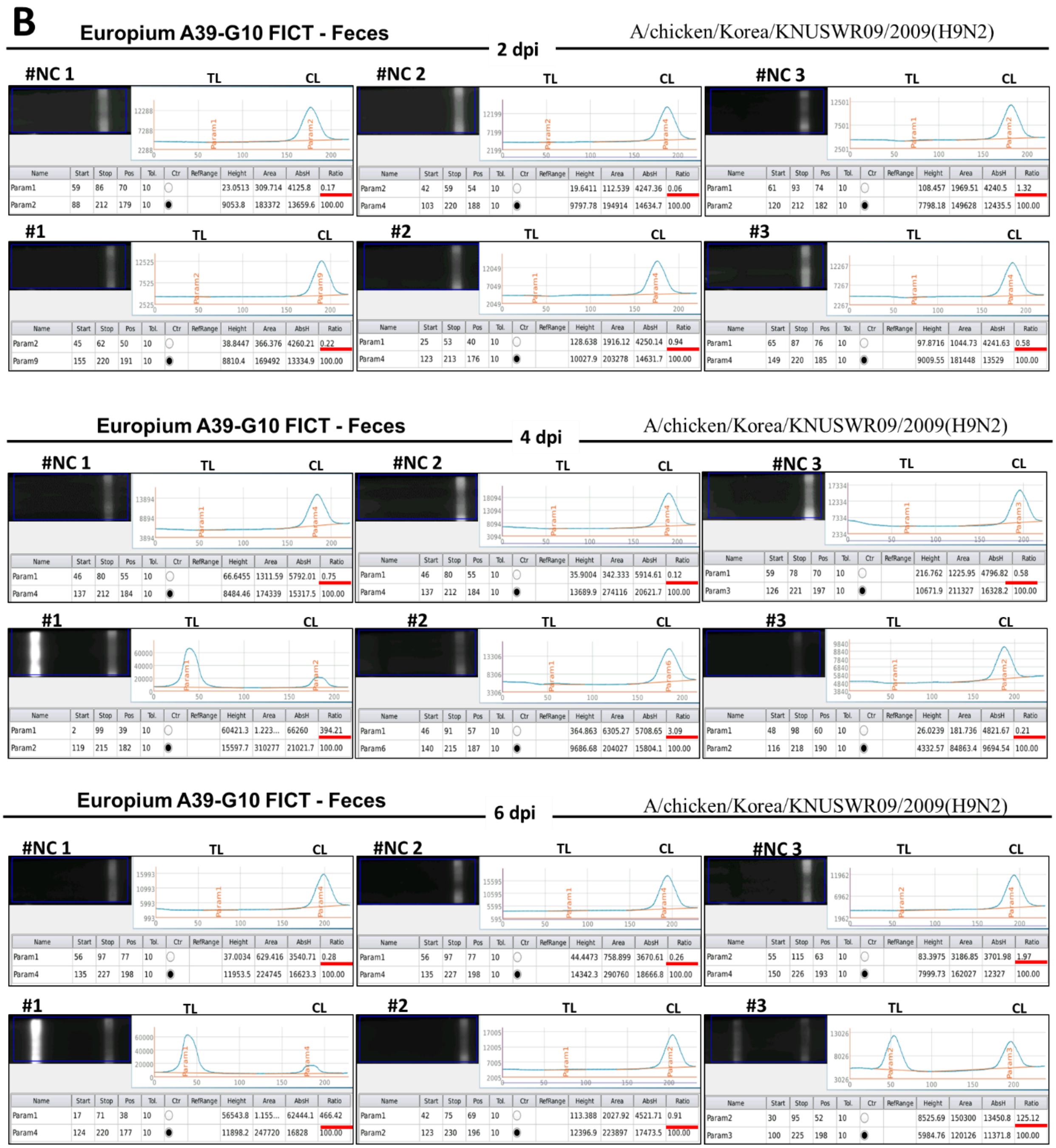
B
Europium A39-G10 FICT - Feces
2 dpi
A/chicken/Korea/KNUSWR09/2009(H9N2)
#NC 1
TL
CL
Name Start Stop Pos Tol. Ctr RefRange Height Area AbsH Ratio
Param1 59 86 70 10 23.0513 309.714 4125.8 0.17
Param2 88 212 179 10 9053.8 183372 13659.6 100.00
#NC 2
Param2 42 59 54 10 19.6411 112.539 4247.36 0.06
Param4 103 220 188 10 9797.78 194914 14634.7 100.00
#NC 3
Param1 61 93 74 10 108.457 1969.51 4240.5 1.32
Param2 120 212 182 10 7798.18 149628 12435.5 100.00
#1
Param2 45 62 50 10 38.8447 366.376 4260.21 0.22
Param9 155 220 191 10 8810.4 169492 13334.9 100.00
#2
Param1 25 53 40 10 128.638 1916.12 4250.14 0.94
Param4 123 213 176 10 10027.9 203278 14631.7 100.00
#3
Param1 65 87 76 10 97.8716 1044.73 4241.63 0.58
Param4 149 220 185 10 9009.55 181448 13529 100.00
Europium A39-G10 FICT - Feces
4 dpi
A/chicken/Korea/KNUSWR09/2009(H9N2)
#NC 1
Param1 46 80 55 10 66.6455 1311.59 5792.01 0.75
Param4 137 212 184 10 8484.46 174339 15317.5 100.00
#NC 2
Param1 46 80 55 10 35.9004 342.333 5914.61 0.12
Param4 137 212 184 10 13689.9 274116 20621.7 100.00
#NC 3
Param1 59 78 70 10 216.762 1225.95 4796.82 0.58
Param3 126 221 197 10 10671.9 211327 16328.2 100.00
#1
Param1 2 99 39 10 60421.3 1.223... 66260 394.21
Param2 119 215 182 10 15597.7 310277 21021.7 100.00
#2
Param1 46 91 57 10 364.863 6305.27 5708.65 3.09
Param6 140 215 187 10 9686.68 204027 15804.1 100.00
#3
Param1 48 98 60 10 26.0239 181.736 4821.67 0.21
Param2 116 218 190 10 4332.57 84863.4 9694.54 100.00
Europium A39-G10 FICT - Feces
6 dpi
A/chicken/Korea/KNUSWR09/2009(H9N2)
#NC 1
Param1 56 97 77 10 37.0034 629.416 3540.71 0.28
Param4 135 227 198 10 11953.5 224745 16623.3 100.00
#NC 2
Param1 56 97 77 10 44.4473 758.899 3670.61 0.26
Param4 135 227 198 10 14342.3 290760 18666.8 100.00
#NC 3
Param2 55 115 63 10 83.3975 3186.85 3701.98 1.97
Param4 150 226 193 10 7999.73 162027 12327 100.00
#1
Param1 17 71 38 10 56543.8 1.155... 62444.1 466.42
Param4 124 220 177 10 11898.2 247720 16828 100.00
#2
Param1 42 75 69 10 113.388 2027.92 4521.71 0.91
Param2 123 230 196 10 12396.9 223897 17473.5 100.00
#3
Param2 30 95 52 10 8525.69 150300 13450.8 125.12
Param3 100 225 198 10 5984.76 120126 11371.8 100.00

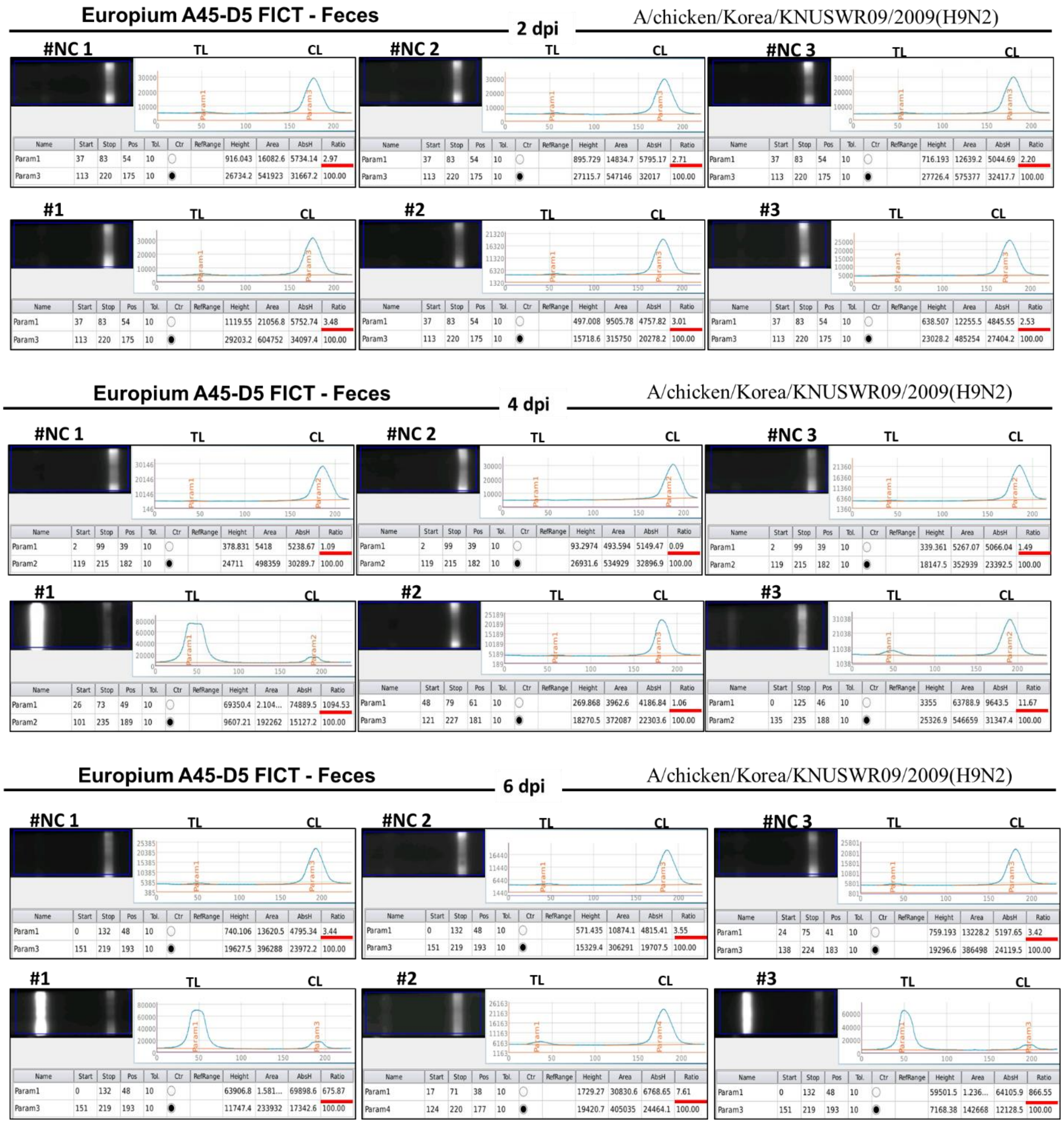

Fig.S14. Raw data of Europium A45-D5-FICT and Europium A39-G10 FICT using clinical specimens from SPF chickens at different day post-infection with A/chicken/Korea/KNUSWR09/2009(H9N2) strain. (A) Cloacal swabs, (B) feces samples. TL/CL is underlined with red color.

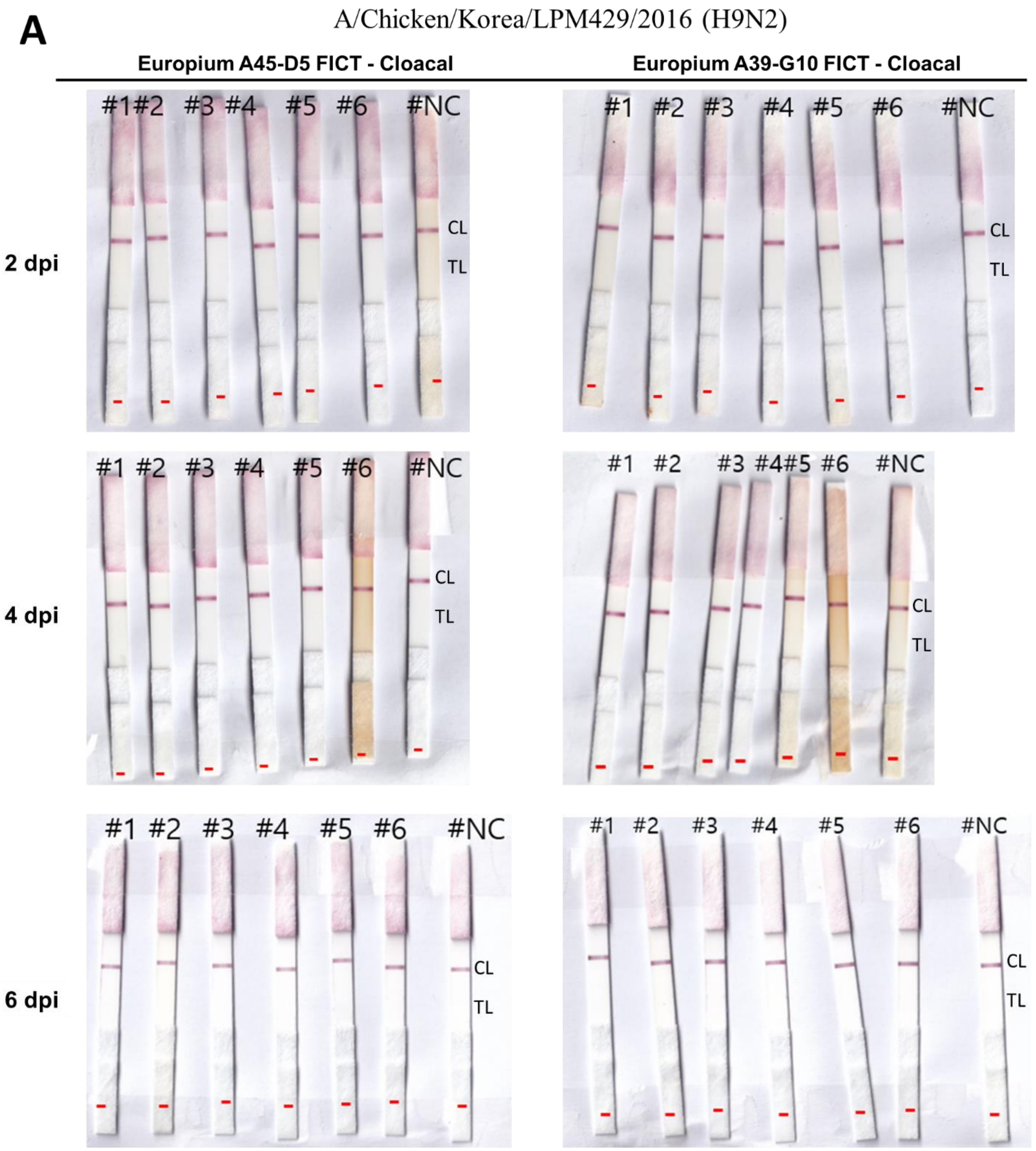
A
A/Chicken/Korea/LPM429/2016 (H9N2)
Europium A45-D5 FICT - Cloacal
Europium A39-G10 FICT - Cloacal
2 dpi
4 dpi
6 dpi
#1 #2 #3 #4 #5 #6 #NC
CL
TL

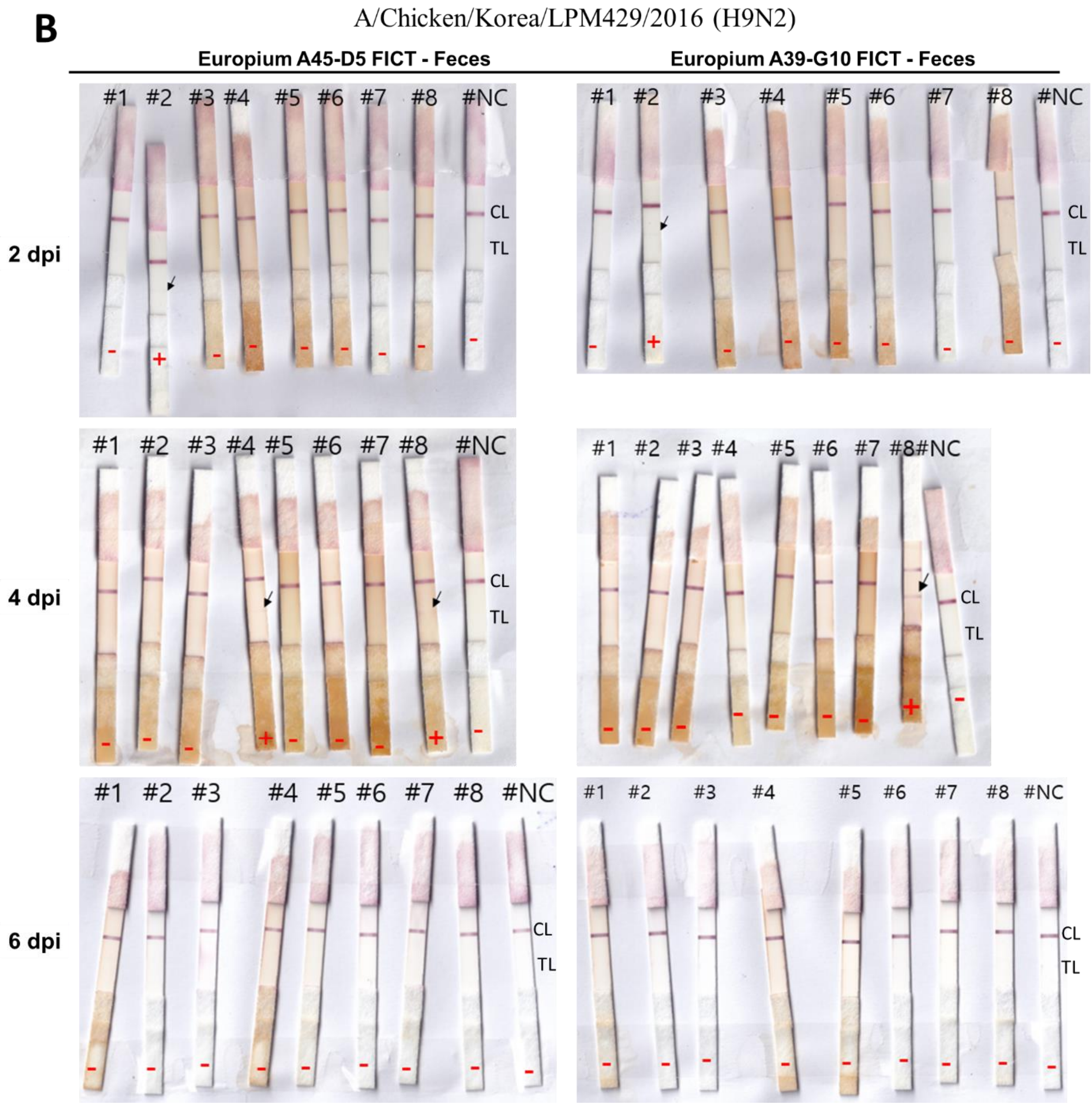

Fig.S15. RDT with clinical specimens from SPF chickens at different day post-infection with A/Chicken/Korea/LPM429/2016(H9N2) strain. (A) Cloacal swabs, (B) feces samples. TL, test line of influenza A H9N2; CL, control line

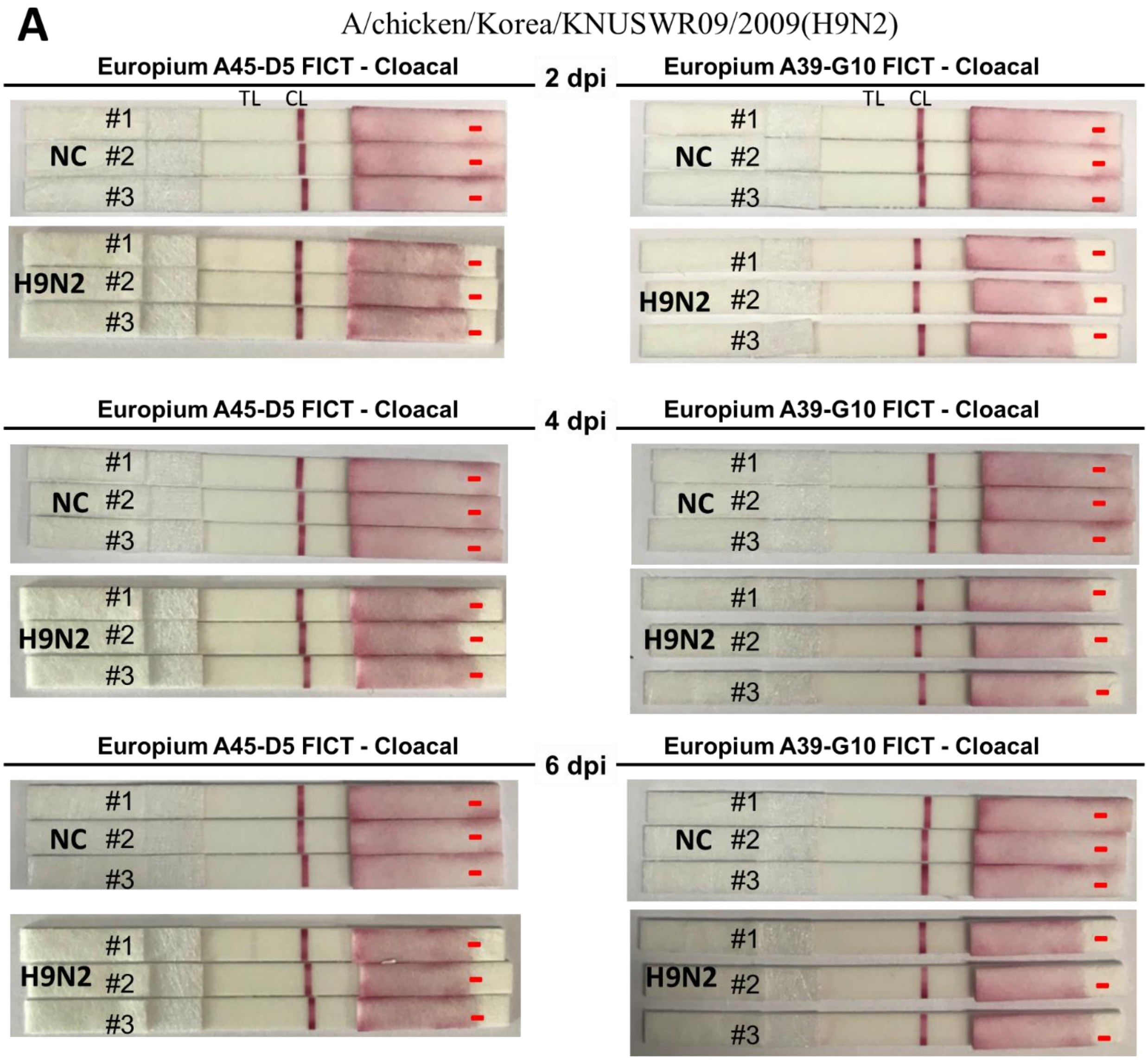
A
A/chicken/Korea/KNUSWR09/2009(H9N2)
Europium A45-D5 FICT - Cloacal
2 dpi
Europium A39-G10 FICT - Cloacal
TL
CL
NC
#1
#2
#3
H9N2
4 dpi
6 dpi

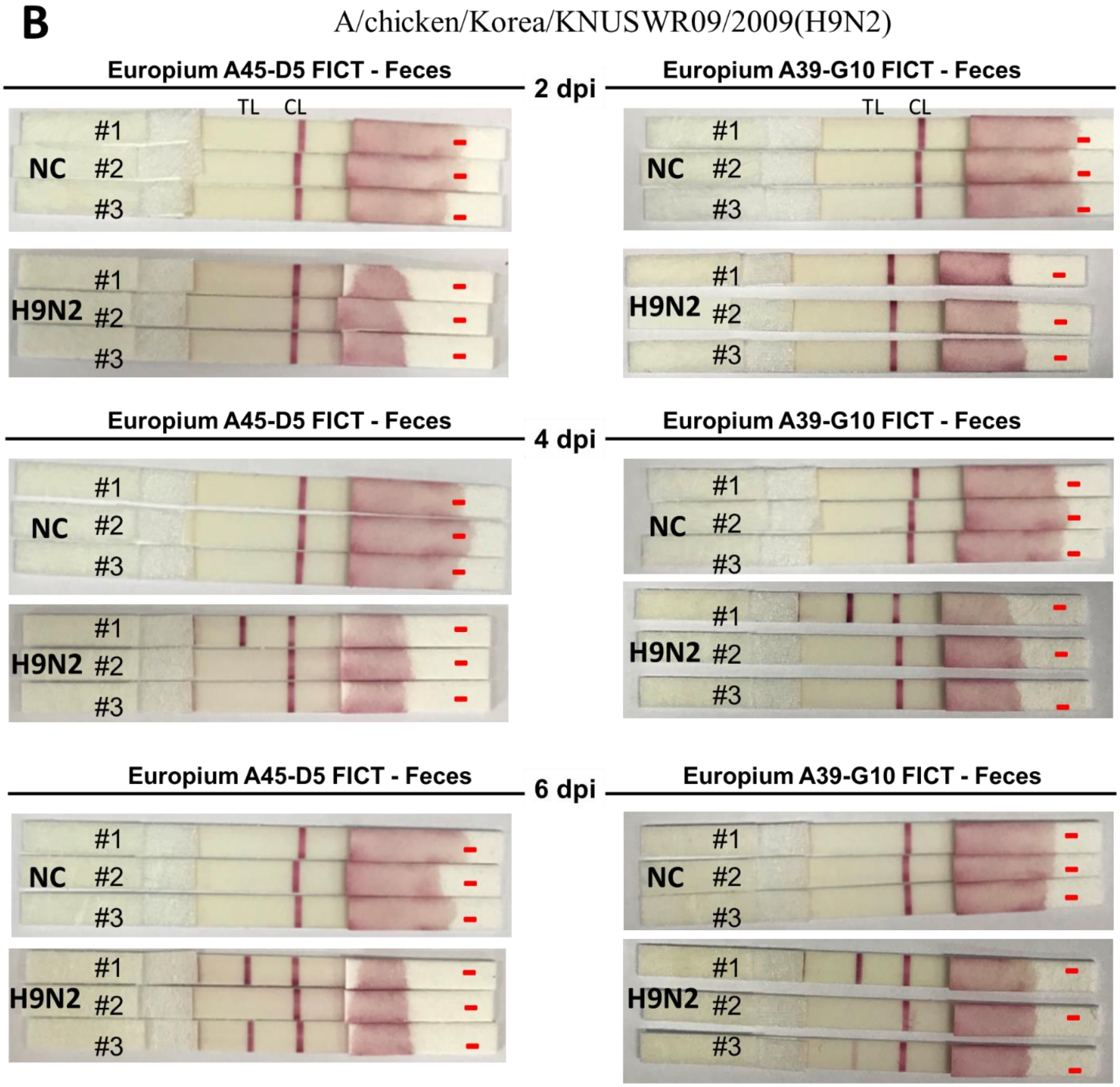

Fig.S16. RDT with clinical specimens from SPF chickens at different day post-infection with A/chicken/Korea/KNUSWR09/2009(H9N2) strain. (A) Cloacal swabs, (B) feces samples. TL, test line of influenza A H9N2; CL, control line

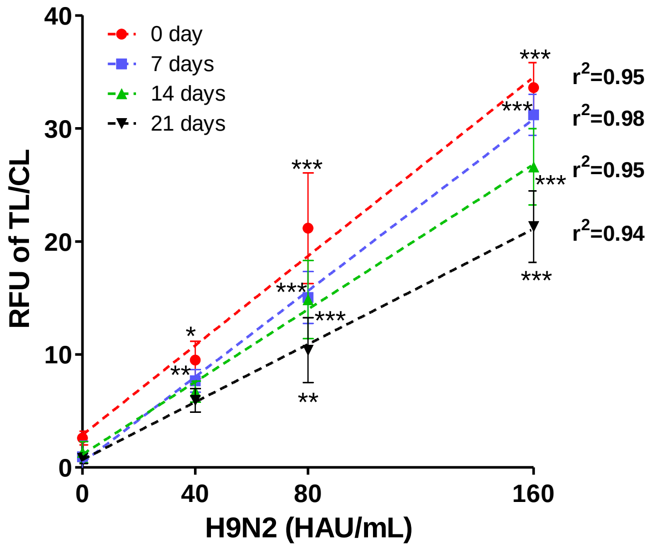

0 day
7 days
14 days
21 days
RFU of TL/CL
H9N2 (HAU/mL)
0
40
80
160
10
20
30
40
r2=0.95
r2=0.98
r2=0.95
r2=0.94
***
**
*

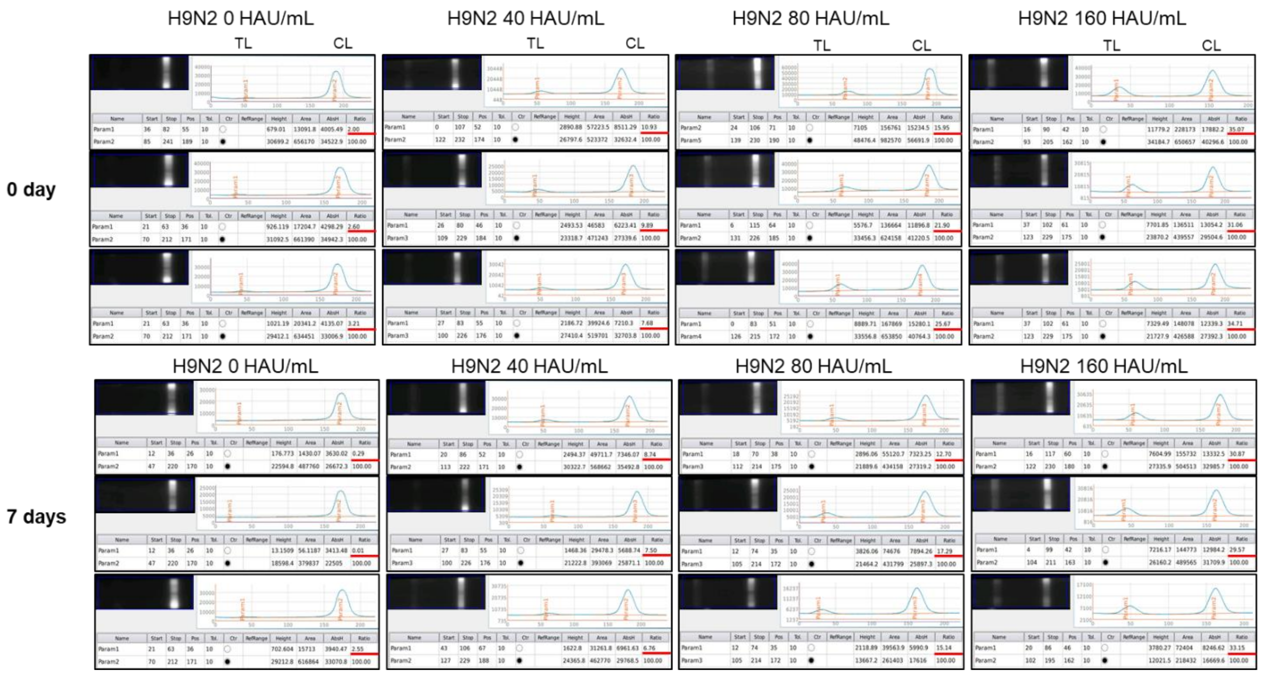

H9N2 0 HAU/mL
H9N2 40 HAU/mL
H9N2 80 HAU/mL
H9N2 160 HAU/mL
TL
CL
0 day
7 days

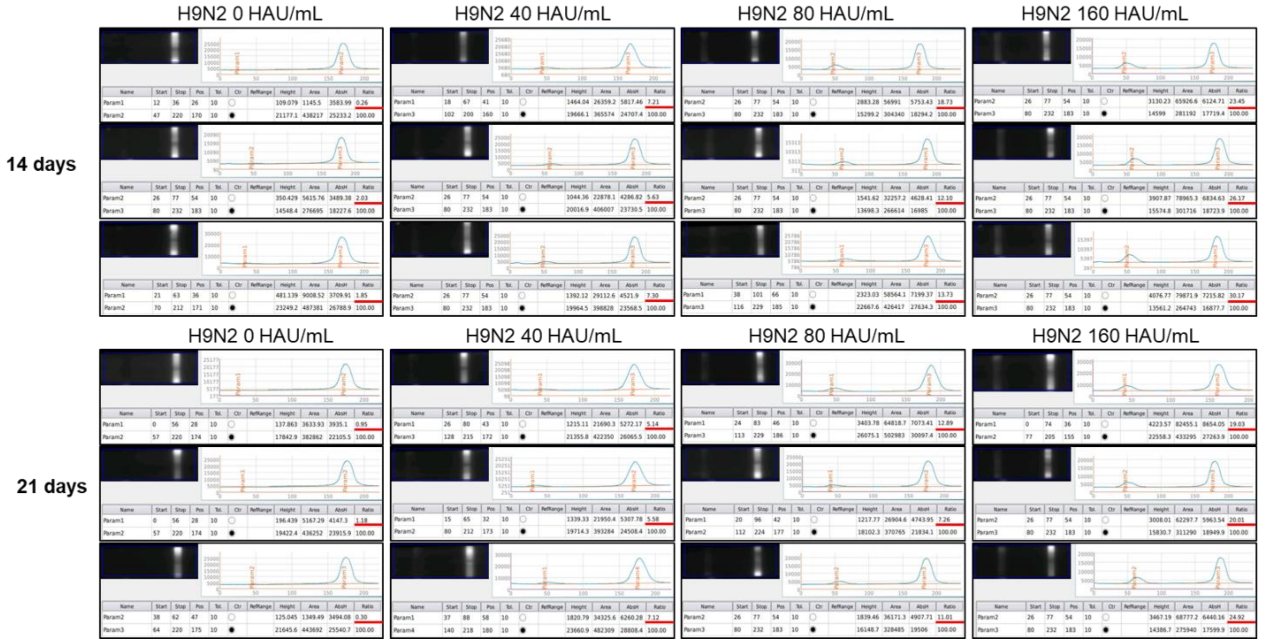

Fig.S17. Duration of FICT in dry condition. Two-fold serially diluted spiked-H9N2 (A/chicken/Korea/KNUSWR09/2009(H9N2)) virus in chicken feces was tested using 0-, 7-, 14-, 21-day-old conjugates. *, $P < 0.05$; **, $P < 0.01$; ***, $P < 0.001$.

Dear editor of MDPI,

Juwon Jeong, Younjeong Kim, Suyeon Yoon, and Gumju Bae in Wonkwang university endorse that they supported the documentation to perform this work and their names are written in Acknowledgement section of this article.:

Article title: Development of a Rapid Fluorescent Diagnostic System to Detect Subtype H9 Influenza A Virus in Chicken Feces.

Date: August 13, 2021

Juwon Jeong,

Younjeong Kim,

Suyeon Yoon,

Gumju Bae
